# Synthesis of α-D-Gal*p*N_3_-(1-3)-D-Gal*p*N_3_: α- and 3-*O-*selectivity using 3,4-diol acceptors

**DOI:** 10.3762/bjoc.14.258

**Published:** 2018-11-08

**Authors:** Emil Glibstrup, Christian Marcus Pedersen

**Affiliations:** 1 Department of Chemistry, University of Copenhagen, Universitetsparken 5, 2100 Copenhagen O, Denmark

**Keywords:** diastereoselectivity, glycosylation, regioselectivity

## Abstract

The motif α-D-Gal*p*NAc-(1-3)-D-Gal*p*NAc is very common in Nature and hence its synthesis highly relevant. The synthesis of its azido precursor has been studied and optimized in terms of steps, yields and selectivity. It has been found that glycosylation of the 3,4-diol acceptor is an advantage over the use of a 4-*O*-protected acceptor and that both regio- and anomeric selectivity is enhanced by bulky 6-*O*-protective groups. The acceptors and donors are made from common building blocks, limiting protective manipulations, and in this context, unavoidable side reactions.

## Introduction

The disaccharide α-D-Gal*p*NAc-(1-3)-β-D-Gal*p*NAc is a widespread motif in glycobiology and present in several distinct entities. One of the most studied glycoconjugates, containing this sequence, is the Forssman antigen, in which this disaccharide is the terminal unit. The many biological roles attributed to this particular glycoconjugate and its appearance in some human tumors [1–2] has triggered the interest of carbohydrate chemists since the early days of complex oligosaccharides synthesis. The structure of the pentasaccharide part of the Forssman antigen was resolved in the seventies [3–4] and soon thereafter Paulsen and Bünsch finished the chemical synthesis of the pentasaccharide chain [5–6]. The pioneering work by Paulsen was later followed up by a total synthesis by the Ogawa group [7] and an oligosaccharide synthesis by the Magnusson group [8]. With the increasing understanding of glycobiology, the Forssman antigen has remained an interesting target for vaccine development [9] and further biological evaluations [10]. The disaccharide motif is also commonly found in viruses and bacteria. In bacteria, as an example, it has been found in pathogenic bacteria such as in *Salmonella* [11], *Shigella* [12], several *Burkholderia* [13], *Escherichia coli* [14], *Vibrio chlorae* [15], *Edwardsiella ictaluri* [16], *Enterrococcus* [17], *Proteus mirabilis* [18], and *Streptococcus* [19–22]. With the wide spread appearance of α-D-Gal*p*NAc-(1-3)-β-D-Gal*p*NAc in many pathogens, this motif has been synthesized many times for various purposes, e.g., as part of the lipopolysaccharide found in *Shigella dysenteriae* [23], as derivatives of the mucin *O*-glycan core structures for glycosidase studies [24], for the synthesis of T-antigen analogues [25], for the synthesis of *E. coli O*-antigens [26–29], for the development of *Burkholderia* vaccines [30–32], for the synthesis of PS A1 conjugate vaccine [33], and the synthesis of *E. faecium* wall teichoic acid fragments for vaccine development [34–35]. We have been interested in α-D-Gal*p*NAc-(1-3)-β-D-Gal*p*NAc as a part of our ongoing syntheses of *S. pneumoniae* lipoteichoic acid (LTA) derivatives [20,36–37]. During optimization of the synthesis, we realized that the synthesis of the α-D-Gal*p*NAc-(1-3)-β-D-Gal*p*NAc part worked more efficiently, when using the 3,4-diol acceptor instead of a fully protected 3-OH. As some protective group manipulations can be avoided and at the same time the yield and α-selectivity improved, we consider this finding important for oligosaccharide synthesis. Here we describe this regioselective glycosylation approach in detail.

## Results and Discussion

**Scheme 1:**
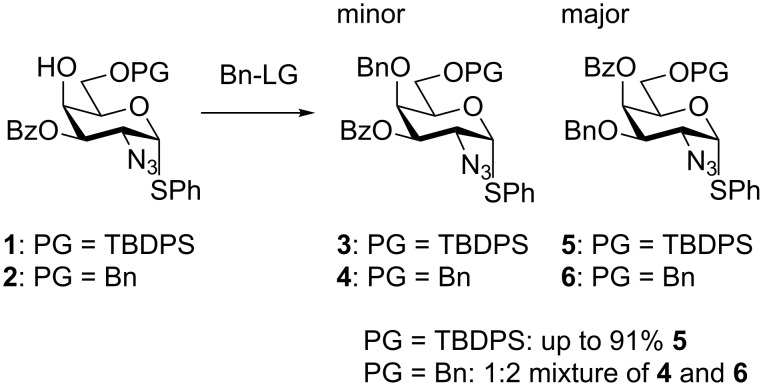
Undesired migration followed by benzylation of the 3-*O*-Bz GalN_3_ using several different benzylation procedures (LG: leaving group).

In the initial strategy for synthesizing the disaccharide, the fully protected acceptor (**1** or **2**) was intended to be a key building block. However, an unexpected problem during its preparation occurred, i.e., benzylation of the axial 4-OH only gave the desired 4-*O*-Bn product as a minor product, whereas the benzoyl migration from the 3-*O* to the 4-*O*, followed by benzylation of the 3-*O*, was the major, if not the exclusive, product. Despite several different benzylation procedures, i.e., NaH and BnBr, TriBOT [38] and TfOH or BnBr and Ag_2_O, the 4-*O*-benzylation remained inaccessible, with a 3-*O*-benzoyl group present. Using the 6-*O*-Bn-protected variant (**2** in Scheme 1), a 1:2 mixture of the desired vs migrated product could be obtained. When using the more bulky 6-OTBDPS as protective group (**1** in Scheme 1) and benzylation with freshly prepared Ag_2_O and BnBr in the solvent mixture CH_2_Cl_2_/cyclohexane 1:4 as described by Wang et al. [39], a 91% isolated yield of the migrated product, i.e., 3-*O*-Bn, 4-*O*-Bz, was obtained (Scheme 1).

Following the apparent difference in nucleophilicity and accessibility of the axial 4-OH versus the equatorial 3-OH [40], we wondered, if this could be used to simplify the synthesis of the α-D-Gal*p*N_3_-(1-3)-D-Gal*p*N_3_ unit. When consulting the literature, surprisingly few reports were describing diol glycosylations targeting the *galacto-*configured sugar [41–43] and none for the synthesis of the α-D-Gal*p*NAc-(1-3)-D-Gal*p*NAc motif or its precursors.

We therefore set out to study this regioselective glycosylation in more detail and synthesized the necessary model donors and acceptors. Here, the simplicity of the approach showed its potential, as both, donor and acceptor, could be obtained from a common late stage building block, that in turn could be synthesized anomerically pure as described previously [44]. Alternatively, these components could be obtained via an azido phenylselenylation approach starting from D-galactose [45–46].

**Scheme 2:**
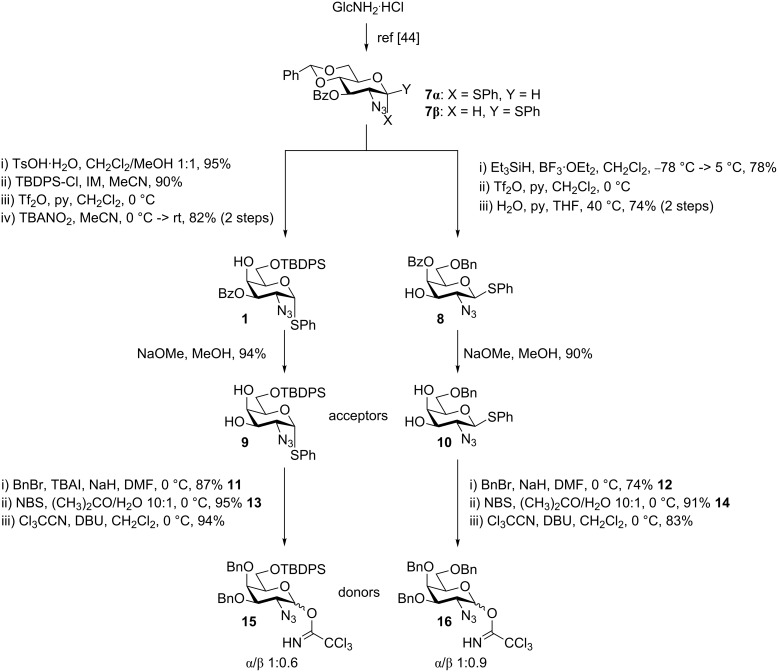
Simple synthesis of two acceptors and two donors from the same common and readily available building block.

Starting from the common 2-azidoglucose precursor **7**, the 4,6-*O*-benzylidene group was removed with TsOH to give the 4,6-diol in 95% yield, followed by selective 6-*O*-protection using TBDPS-Cl and imidazole (IM) in 90% yield. The 4,6-*O*-benzylidene motif could also be regioselectively opened using triethylsilane and BF_3_∙OEt_2_ to achieve the 6-*O*-Bn-protected variant in 78% yield (Scheme 2).

In order to invert the 4-OH, to achieve the *galacto* configuration, two approaches were attractive: one using our recently published method [44] or alternatively the Latrell–Dax inversion using nitrite [47–48]. When having the 6-*O*-TBDPS protection the Latrell–Dax inversion provided slightly higher yields of **1**, whereas with the 6-*O*-Bn **8**, the two methods provided comparable yields. Migration of the benzoyl group under the 4-*O* inversion conditions was irrelevant as it is removed in the following step. The proposed acceptors were obtained via Zemplén deprotection in 94% and 90% yield for the 6-*O*-TBDPS **9** and 6-*O*-Bn **10**, respectively (Scheme 2). The acceptors could in turn be transformed into the corresponding donors in three simple steps by first benzylating the 3,4-diol (**11** and **12**), hydrolyzing the thiophenol using NBS (**13** and **14**), thereby making anomeric purity inconsequential, and finally formation of the donors, **15** and **16**, as trichloroacetimidates [49]. With both, the donors and acceptors in hand, we set out to test our hypothesis of regioselective 3-*O-*α-glycosylation on the 3,4-diol **9**, which is based on the superior nucleophilicity and accessibility of the 3-OH compared to the axial 4-OH. Initially, a small screening of the glycosylation conditions expected to affect the selectivity was performed.

As the donor **15** stems from the acceptor **9**, it was decided to use a slight excess (1.2 equiv) of the “less precious” and recoverable acceptor **9**, which, however, would result in lower yields when compared to glycosylations using excess of donor. In the initial experiment, using TMSOTf as the catalyst in CH_2_Cl_2_ at 0 °C, a pleasingly 8:1 3/4-*O* ratio and a 10:1 α/β-selectivity with a 57% isolated yield of the desired disaccharide **17**, was obtained. Comparing the “normal procedure” with the “inverse procedure” [50], i.e., slow addition of donor **15** to acceptor **9** and catalyst, no discernable difference in the 3/4-*O*-regioselectivity or the α/β-ratio was observed, albeit a slightly lower isolated yield was obtained using the inverse procedure (Table 1, entry 1 vs entry 2). Changing the temperature, i.e., room temperature, 0 °C and −50 °C, had no effects on the 3/4-*O-*ratio, but resulted in a lower α/β-selectivity (5:1 at −50 °C; Table 1, entries 3 and 4) [51]. The reaction was also shown to be scalable giving comparable ratios and excellent isolated yields at both 1 mmol and 2.6 mmol scales (Table 1, entries 5 and 6). Increasing the excess of acceptor **9** to 1.7 equiv, showed no effect towards the ratios observed (Table 1, entry 6).

**Table 1:** Optimizing the α,3-*O*-selective glycosylation conditions.

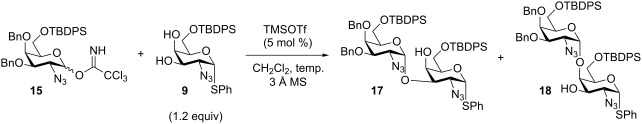					

entry	scale [mmol]	temp.	3/4-*O*^a^	α/β^a^	isolated yields

1	0.1	0 °C	8:1	10:1	**17** (57%)
2^c^	0.1	0 °C	7:1	11:1	**17** (47%) **18** (9%)
3	0.1	−50 °C	7:1	5:1	**17** (51%) **18** (10%)
4	0.1	rt	7:1	10:1	**17** (66%) **18** (11%)
5	1.0	0 °C	7:1	12:1	**17** (67%) **18** (9%)
6^b^	2.6	0 °C	8:1	11:1	**17** (64%) **18** (9%)

^a^Determined by ^1^H NMR on the crude; ^b^1.7 equiv acceptor; ^c^inverse procedure.

**Scheme 3:**
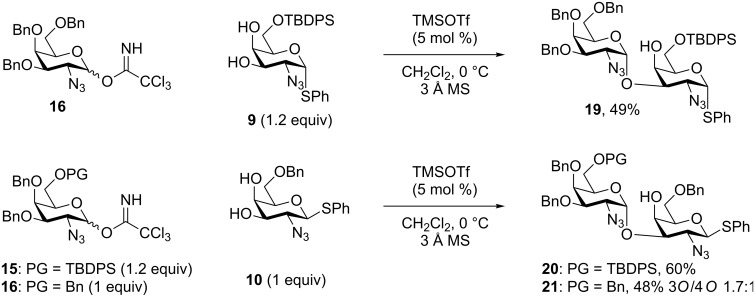
Challenging the α,3-*O*-selectivity with the different 6-*O*-protecting group variants.

**Scheme 4:**
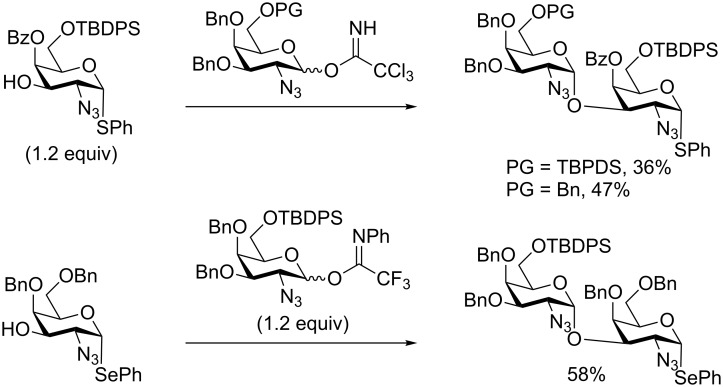
Representative glycosylations with closely related systems [34,44].

With the conditions optimized, the selectivity was challenged using less bulky 6-*O-*protecting groups, such as a benzyl group, at either the donor, the acceptor or at both. With the 6-*O*-Bn donor **16** a slightly diminished, but still respectable, 49% yield of the desired product **19** was obtained (Scheme 3). However, when using the 6-*O*-TBDPS donor **15** (1.2 equiv) a 60% isolated yield of the desired disaccharide **20** was obtained. When using both the 6-*O*-Bn donor **10** and acceptor **16** we saw a lower selectivity than observed with the TBDPS–TBDPS variant **17**, with an isolated yield of 48% of **21**, but as an inseparable 1.7:1 mixture of the 3/4-*O*-glycosylated products. To put these results in to perspective, a few closely related glycosylations are shown in Scheme 4. Firstly, our own glycosylations using the 4-*O*-Bz variants, gave a lower yield, when using the TBDPS–TBDPS variant, of 36% [44]. The less sterically hindered Bn–TBDPS variant gave yields of 47%, which are comparable to the 3,4-diol glycosylation. Glycosylation with a closely related system having a 4-*O*-Bn, 6-*O*-TBDPS pattern gave 58% yield using 1.2 equiv of the donor, proving very comparable to our 60% yield for the same 4-*O*-Bn, 6-*O*-TBDPS system (Scheme 3) [34]. If utilizing the 3,4-diol glycosylation herein reported, the acceptor **10** can be synthesized in 4 less steps than otherwise required.

These results show, how the desired protecting group pattern can direct which glycosylation strategy to choose: In the less sterically hindered cases, a 4-*O*-protecting group, such as the benzoyl or benzyl, can be preferable. However, when a large 6-*O*-protective group, such as TBDPS, is used, it proves advantageous to do the glycosylation directly on the 3,4-diol.

**Scheme 5:**
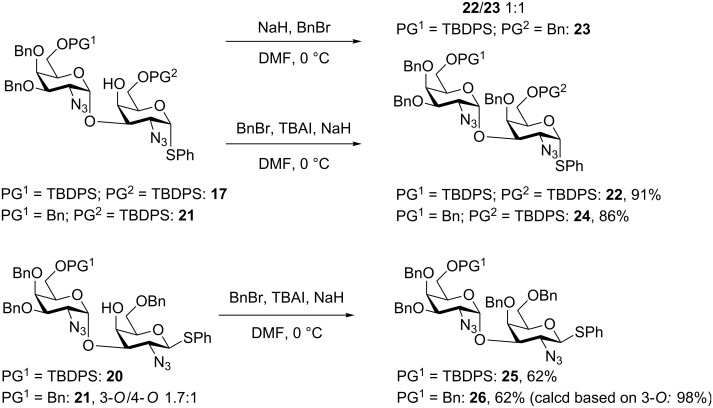
Capping the free 4-OH, allowing for easier separation of mixtures obtained during glycosylation.

Post-glycosylation modifications, i.e., benzylation of the free 4-OH on the TBDPS–TBDPS variant **17** using standard BnBr followed by NaH resulted in a 1:1 mixture of the desired product **22** and a side-product **23**, in which the adjacent TBDPS has been cleaved off and the 6-OH subsequently benzylated (Scheme 5). This side reaction could, however, easily be avoided by using a procedure in which BnBr and TBAI was added and mixed before the addition of NaH. This modification gave a 91% yield of the product **22**. Employing these conditions to the other three variants **24**–**26** allowed for much easier separation of the 3/4-*O* mixtures obtained from the glycosylations, especially for the Bn–Bn variant **26**.

**Scheme 6:**
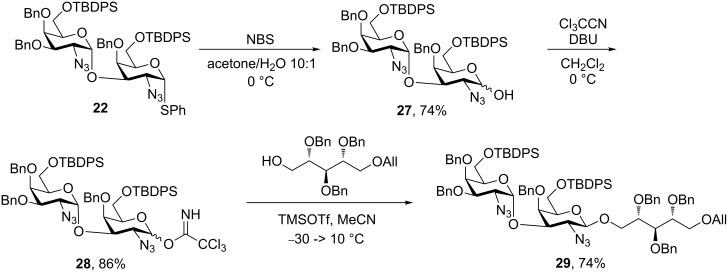
Pseudotrisaccharide synthesis for LTA elucidation.

Ultimately, we employed the strategy to synthesize a pseudotrisaccharide, found as the terminal part of the LTA from *S. pneumoniae*. The deprotection of disaccharide **22** to give **27** followed by formation of the trichloroacetimidate **28** proceeded smoothly, as shown in Scheme 6. The following glycosylation using MeCN as solvent relied on the nitrile effect to afford the desired β-anomer **29** in 74% yield.

## Conclusion

In conclusion we have shown, how the difference in nucleophilicity along with steric effects can be utilized to employ an unprecedented regioselective glycosylation on a 3,4-diol, resulting in up to 67% isolated yield. Following general guidelines can be given:

- With bulky 6-*O*-protective groups, a 4-*O*-protective group should be avoided, i.e., use the diol acceptor.
- Bulky 6-*O*-protective groups on the donor enhance the α-selectivity.
- Bulky 6-*O*-protective groups on either the donor or the acceptor enhance the 3-*O*-regioselectivity.

This simplification in synthesis allows for easier and faster access to these biologically abundant and relevant α-D-Gal*p*NAc-(1-3)-D-Gal*p*NAc motifs.

## Supporting Information

File 1 Full description of experimental procedures and characterization of new compounds.
