# Supplementary material for: Synthesis of α-D-GalpN3-(1-3)-D-GalpN3: α- and 3-O-selectivity using 3,4-diol acceptors
Source: Beilstein J Org Chem. 2018 Nov 8;14:2805–11. doi: 10.3762/bjoc.14.258 (PMC6244312; doi:10.3762/bjoc.14.258)
Supplement: File 1 — Full description of experimental procedures and characterization of new compounds. [file Beilstein_J_Org_Chem-14-2805-s001.pdf]

Supporting Information

for

Synthesis of α-D-Gal*p*N_3-(1-3)-D-Gal*p*N_3: α- and 3-*O*-selectivity using 3,4-diol acceptors

Emil Glibstrup and Christian Marcus Pedersen*

Address: Department of Chemistry, University of Copenhagen, Universitetsparken 5, 2100 Copenhagen O, Denmark

Email: Christian Marcus Pedersen - cmp@chem.ku.dk

*Corresponding author

Full description of experimental procedures and characterization of new compounds

Contents

S1 Experimentals **S3**

S1.1 General . . . S3

S1.2 Phenyl 2-deoxy-2-azido-3-*O*-benzoyl-6-*O*-*tert*-butyldiphenylsilyl-thio-α-D-galactopyranoside (**1**) . . . S4

S1.3 Phenyl 2-deoxy-2-azido-6-*O*-*tert*-butyldiphenylsilyl-thio-α-D-galactopyranoside, (**9**) . . . S4

S1.4 Phenyl 2-deoxy-2-azido-3,4-di-*O*-benzyl-6-*O*-*tert*-butyldiphenylsilyl-thio-α-D-galactopyranoside, (**11**) . . . S5

S1.5 2-deoxy-2-azido-3,4-di-*O*-benzyl-6-*O*-*tert*-butyldiphenylsilyl-α/β-D-galactopyranose, (**13**) . . . S6

S1.6 *O*-(2-deoxy-2-azido-3,4-di-*O*-benzyl-6-*O*-*tert*-butyldiphenylsilyl-α/β-D-galactopyranosyl) Trichloroacetimidate, (**15**) . . . S6

S1.7 Phenyl 2-deoxy-2-azido-6-*O*-benzyl-thio-β-D-galactopyranoside, (**10**) . . . S7

S1.8 Phenyl (2-deoxy-2-azido-3,4,6-tri-*O*-benzyl-thio-α/β-D-galactopyranoside, (**12**α/β) S7

S1.9 2-deoxy-2-azido-3,4,6-tri-*O*-benzyl-α/β-D-galactopyranose, (**14**) . . . S8

S1.10 *O*-(2-deoxy-2-azido-3,4, 6-tri-*O*-benzyl-α/β-D-galactopyranosyl) Trichloroacetimidate, (**16**) . . . S8

S1.11 Phenyl (2-deoxy-2-azido-3,4-di-*O*-benzyl-6-*O*-*tert*-butyldiphenylsilyl-α-D-galactopyranosyl)(1→3)-2-deoxy-2-azido-6-*O*-*tert*-butyldiphenylsilyl-thio-α-D-galactopyranoside, (**17**) . . . S9

S1.12 Phenyl (2-deoxy-2-azido-3,4,6-tri-*O*-benzyl-α-D-galactopyranosyl)(1→3)-2-deoxy-2-azido-6-*O*-*tert*-butyldiphenylsilyl-thio-α-D-galactopyranoside, (**19**) . . . S10

S1.13 Phenyl (2-deoxy-2-azido-3,4-di-*O*-benzyl-6-*O*-*tert*-butyldiphenylsilyl-α-D-galactopyranosyl)(1→3)-2-deoxy-2-azido-6-*O*-benzyl-thio-β-D-galactopyranoside, (**20**) S11

S1.14 Phenyl (2-deoxy-2-azido-3,4,6-tri-*O*-benzyl-α-D-galactopyranosyl)(1→3)-2-deoxy-2-azido-4,6-di-*O*-benzyl-thio-β-D-galactopyranoside, (**26**) . . . S12

S1.15 Phenyl (2-deoxy-2-azido-3,4-di-*O*-benzyl-6-*O*-*tert*-butyldiphenylsilyl-α-D-galactopyranosyl)(1→3)-2-deoxy-2-azido-4-*O*-benzyl-6-*O*-*tert*-butyldiphenylsilyl-thio-α-D-galactopyranoside, (**22**) . . . S13

S1.16 Phenyl (2-deoxy-2-azido-3,4-di-*O*-benzyl-6-*O*-*tert*-butyldiphenylsilyl-α-D-galactopyranosyl)(1→3)-2-deoxy-2-azido-4-*O*-benzyl-6-*O*-benzyl-thio-α-D-galactopyranoside, (**23**) . . . S14

S1.17 Phenyl (2-deoxy-2-azido-3,4,6-tri-*O*-benzyl-α-D-galactopyranosyl)(1→3)-2-deoxy-2-azido-4-*O*-benzyl-6-*O*-*tert*-butyldiphenylsilyl-thio-α-D-galactopyranoside, (**24**) S15

S1.18 Phenyl (2-deoxy-2-azido-3,4-di-*O*-benzyl-6-*O*-*tert*-butyldiphenylsilyl-α-D-galactopyranosyl)(1→3)-2-deoxy-2-azido-4,6-di-*O*-benzyl-thio-β-D-galactopyranoside, (**25**) . . . S16

S1.19 2-deoxy-2-azido-3,4-di-*O*-benzyl-6-*O*-*tert*-butyldiphenylsilyl-α-D-galactopyranosyl)(1→3)-2-deoxy-2-azido-4-*O*-benzyl-6-*O*-*tert*-butyldiphenylsilyl-α/β-D-galactopyranose, (**27**) S17

S1.20 *O*-(2-deoxy-2-azido-3,4-di-*O*-benzyl-6-*O*-*tert*-butyldiphenylsilyl-α-D-galactopyranosyl)(1→3)-2-deoxy-2-azido-4-*O*-benzyl-6-*O*-*tert*-butyldiphenylsilyl-α/β-D-galactopyranosyl) Trichloroacetimidate, (**28**) . . . S18

S1.21 5-*O*-allyl-1-*O*-(3-*O*-[2-azido-3,4-di-*O*-benzyl-6-*O*-(*tert*-butyldiphenylsilyl)-2-deoxy-α-D-galactopyranosyl]-2-azido-4-*O*-benzyl-6-*O*-(*tert*-butyldiphenylsilyl)-2-deoxy-β-D-galactopyranosyl)-2,3,4-tri-*O*-benzyl-D-ribitol, (**29**) . . . S19

S2 Spectra **S20**

S2.1 Spectra for **1** . . . S20

S2.2 Spectra for **9** S24
S2.3 Spectra for **11** S27
S2.4 Spectra for **15** S32
S2.5 Spectra for **12**α/β S36
S2.6 Spectra for **17** S40
S2.7 Spectra for **19** S45
S2.8 Spectra for **20** S50
S2.9 Spectra for **26** S55
S2.10Spectra for **22** S60
S2.11Spectra for **23** S65
S2.12Spectra for **24** S70
S2.13Spectra for **25** S75
S2.14Spectra for **27** S80
S2.15Spectra for **28** S84
S2.16Spectra for **29** S88

List of References **S93**

S1 Experimentals

S1.1 General

NMR-spectra were acquired using a 500 MHz Avance III HD equipped with a cryogenically cooled 5 mm observe probe optimized for ^{13}C. Samples were dissolved in chloroform-d (D 99.8 %) or methanol-d_4 (D 99.8 %) and analyzed at 298 K. The spectra were referenced to the solvent peak; $CHCl_3$ ^{1}H 7.260 ppm and ^{13}C 77.160 ppm, MeOH ^{1}H 3.310 ppm and ^{13}C 49.000 ppm.

Flash column chromatography was performed with SiO_2 (LC60Å, 40-63 µm, pre-neutralized from the manufacturer to a pH of 6.0-8.0), direct loading with a pasteur pipette in minimal amount of eluent or evaporation onto celite and layered on top of the silica. Pressure was continuously applied with a hand pump. Solvents were of technical grade.

Solvents used in reactions were of HPLC grade and used without any further purification. Dry MeCN, CH_2Cl_2 and THF were obtained from a drying tower, other dry solvents used were dried with molecular sieves (4 Å for pyridine, 3 Å for MeOH). All Petroleum ether (PE) used was in the b.p. 40-65 °C range. Chemicals were generally obtained from the in-house chemical inventory or bought and used without further purification.

High-res ESI-MS and High-res MALDI-MS spectra were recorded with a SolariX XR 7 T ESI/MALDI-FT-ICR-MS instrument and data was handled with the DataAnalysis Version 4.0 SP 4. ESI samples were dissolved in MeCN or mixtures of MeCN and H_2O with 0.1% HCOOH. MALDI samples were dissolved in CH_2Cl_2 and applied with dithranol as matrix.

General glycosylation optimization procedure. Donor (0.1 mmol) and acceptor (0.12 mmol, 1.2 equiv.) were dissolved in dry CH_2Cl_2 (2 mL) and stirred with 3 Å molecular sieves for 2 h. The reaction mixture was then cooled to 0 °C before adding TMSOTf (5-10 mol%). The reaction was quenched after 15 min using Et_3N (0.1 mL), filtrated through a pad of celite and solvents were evaporated *in vacuo*. Crude ^{1}H NMR was used to determine the 3/4-*O* and α/β-ratio. The crude product was purified by flash column chromatography.

S1.2 Phenyl 2-deoxy-2-azido-3-*O*-benzoyl-6-*O*-*tert*-butyldiphenylsilyl-thio-α-D-galactopyranoside (1)

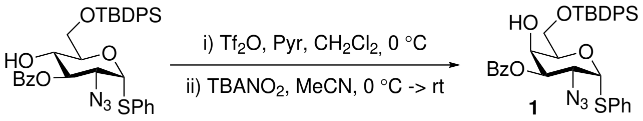

A solution of the *gluco*-configured 4-OH(Phenyl 2-deoxy-2-azido-3-*O*-benzoyl-6-*O*-*tert*-butyldiphenylsilyl-thio-α-D-glucopyranoside)[1] (5.75 g, 8.99 mmol) in dry CH_2Cl_2 (100 mL) was cooled to 0 °C, added dry pyridine (2.90 mL, 36.0 mL, 4 equiv.) followed by Tf_2O (2.30 mL, 13.7 mmol, 1.5 equiv.). The reaction was quenched after 30 min with 1 M HCl, washed with sat. aq $NaHCO_3$ followed by brine. The organic phase was dried using $MgSO_4$ followed by evaporation *in vacuo*. The crude triflate was redissolved in dry MeCN (100 mL) and cooled to 0 °C before addition of tetrabutylammonium nitrite ($TBANO_2$) (5.18 g, 18.0 mmol, 2 equiv.). The reaction was allowed to reach rt overnight, after which it was diluted with EtOAc, washed with H_2O, the water phase was then backextracted with more EtOAc and the combined organic phase was washed with brine, dried over $MgSO_4$ and evaporated *in vacuo*. The crude product was purified by flash column chromatography (SiO_2, CH_2Cl_2:PE 9:1) to yield the pure title compound **1** (4.73 g, 7.39 mmol, 82%)(R_f 0.59 CH_2Cl_2)

Characterisation

^{1}H NMR (500 MHz, $CDCl_3$) δ 8.16-8.13 (m, 2H), 7.70-7.65 (m, 4H), 7.61 (tt, J = 7.2, 1.3 Hz, 1H), 7.51-7.48 (m, 2H), 7.48-7.42 (m, 5H), 7.41-7.34 (m, 5H), 7.26-7.24 (m, 2H), 5.79 (d, J = 5.5 Hz, 1H, H-1), 5.28 (dd, J = 11.0, 2.8 Hz, 1H, H-3), 4.74 (dd, J = 11.0, 5.5 Hz, 1H, H-2), 4.59 (d, J = 2.4 Hz, 1H, H-4), 4.45 (t, J = 4.1 Hz, 1H, H-5), 3.95 (d, J = 4.1 Hz, 2H, H-6), 3.51 (br s, 1H, OH-4), 1.05 (s, 9H).

^{13}C NMR (125 MHz, $CDCl_3$) δ 165.8 (C=O), 135.8, 135.6, 133.7, 133.6, 132.6, 132.3, 132.26, 130.16, 130.17, 130.14, 129.4, 129.2, 128.7, 128.1, 128.0, 127.7 (24 C-Ar), 87.8 (C-1), 73.8 (C-3), 70.0 (C-5), 69.0 (C-4), 65.0 (C-6), 58.6 (C-2), 26.9 (tBu-Me), 19.2 (tBu-C).

HRMS (MALDI) m/z: $[M + Na]^+$ Calcd for $C_{35}H_{37}N_3O_5SSiNa^+$ 662.2115; Found 662.2104.

$[\alpha]_D^{25}$ +99.6 (c 0.47, $CHCl_3$)

Spectra can be seen on page S20 - S23.

S1.3 Phenyl 2-deoxy-2-azido-6-*O*-*tert*-butyldiphenylsilyl-thio-α-D-galactopyranoside, (9)

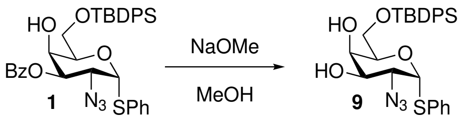

The benzoyl ester **1** (4.73 g, 7.39 mmol) was dissolved in dry MeOH (75 mL) and added NaOMe (0.10 mL, 25% in MeOH, 0.44 mmol, 6 mol%). After 30 min the reaction mixture was

neutralized using Amberlite resin (H^+) followed by filtration and evaporation *in vacuo*. The crude product was purified by flash column chromatography (SiO_2, short, CH_2Cl_2) to yield the pure title compound **9** (3.72 g, 6.94 mmol, 94%)(R_f 0.16 CH_2Cl_2) as a white foam.

Characterisation

Spectral data (peak assignment) were not in full agreement with literature.[2]
^{1}H NMR (500 MHz, $CDCl_3$) δ 7.72-7.65 (m, 4H), 7.48-7.35 (m, 9H), 7.25-7.22 (m, 2H), 5.66 (d, J = 5.5 Hz, 1H, H-1), 4.35 (t, J = 4.4 Hz, 1H, H-5), 4.24 (br s, 1H, H-4), 4.20 (dd, J = 10.4, 5.5 Hz, 1H, H-2), 3.94 (d, J = 4.5 Hz, 1H, H-6), 3.84 (ddd, J = 10.8, 8.7, 3.2 Hz, 1H, H-3), 3.54 (br s, 1H, OH-4), 2.67 (d, J = 8.3 Hz, 1H, OH-3), 1.07 (s, 9H).

^{13}C NMR (125 MHz, $CDCl_3$) δ 135.8, 135.6, 133.8, 132.7, 132.4, 131.9, 130.2, 130.1, 129.2, 128.1, 128.0, 127.6 (18 C-Ar), 87.6 (C-1), 70.8, 70.17, 70.16, 64.6, 61.5, 26.9 (*t*Bu-Me), 19.3 (*t*Bu-C).

Spectra can be seen on page S24 - S26.

S1.4 Phenyl 2-deoxy-2-azido-3,4-di-*O*-benzyl-6-*O*-*tert*-butyldiphenylsilyl-thio-α-D-galactopyranoside, (11)

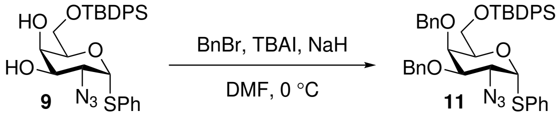

The diol **9** (2.54 g, 4.73 mmol) was dissolved in dry DMF (50 mL), cooled to 0 °C, added BnBr (2.80 mL, 23.6 mmol, 5 equiv.) and TBAI (0.349 g, 0.945 mmol, 20 mol%) before addition of NaH (939 mg, 23.5 mmol, 5 equiv.) and stirred at 0 °C for 2.5 h. The reaction mixture was then diluted with EtOAc, quenched with H_2O at 0 °C, washed with H_2O (x2), the water phase was backextracted with EtOAc and the combined organic phases were washed with more H_2O, dried over $MgSO_4$ and solvents evaporated *in vacuo*. The crude product was purified by flash column chromatography (SiO_2, CH_2Cl_2 : PE 2:1) to yield the pure title compound **11** (2.94 g, 4.11 mmol, 87%)(R_f 0.80 CH_2Cl_2) as a clear oil.

Characterisation

^{1}H NMR (500 MHz, $CDCl_3$) δ 7.64 (td, J = 7.9, 1.3 Hz, 4H), 7.50-7.32 (m, 15H), 7.26 (s, 4H ($CHCl_3$ overlap)), 7.23-7.15 (m, 3H), 5.55 (d, J = 5.4 Hz, 1H, H-1), 4.94 (d, J = 11.1 Hz, 1H, O4-Bn), 4.84 (d, J = 11.5 Hz, 1H, O3-Bn), 4.79 (d, J = 11.5 Hz, 1H, O3-Bn), 4.61 (d, J = 11.2 Hz, 1H, O4-Bn), 4.43 (dd, J = 10.6, 5.4 Hz, 1H, H-2), 4.34 (dd, J = 7.7, 5.7 Hz, 1H, H-5), 4.12-4.07 (m, 1H, H-4), 3.84 (dd, J = 10.1, 7.7 Hz, 1H, H-6a), 3.80 (dd, J = 10.6, 2.7 Hz, 1H, H-3), 3.67 (dd, J = 10.1, 5.8 Hz, 1H, H-6b), 1.07 (s, 9H).

^{13}C NMR (125 MHz, $CDCl_3$) δ 138.4, 137.6, 135.6, 133.4, 133.3, 133.2, 132.8, 130.0, 129.9, 129.1, 128.7, 128.4, 128.2, 128.1, 128.0, 127.91, 127.90, 127.7 (30 C-Ar), 88.0 (C-1), 79.3 (C-3), 75.2 (O4-Bn), 73.7 (C-4), 72.7 (O3-Bn), 72.0 (C-5), 62.2 (C-6), 60.6 (C-2), 27.0 (*t*Bu-Me), 19.3 (*t*Bu-C).

HRMS (ESI) m/z: $[M + H]^+$ Calcd for $C_{42}H_{46}N_3O_4SSi^+$ 716.2973; Found 716.2980.

$[\alpha]_D^{25}$ +138.2 (c 0.99, $CHCl_3$)

Spectra can be seen on page S27 - S31.

S1.5 2-deoxy-2-azido-3,4-di-*O*-benzyl-6-*O*-*tert*-butyldiphenylsilyl-α/β-D-galactopyranose, (13)

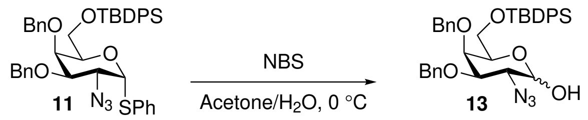

The thiophenol protected glycoside **11** (2.49 g, 3.48 mmol) was dissolved in acetone (150 mL) and H_2O (15 mL), cooled to 0 °C before adding NBS (2.47 g, 13.9 mmol, 4 equiv.). After 15 min the reaction mixture was diluted with Et_2O, washed with brine followed by 10% aq. $Na_2S_2O_3$ and finally brine. The organic phase was dried over $MgSO_4$ and solvents evaporated *in vacuo*. The crude product was purified by flash column chromatography (SiO_2, short, CH_2Cl_2) to yield the pure title compound **13** (2.06 g, 3.30 mmol, 95%, α:β 1:0.6)(R_f 0.18 CH_2Cl_2) as a clear oil.

Characterisation

Spectral data were in accordance with literature.[3]

S1.6 *O*-(2-deoxy-2-azido-3,4-di-*O*-benzyl-6-*O*-*tert*-butyldiphenylsilyl-α/β-D-galactopyranosyl) Trichloroacetimidate, (15)

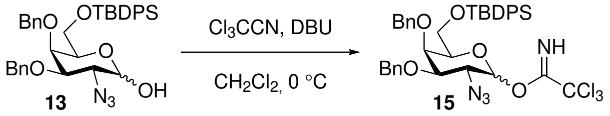

The free 1-OH **13** (1.72 g, 2.76 mmol) was dissolved in dry CH_2Cl_2 (100 mL), cooled to 0 °C before adding Cl_3CCN (1.40 mL, 14.0 mmol, 5 equiv.) followed by DBU (2 drops, cat.). After 45 min the reaction mixture was evaporated *in vacuo*. The crude product was purified by flash column chromatography (SiO_2, short, CH_2Cl_2 0.5% Et_3N) to yield the pure title compound **15** (2.00 g, 2.60 mmol, 94%, α:β 1:0.6)(R_f 0.80 CH_2Cl_2) as a clear oil.

Characterisation

Compound was wrongly reported in literature twice and spectral data were therefore not in accordance.[4,5]
^{1}H NMR (500 MHz, $CDCl_3$) δ 8.63 (s, 0.6H), 8.62 (s, 1H), 7.62-7.56 (m, 7H), 7.46-7.37 (m, 14H), 7.37-7.30 (m, 10H), 7.29-7.26 (m, 7H), 6.36 (d, J = 3.5 Hz, 1H. H-1α), 5.56 (d, J = 8.4 Hz, 0.6H, H-1β), 4.97-4.91 (m, 1.6H, OBn), 4.83 (d, J = 11.5 Hz, 1H, αOBn), 4.80 (d, J = 11.6 Hz, 1H, αOBn), 4.79-4.76 (m, 1.2H, βOBn), 4.65 (d, J = 11.1 Hz, 1H, αOBn), 4.60 (d, J = 11.2 Hz, 0.6H, βOBn), 4.20-4.14 (m, 2H, H-2α H-4α), 4.09-3.99 (m, 2.6H, H-3α H-5α H-2β), 3.96 (d, J = 2.2 Hz, 0.6H, H-4β), 3.88-3.79 (m, 2.2H, H-6aα H-6β), 3.73 (dd, J = 10.1, 5.8 Hz, 1H, H-6bα), 3.59-3.55 (m, 0.6H, H-5β), 3.46 (dd, J = 10.3, 2.8 Hz, 0.6H, H-3β), 1.05 (s, 5.4H), 1.04 (s, 9H).

^{13}C NMR (125 MHz, $CDCl_3$) δ 161.4 (C=NHβ), 160.8(C=NHα), 138.41, 138.35, 137.6, 137.4, 135.73, 135.67, 135.6, 133.3, 133.2, 133.21, 133.18, 129.99, 129.98, 129.94, 128.7, 128.5, 128.4, 128.3, 128.2, 128.1, 128.0, 127.91, 127.88, 127.84, 127.76, 97.3 (C-1β), 95.5 (C-1α), 91.2 ($CCl_3\alpha$), 90.8 ($CCl_3\beta$), 80.8 (C-3β), 77.4 (C-3α), 76.0(C-5β), 75.2(αOBn), 75.1(βOBn), 73.8 (C-5α), 73.1 (C-4α), 73.0 (C-4β), 72.50 (βOBn), 72.48 (αOBn), 62.9 (C-2β), 62.02 (C-6α), 62.00 (C-6β), 59.4 (C-2α), 27.0 (tBu-Me), 19.3 (tBu-C).

HRMS (MALDI) m/z: $[M + Na]^+$ Calcd for $C_{38}H_{41}Cl_3N_4O_5SiNa^+$ 789.1804; Found 789.1806.

Spectra can be seen on page S32 - S35.

S1.7 Phenyl 2-deoxy-2-azido-6-*O*-benzyl-thio-β-D-galactopyranoside, (10)

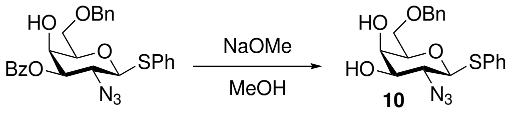

The benzoyl ester(Phenyl 2-deoxy-2-azido-4-*O*-benzoyl-6-*O*-benzyl-thio-β-D-galactopyranoside)[1] (0.868 g, 1.76 mmol) was dissolved in dry MeOH (50 mL) and added NaOMe (0.05 mL, 25% in MeOH, 0.22 mmol, 12 mol%). After 15 min the reaction mixture was neutralized using Amberlite resin (H^+) followed by filtration and evaporation *in vacuo*. The crude product was purified by flash column chromatography (SiO_2, short, 1% MeOH in CH_2Cl_2) to yield the pure title compound **10** (0.616 g, 1.59 mmol, 90%)(R_f 0.16 1% MeOH in CH_2Cl_2).

Characterisation

Spectral data were in accordance with literature.[6]

S1.8 Phenyl (2-deoxy-2-azido-3,4,6-tri-*O*-benzyl-thio-α/β-D-galactopyranoside, (12α/β)

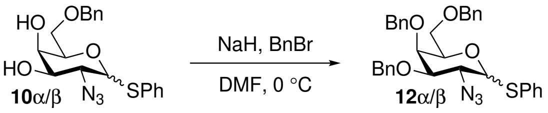

The diol **10**α/β (1.46 g, 3.77 mmol, α:β 1:0.2) was dissolved in dry DMF (50 mL), cooled to 0 °C before addition of NaH (597 mg, 14.9 mmol, 4 equiv.) and stirred for 25 min before addition of BnBr (1.30 mL, 11.0 mmol, 3 equiv.). The reaction mixture was stirred for 2 h at 0 °C then diluted with EtOAc, quenched with H_2O at 0 °C, washed with H_2O, the water phase was backextracted with EtOAc and the combined organic phases were washed with more H_2O (x2), dried over $MgSO_4$ and solvents evaporated *in vacuo*. The crude product was purified by flash column chromatography (SiO_2, CH_2Cl_2 : PE 1:1 $\rightarrow$ CH_2Cl_2) to yield the pure title compound **12**α/β (1.59 g, 2.79 mmol, 74%, α:β 1:0.2)(R_f 0.80 CH_2Cl_2).

Characterisation

Reported in literature, but not characterized.[7,8]
^{1}H NMR (500 MHz, $CDCl_3$) δ 7.62-7.58 (m, 0.4H), 7.56-7.50 (m, 2H), 7.48-7.27 (m, 19H), 7.26-7.21 (m, 3H), 5.64 (d, *J* = 5.4 Hz, 1H, H-1α), 4.93 (d, *J* = 11.3 Hz, 1H, αOBn), 4.90 (d, *J* = 11.5 Hz, 0.2H, βOBn), 4.78 (s, 2H, αOBn), 4.74 (d, *J* = 11.6 Hz, 0.2H, βOBn), 4.69 (d, *J* = 11.6 Hz, 0.2H, β), 4.58 (d, *J* = 11.2 Hz, 1H, αOBn), 4.55-4.48 (m, 1.4H, H-5α βOBn), 4.48-4.40 (m, 3.4H, H-2α αOBn H-1β βOBn), 4.09-4.05 (m, 1H, H-4α), 3.99-3.94 (m, 0.2H, H-4β), 3.87 (t, *J* = 9.9 Hz, 0.2H, H-2β), 3.82 (dd, *J* = 10.6, 2.7 Hz, 1H, H-3α), 3.69-3.63 (m, 1.4H, H-6aα H-6β), 3.63-3.59 (m, 0.2H, H-5β), 3.57 (dd, *J* = 9.4, 6.0 Hz, 1H, H-6bα), 3.44 (dd, *J* = 9.7, 2.7 Hz, 0.2H, H-3β).

^{13}C NMR (125 MHz, $CDCl_3$) δ 138.5, 138.3, 138.0, 137.9, 137.52, 137.49, 133.6, 132.9, 132.4, 132.0, 129.1, 128.9, 128.7, 128.62, 128.55, 128.51, 128.4, 128.3, 128.12, 128.09, 128.01, 127.95, 127.86, 127.84, 127.75, 127.65, 127.60 (48 C-Ar), 87.8 (C-1α), 86.5 (C-1β), 82.6 (C-3β), 79.3 (C-3α), 77.5 (C-5α), 75.0 (αOBn), 74.5 (βOBn), 73.7 (βOBn), 73.55 (H-4α), 73.55 (αOBn), 72.6 (αOBn), 72.5 (βOBn), 72.2 (C-4β), 70.6 (C-5α), 68.7 (C-6α), 68.6 (C-6β), 61.6 (C-2β), 60.5 (C-2α).

HRMS (MALDI) *m/z*: $[M + H]^+$ Calcd for $C_{33}H_{33}N_3O_4SNa^+$ 590.2084; Found 590.2092.

Spectra can be seen on page S36 - S39.

S1.9 2-deoxy-2-azido-3,4,6-tri-*O*-benzyl-α/β-D-galactopyranose, (14)

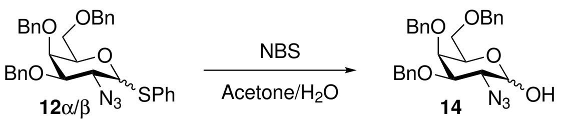

The thiophenol protected **12α/β** (1.51 g, 2.66 mmol) was dissolved in acetone (150 mL) and H_2O (15 mL), cooled to 0 °C before adding NBS (1.91 g, 10.7 mmol, 4 equiv.). After 15 min the reaction mixture was diluted with Et_2O, washed with brine followed by 10% aq. $Na_2S_2O_3$ and finally brine. The organic phase was dried over $MgSO_4$ and solvents evaporated *in vacuo*. The crude product was purified by flash column chromatography (SiO_2, short, CH_2Cl_2) to yield the pure title compound **14** (1.15 g, 2.41 mmol, 91%, α:β 2:1)(R_f 0.09 CH_2Cl_2) as a clear oil.

Characterisation

Spectral data were in accordance with literature.[9]

S1.10 *O*-(2-deoxy-2-azido-3,4, 6-tri-*O*-benzyl-α/β-D-galactopyranosyl) Trichloroacetimidate, (16)

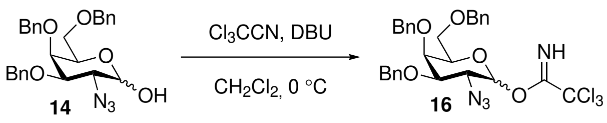

The free 1-OH **14** (1.15 g, 2.42 mmol) was dissolved in dry CH_2Cl_2 (100 mL), cooled to 0 °C before adding Cl_3CCN (1.20 mL, 12.0 mmol, 5 equiv.) followed by DBU (3 drops, cat.). After

1 h the reaction mixture was evaporated *in vacuo*. The crude product was purified by flash column chromatography (SiO_2, short, CH_2Cl_2 0.5% Et_3N) to yield the pure title compound **16** (1.24 g, 2.00 mmol, 83%, α:β 1:0.9)(R_f 0.38 CH_2Cl_2) as a clear oil.

Characterisation

Spectral data were in accordance with literature.[10,11]

S1.11 Phenyl (2-deoxy-2-azido-3,4-di-*O*-benzyl-6-*O*-*tert*-butyldiphenylsilyl-α-D-galactopyranosyl)(1→3)-2-deoxy-2-azido-6-*O*-*tert*-butyldiphenylsilyl-thio-α-D-galactopyranoside, (17)

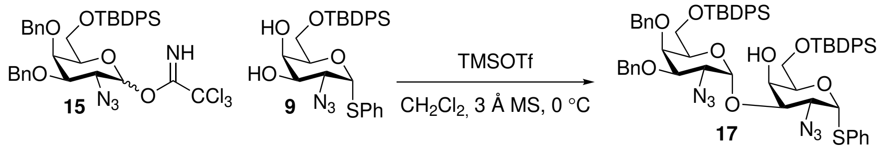

Donor (**15**) (2.00 g, 2.60 mmol, α:β 1:0.3) and acceptor (**9**) (2.33 g, 4.35 mmol, 1.7 equiv.) were dissolved in dry CH_2Cl_2 (50 mL) and stirred with 3 Å molecular sieves for 2 h. The reaction mixture was then cooled to 0 °C before adding TMSOTf (23 µL, 0.13 mmol, 5 mol%). The reaction was quenched after 15 min using Et_3N (0.1 mL), filtrated through a pad of celite and solvents were evaporated *in vacuo*. The crude product was purified by flash column chromatography (SiO_2, CH_2Cl_2 : PE 4:1) to yield the pure title compound **17** (1.91 g, 1.67 mmol, 64%)(R_f 0.87 CH_2Cl_2) as a clear oil along with the 4-*O* glycosylated product (0.26 g, 0.23 mmol, 9%) and recovered acceptor.

Characterisation

^{1}H NMR (500 MHz, $CDCl_3$) δ 7.69-7.64 (m, 8H), 7.50-7.33 (m, 22H), 7.26-7.21 (m, 8H), 5.65 (d, J = 5.5 Hz, 1H, H-1), 5.02 (d, J = 3.6 Hz, 1H, H-1'), 4.90 (d, J = 11.0 Hz, 1H, O4'-Bn), 4.79 (s, 2H, O3'-Bn), 4.56 (d, J = 11.0 Hz, 1H, O4'-Bn), 4.37 (t, J = 5.9 Hz, 1H, H-5), 4.31 (dd, J = 10.5, 5.5 Hz, 1H, H-2), 4.18 (t, J = 7.0 Hz, 1H, H-5'), 4.16-4.12 (m, 3H, H-4 H-2' H-4'), 4.08 (dd, J = 10.5, 2.5 Hz, 1H, H-3'), 3.96 (dd, J = 10.6, 5.8 Hz, 1H, H-6a), 3.90-3.85 (m, 2H, H-3 H-6b), 3.85 – 3.80 (m, 3H, H-6'), 3.34 (br s, 1H, OH-4), 1.09 (s, 9H), 1.05 (s, 9H).

^{13}C NMR (125 MHz, $CDCl_3$) δ 138.5, 137.6, 135.8, 135.77, 135.67, 135.65, 133.9, 133.5, 133.4, 133.2, 133.1, 132.1, 129.94, 129.89, 129.2, 128.7, 128.4, 128.23, 128.21, 128.0, 127.92, 127.87, 127.87, 127.7, 127.6 (42 C-Ar), 94.0 (C-1'), 87.6 (C-1), 78.6 (C-3'), 75.2 (O4'-Bn), 75.0 (C-3), 73.6 (C-4'), 72.7 (O3'-Bn), 72.3 (C-5'), 71.2 (C-5), 64.9 (C-4), 63.3 (C-6), 62.1 (C-6'), 60.4 (C-2'), 59.6 (C-2), 27.1 (tBu-Me), 27.0 (tBu-Me), 19.36 (tBu-C), 19.35 (tBu-C).

HRMS (ESI) m/z: $[M + Na]^+$ Calcd for $C_{64}H_{72}N_6O_8SSi_2Na^+$ 1163.4563; Found 1163.4589.

$[\alpha]_D^{25}$ +113.9 (c 1.67, $CHCl_3$)

Spectra can be seen on page S40 - S44.

S1.12 Phenyl (2-deoxy-2-azido-3,4,6-tri-*O*-benzyl-α-D-galactopyranosyl)(1→3)-2-deoxy-2-azido-6-*O*-*tert*-butyldiphenylsilyl-thio-α-D-galactopyranoside, (19)

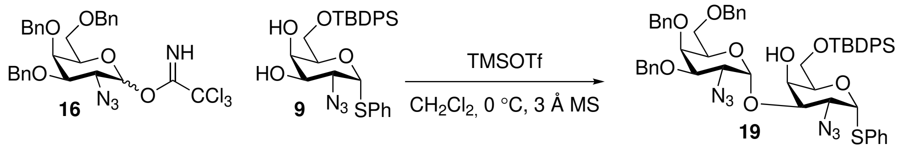

Donor (**16**) (625 mg, 1.01 mmol, α:β 1:0.9) and acceptor (**9**) (649 mg, 1.21 mmol, 1.2 equiv.) were dissolved in dry CH_2Cl_2 (20 mL) and stirred with 3 Å molecular sieves for 1 h. The reaction mixture was then cooled to 0 °C before adding TMSOTf (9 µL, 0.05 µmol, 5 mol%). The reaction was quenched after 15 min using Et_3N (0.1 mL), filtrated through a pad of celite and solvents were evaporated *in vacuo*. The crude product was purified by flash column chromatography (SiO_2, CH_2Cl_2 : toluene 9:1) to yield most of the pure title compound **19** (361 mg, 364 µmol, 36%)(R_f 0.50 CH_2Cl_2) as a clear oil along with mixed fractions of 3-*O*/4-*O* glycosylated products (179 mg, 180 µmol, 18%, 3-*O*:4-*O* 1:0.4, calcd total 3-*O* yield 49%). The 3-*O*/4-*O* mixture was found easier separated after the ensuing benzylation.

Characterisation

^{1}H NMR (500 MHz, $CDCl_3$) δ 7.73-7.67 (m, 4H), 7.53-7.48 (m, 2H), 7.47-7.41 (m, 5H), 7.41-7.36 (m, 12H), 7.35-7.29 (m, 7H), 5.65 (d, J = 5.5 Hz, 1H, H-1), 5.09 (d, J = 3.7 Hz, 1H, H-1'), 4.90 (d, J = 11.2 Hz, 1H, O4'-Bn), 4.78 (d, J = 11.4 Hz, 1H, O3'-Bn), 4.72 (d, J = 11.4 Hz, 1H, O3'-Bn), 4.60 (d, J = 11.7 Hz, 1H, O6'-Bn), 4.57 (d, J = 11.1 Hz, 1H, O4'-Bn), 4.52 (d, J = 11.7 Hz, 1H, O6'-Bn), 4.43 (t, J = 5.9 Hz, 1H, H-5), 4.32 (dd, J = 10.5, 5.4 Hz, 1H, H-2), 4.26 (t, J = 6.8 Hz, 1H, H-5'), 4.21-4.19 (m, 1H, H-4), 4.17 (dd, J = 10.6, 3.7 Hz, 1H, H-2'), 4.12 (br s, 1H, H-4'), 4.05 (dd, J = 10.5, 2.6 Hz, 1H, H-3'), 4.00 (dd, J = 10.6, 5.7 Hz, 1H, H-6a), 3.94 (dd, J = 10.5, 2.9 Hz, 1H, H-3), 3.91 (dd, J = 10.7, 6.0 Hz, 1H, H-6b), 3.73-3.64 (m, 2H, H-6'), 3.35 (s, 1H, OH-4), 1.08 (s, 9H).

^{13}C NMR (125 MHz, $CDCl_3$) δ 138.3, 138.0, 137.4, 135.8, 135.7, 133.7, 133.3, 133.2, 132.2, 129.9, 129.1, 128.6, 128.5, 128.4, 128.2, 128.1, 128.02, 127.96, 127.86, 127.84, 127.7 (36 C-Ar), 94.0 (C-1'), 87.8 (C-1), 78.3 (C-3'), 75.10 (C-3), 75.05 (O4'-Bn), 73.5 (O6'-Bn), 73.2 (C-4'), 72.3 (O3'-Bn), 71.3 (C-5), 70.5 (C-5'), 68.4 (C-6'), 65.0 (C-4), 63.3 (C-6), 60.3 (C-2'), 59.6 (C-2), 27.0 (tBu-Me), 19.3 (tBu-C).

HRMS (ESI) m/z: $[M + Na]^+$ Calcd for $C_{55}H_{60}N_6O_8SSiNa^+$ 1015.3855; Found 1015.3820.

$[\alpha]_D^{25}$ +116.0 (c 1.01, $CHCl_3$)

Spectra can be seen on page S45 - S49.

S1.13 Phenyl (2-deoxy-2-azido-3,4-di-*O*-benzyl-6-*O*-*tert*-butyldiphenylsilyl-α-D-galactopyranosyl)(1→3)-2-deoxy-2-azido-6-*O*-benzyl-thio-β-D-galactopyranoside, (20)

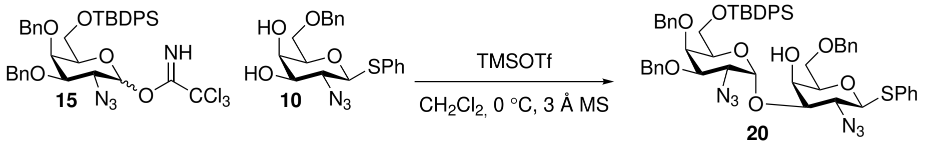

A solution of donor (**15**) (700 mg, 0.911 mmol, α:β 1:0.3, 1.2 equiv.) in dry CH_2Cl_2 (7.5 mL) was mixed with a solution of acceptor (**10**) (297 mg, 0.767 mmol) in dry CH_2Cl_2 (7.5 mL) and stirred with 3 Å molecular sieves for 1.5 h. The reaction mixture was then cooled to 0 °C before adding TMSOTf (7 µL, 0.04 µmol, 5 mol%). The reaction was quenched after 15 min using Et_3N (0.1 mL), filtrated through a pad of celite and solvents were evaporated *in vacuo*. The crude product was purified by flash column chromatography (SiO_2, CH_2Cl_2) to yield the title compound **20** (455 mg, 458 µmol, 60%)(R_f 0.27 CH_2Cl_2) as a clear oil along with the 4-*O* glycosylated product as a mixture with Cl_3CCN (250 mg, 252 µmol, 33%).

Characterisation

^{1}H NMR (500 MHz, $CDCl_3$) δ 7.68-7.59 (m, 9H), 7.47-7.31 (m, 27H), 7.25-7.19 (m, 6H), 5.01 (d, J = 3.5 Hz, 1H, H-1'), 4.89 (d, J = 11.1 Hz, 1H, O4'-Bn), 4.80 (d, J = 11.4 Hz, 1H, O3'-Bn), 4.77 (d, J = 11.4 Hz, 1H, O3'-Bn), 4.59 (s, 2H, O6-Bn), 4.54 (d, J = 11.2 Hz, 1H, O4'-Bn), 4.38 (d, J = 10.2 Hz, 1H, H-1), 4.12 (t, J = 7.0 Hz, 1H, H-5'), 4.08 (dd, J = 10.3, 3.5 Hz, 1H, H-2'), 4.07-4.03 (m, 3H, H-4 H-3' H-4'), 3.82 (dd, J = 10.1, 5.7 Hz, 1H, H-6a), 3.79 (dd, J = 10.1, 5.8 Hz, 1H, H-6n), 3.76 (d, J = 6.9 Hz, 2H, H-6'), 3.71 (dd, J = 10.1, 9.7 Hz, 1H, H-2), 3.58 (dd, J = 9.7, 3.0 Hz, 1H, H-3), 3.54 (t, J = 5.7 Hz, 1H, H-5), 3.07 (s, 1H, 4-OH), 1.06 (s, 9H).

^{13}C NMR (125 MHz, $CDCl_3$) δ 138.3, 137.9, 137.5, 135.61, 135.59, 133.4, 133.14, 133.10, 132.0, 129.93, 129.89, 129.2, 128.7, 128.6, 128.4, 128.2, 128.1, 127.93, 127.88, 127.8 (36 C-Ar), 93.9 (C-1'), 86.6 (C-1), 78.4 (C-3'), 77.9 (C-3), 77.1 (C-5), 75.1 (O4'-Bn), 73.8 (O6-Bn), 73.5 (C-4'), 72.7 (O3'-Bn), 72.3 (C-5'), 69.4 (C-6), 64.3 (C-4), 62.4 (C-6'), 61.0 (C-2), 60.1 (C-2'), 27.0 (*t*Bu-Me), 19.3 (*t*Bu-C).

HRMS (ESI) m/z: $[M + Na]^+$ Calcd for $C_{55}H_{60}N_6O_8SSiNa^+$ 1015.3855; Found 1015.3869.

$[\alpha]_D^{25}$ +54.3 (c 1.01, $CHCl_3$)

Spectra can be seen on page S50 - S54.

S1.14 Phenyl (2-deoxy-2-azido-3,4,6-tri-*O*-benzyl-α-D-galactopyranosyl)(1→3)-2-deoxy-2-azido-4,6-di-*O*-benzyl-thio-β-D-galactopyranoside, (26)

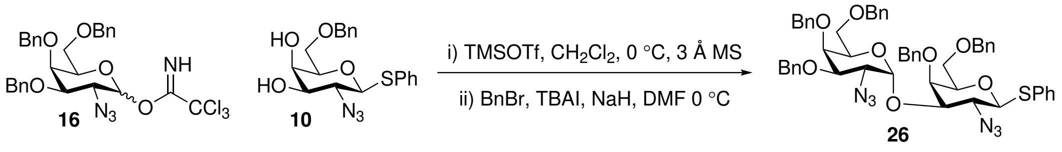

A solution of donor (**16**) (457 mg, 0.737 mmol, α:β 1:0.9, 1.0 equiv.) in dry CH_2Cl_2 (7.5 mL) was mixed with a solution of acceptor (**10**) (297 mg, 0.767 mmol, 1.0 equiv.) in dry CH_2Cl_2 (7.5 mL) and stirred with 3 Å molecular sieves for 1.5 h. The reaction mixture was then cooled to 0 °C before adding TMSOTf (6 µL, 0.03 µmol, 5 mol%). The reaction was quenched after 25 min using Et_3N (0.1 mL), filtrated through a pad of celite and solvents were evaporated *in vacuo*. The crude product was purified by flash column chromatography (SiO_2, 2% Acetone in toluene) to yield the 3-*O* glycosylated product **21** as a mixture with the 4-*O* isomer (299 mg, 354 µmol, 48%, 3*O*:4*O* 1.7:1)(R_f 0.22 2% Acetone in toluene) as a clear oil along. The mixed disaccharide was dissolved in dry DMF (10 mL) and cooled to 0 °C before adding BnBr (0.21 mL, 1.8 mmol, 5 equiv.) and TBAI (26 mg, 70 µmol, 20 mol%) followed by NaH (72 mg, 60%, 1.8 mmol, 5 equiv.). The reaction mixture was stirred for 2 h at 0 °C then diluted with EtOAc, quenched with H_2O at 0 °C, washed with H_2O (x2), the water phase was backextracted with EtOAc and the combined organic phases were dried over $MgSO_4$ and solvents evaporated *in vacuo*. The crude product was purified by flash column chromatography (SiO_2, CH_2Cl_2:toluene 1:1 → CH_2Cl_2) to yield the title compound **26** (206 mg, 220 µmol, 62%)(R_f 0.08 CH_2Cl_2:toluene 1:1) as a clear oil along with the 4-*O* isomer (60 mg, 64 µmol, 18%).

Characterisation

^{1}H NMR (500 MHz, $CDCl_3$) δ 7.62-7.57 (m, 2H), 7.42-7.26 (m, 31H), 7.24-7.20 (m, 3H), 5.19 (br s, 1H, H-1'), 5.02 (d, J = 11.2 Hz, 1H, O4-Bn), 4.89 (d, J = 11.3 Hz, 1H, O4'-Bn), 4.72 (d, J = 11.3 Hz, 1H, O3'-Bn), 4.68 (d, J = 11.3 Hz, 1H, O3'-Bn), 4.56-4.44 (m, 6H, O4-Bn O4'-Bn O6-Bn O6'-Bn), 4.37 (d, J = 10.0 Hz, 1H, H-1), 4.19 (t, J = 6.5 Hz, 1H, H-5'), 4.08-4.03 (m, 3H, H-2' H-3' H-4'), 4.00 (d, J = 2.3 Hz, 1H, H-4), 3.89 (t, J = 10.0 Hz, 1H, H-2), 3.69-3.64 (m, 3H, H-3 H-6), 3.59 (d, J = 6.5 Hz, 2H, H-6'), 3.56 (t, J = 6.3 Hz, 1H, H-5).

^{13}C NMR (125 MHz, $CDCl_3$) δ 138.6, 138.4, 138.1, 137.8, 137.6, 132.8, 131.9, 129.0, 128.63, 128.60, 128.5, 128.4, 128.3, 128.2, 128.1, 128.0, 127.9, 127.8, 127.7, 127.62, 127.59 (36 C-Ar), 94.9 (C-1'), 86.7 (C-1), 78.0 (C-3), 77.7 (C-3'), 77.3 (C-5), 74.9 (O4'-Bn), 74.6 (O4-Bn), 73.7 (O6-Bn), 73.5 (C-4'), 73.4 (O6'-Bn), 72.5 (O3'-Bn), 71.1 (C-4), 70.1 (C-5'), 68.8 (C-6'), 68.3 (C-6), 61.3 (C-2), 59.8 (C-2').

HRMS (ESI) m/z: $[M + Na]^+$ Calcd for $C_{53}H_{54}N_6O_8SNa^+$ 957.3616; Found 957.3595.

$[\alpha]_D^{25}$ +58.7 (c 1.00, $CHCl_3$)

Spectra can be seen on page S55 - S59.

S1.15 Phenyl (2-deoxy-2-azido-3,4-di-*O*-benzyl-6-*O*-*tert*-butyldiphenylsilyl-α-D-galactopyranosyl)(1→3)-2-deoxy-2-azido-4-*O*-benzyl-6-*O*-*tert*-butyldiphenylsilyl-thio-α-D-galactopyranoside, (22)

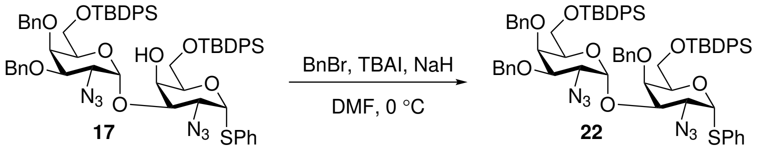

The 4-OH disaccharide **17** (1.91 g, 1.67 mmol was dissolved in dry DMF (150 mL) and cooled to 0 °C before adding BnBr (1.0 mL, 8.4 mmol, 5 equiv.) and TBAI (134 mg, 363 µmol, 22 mol%) followed by NaH (336 mg, 60%, 8.4 mmol, 5 equiv.). The reaction mixture was stirred for 2 h at 0 °C then diluted with EtOAc, quenched with H_2O at 0 °C, washed with H_2O (x3), the water phase was backextracted with EtOAc and the combined organic phases were dried over $MgSO_4$ and solvents evaporated *in vacuo.* The crude product was purified by flash column chromatography (SiO_2, CH_2Cl_2:PE 2:1) to yield the title compound **22** (1.88 g, 1.53 mmol, 91%)(R_f 0.48 CH_2Cl_2:PE 2:1).

Characterisation

^{1}H NMR (500 MHz, $CDCl_3$) δ 7.72-7.66 (m, 4H), 7.64-7.58 (m, 4H), 7.48-7.27 (m, 28H), 7.25-7.14 (m, 7H), 5.59 (d, J = 5.5 Hz, 1H, H-1), 5.29 (d, J = 3.5 Hz, 1H, H-1'), 5.07 (d, J = 10.8 Hz, 1H, O4-Bn), 4.94 (d, J = 10.9 Hz, 1H, O4'-Bn), 4.81 (d, J = 11.9 Hz, 1H, O3'-Bn), 4.78 (d, J = 11.9 Hz, 1H, O3'-Bn), 4.65 (d, J = 10.8 Hz, 1H, O4-Bn), 4.58 (d, J = 10.9 Hz, 1H, O4'-Bn), 4.49 (dd, J = 10.8, 5.5 Hz, 1H, H-2), 4.29 (dd, J = 8.0, 6.0 Hz, 1H, H-5'), 4.26-4.21 (m, 2H, H-5 H-3'), 4.19 (d, J = 1.4 Hz, 1H, H-4'), 4.18 (d, J = 2.0 Hz, 1H, H-4), 4.06 (dd, J = 10.7, 3.5 Hz, 1H, H-2'), 4.02 (dd, J = 10.8, 2.5 Hz, 1H, H-3), 3.89 (d, J = 6.9 Hz, 1H, H-6), 3.83 (dd, J = 9.9, 8.2 Hz, 1H, H-6a'), 3.62 (dd, J = 10.0, 5.7 Hz, 1H, H-6b'), 1.12 (s, 9H), 1.04 (s, 9H).

^{13}C NMR (125 MHz, $CDCl_3$) δ 138.5, 138.4, 137.7, 135.7, 135.64, 135.62, 133.5, 133.3, 133.11, 133.08, 133.05, 132.9, 130.0, 129.94, 129.90, 129.88, 129.1, 128.7, 128.44, 128.40, 128.3, 128.2, 128.1, 128.0, 127.93, 127.87, 127.76, 127.74, 127.70 (48 C-Ar), 94.6 (C-1'), 87.2 (C-1), 77.6 (C-3'), 75.3 (O4'-Bn), 75.1 (O4-Bn), 74.3 (C-3), 73.8 (C-4'), 72.7 (O3'-Bn), 71.7 (C-4 C-5 C-5'), 62.1 (C-6), 61.9 (C-6'), 60.4 (C-2), 59.8 (C-2'), 27.2 (*t*Bu-Me), 27.0 (*t*Bu-Me), 19.4 (*t*Bu-C), 19.3 (*t*Bu-C).

HRMS (ESI) m/z: $[M + Na]^+$ Calcd for $C_{71}H_{78}N_6O_8SSi_2Na^+$ 1253.5053; Found 1253.5042.

$[\alpha]_D^{25}$ +160.3 (c 0.79, $CHCl_3$)

Spectra can be seen on page S60 - S64.

S1.16 Phenyl (2-deoxy-2-azido-3,4-di-*O*-benzyl-6-*O*-*tert*-butyldiphenyl-silyl-α-D-galactopyranosyl)(1→3)-2-deoxy-2-azido-4-*O*-benzyl-6-*O*-benzyl-thio-α-D-galactopyranoside, (23)

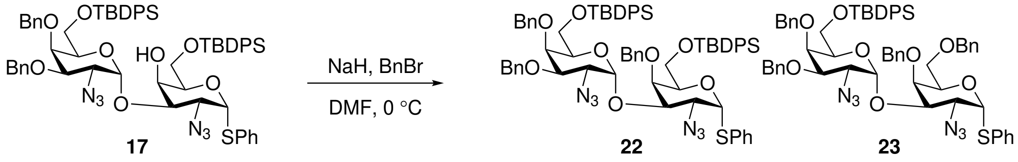

The 4-OH disaccharide **17** (57.1 mg, 50.0 µmol was dissolved in dry DMF (1 mL) and cooled to 0 °C before adding NaH (4.0 mg, 100 µmol, 60%, 2 equiv.). The reaction mixture was stirred for 5 min before addition of BnBr (9 µL, 76 µmol, 1.5 equiv.). The reaction mixture was stirred overnight, slowly reaching rt. The reaction mixture was diluted with EtOAc, quenched with H_2O at 0 °C, washed with H_2O (x3), the water phase was backextracted with EtOAc and the combined organic phases were dried over $MgSO_4$ and solvents evaporated *in vacuo*. Crude NMR suggested a 1:1 ratio of the two products **22** and **23**. The mixture was purified by flash column chromatography (SiO_2, CH_2Cl_2:PE 2:1) to yield the title compound **22** (20 mg, 16 µmol, 33%) along with the byproduct **23** (15 mg, 14 µmol, 28%)(R_f 0.55 CH_2Cl_2:PE 4:1 (same as the starting material **17**)).

Characterisation of byproduct 23

^{1}H NMR (500 MHz, $CDCl_3$) δ 7.70-7.64 (m, 4H), 7.48-7.44 (m, 3H), 7.44-7.38 (m, 7H), 7.38-7.28 (m, 17H), 7.26-7.20 (m, 11H), 5.67 (d, J = 5.5 Hz, 1H, H-1), 5.21 (d, J = 3.5 Hz, 1H, H-1'), 5.05 (d, J = 11.0 Hz, 1H, O4-Bn), 4.91 (d, J = 11.0 Hz, 1H, O4'-Bn), 4.78 (d, J = 1.4 Hz, 2H, O3'-Bn), 4.56 (d, J = 10.9 Hz, 2H, O4-Bn O4'-Bn), 4.51 (dd, J = 10.8, 5.5 Hz, 1H, H-2), 4.46-4.37 (m, 3H, O6-Bn H-5), 4.23-4.15 (m, 3H, H-5' H-3' H-4'), 4.06 (d, J = 1.9 Hz, 1H, H-4), 4.02 (dd, J = 10.6, 3.5 Hz, 1H, H-2'), 3.98 (dd, J = 10.8, 2.5 Hz, 1H, H-3), 3.87-3.82 (m, 2H, H-6'), 3.62 (dd, J = 9.3, 7.4 Hz, 1H, H-6a), 3.50 (dd, J = 9.3, 6.0 Hz, 1H, H-6b), 1.10 (s, 9H).

^{13}C NMR (125 MHz, $CDCl_3$) δ 138.5, 138.4, 137.9, 137.7, 135.7, 133.7, 133.5, 133.1, 132.4, 129.9, 129.1, 128.7, 128.6, 128.5, 128.4, 128.3, 128.2, 128.1, 128.0, 128.0, 127.9, 127.9, 127.8, 127.7, 127.6 (42 C-Ar), 94.5 (C-1'), 87.1 (C-1), 77.6 (C-3'), 75.3 (O4'-Bn), 74.9 (O4-Bn), 74.1 (C-3), 73.8 (C-4'), 73.5 (O6-Bn), 72.7 (O3'-Bn), 72.0 (C-4), 71.7 (C-5'), 70.4 (C-5), 68.4 (C-6), 62.1 (C-6'), 60.3 (C-2), 59.8 (C-2'), 27.1 (*t*Bu-Me), 19.4 (*t*Bu-C).

HRMS (ESI) m/z: $[M + Na]^+$ Calcd for $C_{62}H_{66}N_6O_8SSiNa^+$ 1105.4324; Found 1105.4332.

$[\alpha]_D^{25}$ +117.9 (c 0.76, $CHCl_3$)

Spectra can be seen on page S65 - S69.

S1.17 Phenyl (2-deoxy-2-azido-3,4,6-tri-*O*-benzyl-α-D-galactopyranosyl)(1→3)-2-deoxy-2-azido-4-*O*-benzyl-6-*O*-*tert*-butyldiphenylsilyl-thio-α-D-galactopyranoside, (24)

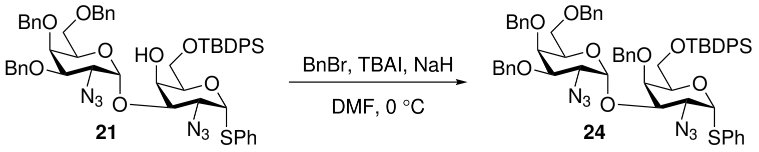

The 4-OH disaccharide **21** (341 mg, 344 µmol was dissolved in dry DMF (10 mL) and cooled to 0 °C before adding BnBr (0.20 mL, 1.7 mmol, 5 equiv.) and TBAI (25 mg, 68 µmol, 20 mol%) followed by NaH (70 mg, 60%, 1.8 mmol, 5 equiv.). The reaction mixture was stirred for 2 h at 0 °C then diluted with EtOAc, quenched with H_2O at 0 °C, washed with H_2O (x2), the water phase was backextracted with EtOAc and the combined organic phases were dried over $MgSO_4$ and solvents evaporated *in vacuo.* The crude product was purified by flash column chromatography (SiO_2, CH_2Cl_2:toluene 1:1) to yield the title compound **24** (319 mg, 294 µmol, 86%)(R_f 0.35 CH_2Cl_2:toluene 1:1).

Characterisation

^{1}H NMR (500 MHz, $CDCl_3$) δ 7.66-7.58 (m, 5H), 7.44-7.27 (m, 33H), 7.23-7.13 (m, 2H), 5.57 (d, J = 5.4 Hz, 1H, H-1), 5.33 (d, J = 3.5 Hz, 1H, H-1'), 5.06 (d, J = 10.8 Hz, 1H, 4O-Bn), 4.92 (d, J = 11.2 Hz, 1H, 4O'-Bn), 4.76 (d, J = 11.3 Hz, 1H, 6O'-Bn), 4.71 (d, J = 11.3 Hz, 1H, 6O'-Bn), 4.64 (d, J = 10.8 Hz, 1H, 4O-Bn), 4.61 (d, J = 12.0 Hz, 1H, O3'-Bn), 4.59-4.54 (m, 2H, 3O'-Bn 4O'-Bn), 4.49 (dd, J = 10.8, 5.4 Hz, 1H, H-2), 4.34 (t, J = 6.8 Hz, 1H, H-5), 4.26 (t, J = 6.5 Hz, 1H, H-5'), 4.19 (br s, 1H, H-3), 4.17 (dd, J = 10.7, 2.5 Hz, 1H, H-3'), 4.11 (br s, 1H, H-4'), 4.08 (dd, J = 10.7, 3.2 Hz, 2H, H-2' H-3), 3.83 (dd, J = 10.0, 7.9 Hz, 1H, H-6a), 3.72-3.60 (m, 3H, H-6b H-6'), 1.06 (s, 9H).

^{13}C NMR (125 MHz, $CDCl_3$) δ 138.4, 138.3, 138.1, 137.6, 135.7, 135.6, 133.24, 133.17, 133.1, 133.0, 129.99, 129.95, 129.1, 128.7, 128.55, 128.46, 128.44, 128.3, 128.2, 128.1, 127.94, 127.91, 127.89, 127.81, 127.76 (42 C-Ar), 94.6 (C-1), 87.5 (C-1'), 77.6 (C-3'), 75.1 (O4-Bn), 75.0 (O4'-Bn), 74.4 (C-3), 73.6 (C-4'), 73.5 (O3'-Bn), 72.5 (O6'-Bn), 71.9 (C-5), 71.8 (C-4), 70.2 (C-5'), 68.7 (C-6'), 62.1 (C-6), 60.4 (C-2), 59.7 (C-2'), 27.0 (tBu-Me), 19.3 (tBu-C).

HRMS (MALDI) m/z: $[M + Na]^+$ Calcd for $C_{62}H_{66}N_6O_8SSiNa^+$ 1105.4324; Found 1105.4365.

$[\alpha]_D^{25}$ +158.8 (c 1.22, $CHCl_3$)

Spectra can be seen on page S70 - S74.

S1.18 Phenyl (2-deoxy-2-azido-3,4-di-*O*-benzyl-6-*O*-*tert*-butyldiphenyl-silyl-α-D-galactopyranosyl)(1→3)-2-deoxy-2-azido-4,6-di-*O*-benzyl-thio-β-D-galactopyranoside, (25)

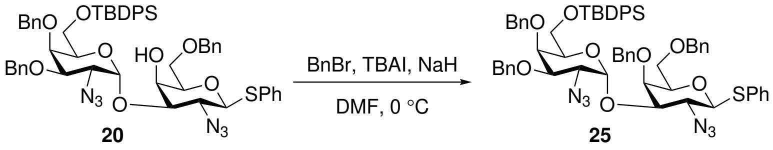

The 4-OH disaccharide **20** (432 mg, 435 µmol was dissolved in dry DMF (10 mL) and cooled to 0 °C before adding BnBr (0.26 mL, 2.2 mmol, 5 equiv.) and TBAI (32 mg, 87 µmol, 20 mol%) followed by NaH (88 mg, 60%, 2.2 mmol, 5 equiv.). The reaction mixture was stirred for 2 h at 0 °C then diluted with EtOAc, quenched with H_2O at 0 °C, washed with H_2O (x2), the water phase was backextracted with EtOAc and the combined organic phases were dried over $MgSO_4$ and solvents evaporated *in vacuo.* The crude product was purified by flash column chromatography (SiO_2, CH_2Cl_2:toluene 1:1) to yield the title compound **25** (292 mg, 269 µmol, 62%)(R_f 0.26 CH_2Cl_2:toluene 1:1).

Characterisation

^{1}H NMR (500 MHz, $CDCl_3$) δ 7.66-7.58 (m, 7H), 7.44-7.26 (m, 30H), 7.25-7.21 (m, 8H), 5.17 (d, J = 3.5 Hz, 1H, H-1'), 5.02 (d, J = 11.1 Hz, 1H, O4-Bn), 4.89 (d, J = 11.3 Hz, 1H, O4'-Bn), 4.76 (d, J = 11.4 Hz, 1H, O3'-Bn), 4.73 (d, J = 11.4 Hz, 1H, O3'-Bn), 4.57-4.51 (m, 2H, O4-Bn O4'-Bn), 4.48 (d, J = 11.8 Hz, 1H, O6-Bn), 4.43 (d, J = 11.8 Hz, 1H, O6-Bn), 4.33 (d, J = 10.0 Hz, 1H, H-1), 4.14-4.07 (m, 2H, H-3' H-5'), 4.04-3.99 (m, 2H, H-2' H-4'), 3.98 (d, J = 2.0 Hz, 1H, H-4), 3.90 (t, J = 10.0 Hz, 1H, H-2), 3.77-3.70 (m, 2H, H-6'), 3.68-3.61 (m, 3H, H-3 H-6), 3.51 (t, J = 6.6 Hz, 1H, H-5), 1.04 (s, 9H).

^{13}C NMR (125 MHz, $CDCl_3$) δ 138.5, 138.4, 137.8, 137.6, 135.7, 135.6, 133.4, 133.1, 132.8, 132.1, 130.0, 129.9, 129.1, 128.7, 128.6, 128.41, 128.36, 128.29, 128.19, 128.13, 128.04, 128.01, 127.9, 127.8, 127.7, 127.6 (42 C-Ar), 94.1 (C-1'), 86.9 (C-1), 77.8 (C-3'), 77.3 (C-5), 77.2 (C-3), 75.1 (O4'-Bn), 74.5 (O4-Bn), 73.7 (C-4'), 73.6 (O6-Bn), 72.8 (O3'-Bn), 72.0 (C-5'), 70.5 (C-4), 68.3 (C-6), 62.7 (C-6'), 61.3 (C-2), 59.8 (C-2'), 27.0 (*t*Bu-Me), 19.4 (*t*Bu-C).

HRMS (MALDI) m/z: $[M + Na]^+$ Calcd for $C_{62}H_{66}N_6O_8SSiNa^+$ 1105.4324; Found 1105.4306.

$[\alpha]_D^{25}$ +58.0 (c 1.45, $CHCl_3$)

Spectra can be seen on page S75 - S79.

S1.19 2-deoxy-2-azido-3,4-di-*O*-benzyl-6-*O*-*tert*-butyldiphenylsilyl-α-D-galactopyranosyl)(1→3)-2-deoxy-2-azido-4-*O*-benzyl-6-*O*-*tert*-butyldiphenylsilyl-α/β-D-galactopyranose, (27)

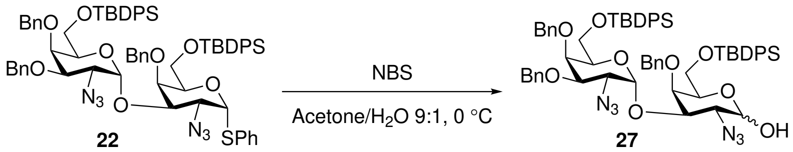

Thiophenoldisaccharide (**22**) (1.88 g, 1.53 mmol was dissolved in acetone (90 mL) and H_2O (10 mL), cooled to 0 °C before adding NBS (1.10 g, 6.18 mmol, 4 equiv.). The reaction mixture was diluted after 15min with Et_2O, and washed with 10% aq. $Na_2S_2O_3$, the water phase was backextracted with Et_2O and the combined organic phases were washed with brine, dried over $MgSO_4$ and solvents evaporated *in vacuo*. The crude product was purified by flash column chromatography (SiO_2, CH_2Cl_2) to yield the title compound **27** (1.28 g, 1.12 mmol, 74%, α:β 1:1)(R_f 0.53 CH_2Cl_2).

Characterisation

^{1}H NMR (500 MHz, $CDCl_3$) δ 7.71-7.57 (m, 17H), 7.49-7.27 (m, 49H), 7.25-7.22 (m, 9H), 5.28 (d, J = 3.5 Hz, 2H, H-1 H-1), 5.26 (d, J = 3.6 Hz, 1H, H-1), 5.09 (d, J = 10.8 Hz, 1H, O4-Bn), 5.06 (d, J = 10.8 Hz, 1H, O4-Bn), 4.94 (d, J = 11.1 Hz, 1H, O4'-Bn), 4.90 (d, J = 11.2 Hz, 1H, O4'-Bn), 4.79 (d, J = 11.4 Hz, 1H, O3'-Bn), 4.79-4.76 (m, 2H, O3'-Bn), 4.76 (d, J = 11.4 Hz, 1H, O'3-Bn), 4.65 (d, J = 10.8 Hz, 1H, O4-Bn), 4.62 (d, J = 10.8 Hz, 1H, O4-Bn), 4.59 (d, J = 11.1 Hz, 1H, O4'-Bn), 4.56 (d, J = 11.3 Hz, 1H, O4'-Bn), 4.38-4.33 (m, 1H, H-1β), 4.234.12 (m, 7H), 4.09-3.97 (m, 6H, H-2 H-2' H-2'), 3.89-3.69 (m, 9H, H-6 H-6' H-6 H-6' H-3), 3.68 (dd, J = 10.6, 2.6 Hz, 1H, H-3), 3.40 (dd, J = 7.9, 7.8 Hz, 1H, H-3β), 2.96 (d, J = 6.4 Hz, 1H, OH-1β), 2.44 (d, J = 1.6 Hz, 1H, OH-1α), 1.09 (s, 9H), 1.08 (s, 9H), 1.06 (s, 9H), 1.05 (s, 9H).

^{13}C NMR (125 MHz, $CDCl_3$) δ 138.6, 138.5, 138.34, 138.31, 137.7, 137.6, 135.73, 135.69, 135.67, 135.66, 135.62, 133.52, 133.45, 133.2, 133.25, 133.21, 133.19, 133.17, 133.01, 129.98, 129.96, 129.94, 129.89, 129.87, 128.72, 128.69, 128.42, 128.41, 128.39, 128.30, 128.25, 128.22, 128.18, 128.17, 128.07, 127.93, 127.92, 127.91, 127.89, 127.81, 127.73, 127.72, 127.66 (84 C-Ar), 96.6 (C-1β), 95.2 (C-1), 94.3 (C-1), 92.3 (C-1), 77.7 (C-3), 75.5 (C-3), 75.21 (C-3β), 75.18 (O4-Bn), 75.02 (O4-Bn), 74.99 (O4-Bn), 74.94 (O4-Bn), 73.8, 73.7, 73.6, 72.74 (O3'-Bn), 72.73 (O3'-Bn), 72.2, 72.0, 71.9, 71.2, 70.3, 64.4 (C-2β), 62.9 (C-6), 62.3 (C-6), 62.0 (C-6), 61.9 (C-6), 60.8 (C-2), 59.9 (C-2), 59.8 (C-2), 27.05 (tBu-Me), 27.03 (tBu-Me), 19.40 (tBu-C), 19.34 (tBu-C), 19.29 (tBu-C), 19.27 (tBu-C).

HRMS (MALDI) m/z: $[M + Na]^+$ Calcd for $C_{65}H_{74}N_6O_9Si_2Na^+$ 1161.4948; Found 1161.4931.

Spectra can be seen on page S80 - S83.

S1.20 *O*-(2-deoxy-2-azido-3,4-di-*O*-benzyl-6-*O*-*tert*-butyldiphenylsilyl-α-D-galactopyranosyl)(1→3)-2-deoxy-2-azido-4-*O*-benzyl-6-*O*-*tert*-butyldiphenylsilyl-α/β-D-galactopyranosyl) Trichloroacetimidate, (28)

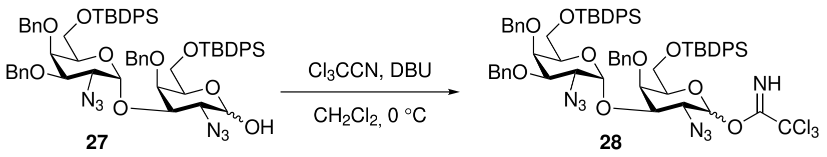

The free 1-OH **27** (371 mg, 326 µmol) was dissolved in dry CH_2Cl_2 (25 mL), cooled to 0 °C before adding Cl_3CCN (0.16 mL, 1.6 mmol, 5 equiv.) followed by DBU (2 drops, cat.). After 1.5 h the reaction mixture was evaporated *in vacuo*. The crude product was purified by flash column chromatography (SiO_2, short, CH_2Cl_2 0.5% Et_3N) to yield the pure title compound **28** (361 mg, 281 µmol, 86%, α:β 1:1)(R_f 0.94 CH_2Cl_2) as a clear oil.

Characterisation

^{1}H NMR (500 MHz, $CDCl_3$) δ 8.72 (s, 1H, =NHβ), 8.62 (s, 1H, =NHα), 7.69-7.56 (m, 25H), 7.49-7.27 (m, 80H), 6.45 (d, J = 3.0 Hz, 1H, H-1α), 5.57 (d, J = 8.4 Hz, 1H, H-1β), 5.27 (d, J = 3.5 Hz, 1H, H-1'α), 5.24 (d, J = 3.4 Hz, 1H, H-1'β), 5.14 (d, J = 10.8 Hz, 1H, O4-Bnα), 5.07 (d, J = 10.8 Hz, 1H, O4-Bnβ), 4.98 (d, J = 10.9 Hz, 1H, O4'-Bnα), 4.95 (d, J = 11.2 Hz, 1H, O4'-Bnβ), 4.80 (s, 4H, O3'-Bn O3'-Bn), 4.73 (d, J = 10.9 Hz, 1H, O4-Bnα), 4.63 (t, J = 10.8 Hz, 3H, O4'-Bn O4-Bn O4'-Bn), 4.27 (br s, 1H, H-4), 4.24 (br s, 1H, H-4), 4.23-4.04 (m, 16H, H-2 H-2' H-2' H-2), 4.04-4.00 (m, 1H, H-5), 3.96 (t, J = 9.5 Hz, 1H, H-6a), 3.90-3.70 (m, 11H, H-6b H-6' H-6 H-6' H-5), 3.58 (t, J = 6.8 Hz, 1H, H-5β), 1.09 (s, 9H), 1.08 (s, 9H), 1.06 (s, 9H), 1.04 (s, 9H).

^{13}C NMR (125 MHz, $CDCl_3$) δ 161.2 (C=NHβ), 160.9 (C=NHα), 138.8, 138.5, 138.4, 138.3, 137.7, 137.5, 135.71, 135.65, 135.62, 135.60, 135.57, 135.50, 133.3, 133.22, 133.19, 133.07, 133.05, 133.01, 130.0, 129.98, 129.96, 128.73, 128.69, 128.5, 128.43, 128.41, 128.36, 128.36, 128.27, 128.20, 128.1, 127.97, 127.95, 127.94, 127.92, 127.91, 127.87, 127.82, 127.78, 127.77, 127.71, 127.6 (84 C-Ar), 97.2 (C-1β), 95.9 (C-1'), 95.1 (C-1'), 94.8 (C-1α), 91.1 (CCl_3), 90.7 (CCl_3), 77.7, 76.0 (C-5), 75.9 (C-5β), 75.2 (O4-Bnα), 75.1 (O4-Bn), 75.0 (O4'-Bn), 74.6, 73.6, 73.6, 72.7 (O3'-Bn), 72.6 (O3'-Bn), 72.3, 71.9, 71.5, 70.5, 62.4 (C-6), 62.3 (C-2), 61.9 (C-6), 61.8 (C-6), 61.5 (C-6), 60.0 (C-2), 59.8 (C-2), 59.5 (C-2), 27.1 (tBu-Me), 27.02 (tBu-Me), 26.99 (tBu-Me), 26.98 (tBu-Me), 19.40 (tBu-C), 19.35 (tBu-C), 19.28 (tBu-C), 19.26 (tBu-C).

HRMS (ESI) m/z: $[M + Na]^+$ Calcd for $C_{67}H_{74}Cl_3N_7O_9Si_2Na^+$ 1304.4044; Found 1304.4004.

Spectra can be seen on page S84 - S87.

S1.21 5-*O*-allyl-1-*O*-(3-*O*-[2-azido-3,4-di-*O*-benzyl-6-*O*-(*tert*-butyldiphenylsilyl)-2-deoxy-α-D-galactopyranosyl]-2-azido-4-*O*-benzyl-6-*O*-(*tert*-butyldiphenylsilyl)-2-deoxy-β-D-galactopyranosyl)-2,3,4-tri-*O*-benzyl-D-ribitol, (29)

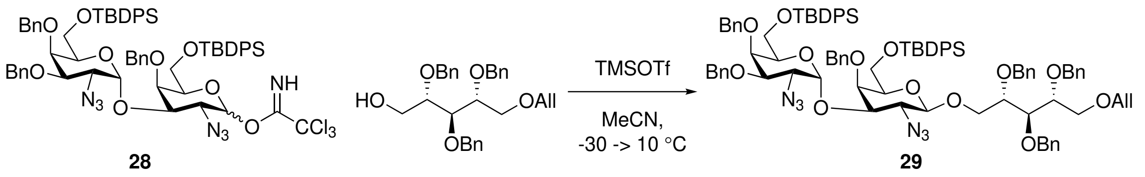

Donor (**28**) (30 mg, 23 µmol) and acceptor (2,3,4-tri-*O*-benzyl-D-ribitol[12]) (20 mg, 43 µmol, 1.9 equiv.) was dissolved in dry MeCN (0.5 mL) and stirred with 3 Å molecular sieves for 2 h. The reaction mixture was then cooled to -30 °C and added TMSOTf (1 drop, 1-2 µL, cat.) and let slowly warm to 10 °C over 3 h. The reaction mixture was quenched with Et_3N (0.05 mL), filtrated through a pad of celite eluting with CH_2Cl_2 and evaporated *in vacuo*. The crude product was purified by flash column chromatography (SiO_2, CH_2Cl_2 : PE 2:1) to yield the pure title compound **29** (27 mg, 17 µmol, 74%, β)(R_f 0.25 CH_2Cl_2 : PE 2:1) as a clear oil.

Characterisation

^{1}H NMR (500 MHz, $CDCl_3$) δ 7.72-7.54 (m, 10H), 7.48-7.24 (m, 36H), 7.25-7.16 (m, 18H), 5.88 (ddt, J = 17.2, 10.7, 5.5 Hz, 1H, C*H*=), 5.29-5.20 (m, 2H, H-1" =CH_2a), 5.17-5.11 (m, 1H, =C*H*b), 5.08 (d, J = 10.9 Hz, 1H, O4'-Bn), 4.92 (d, J = 11.2 Hz, 1H, O4"-Bn), 4.80-4.77 (m, 2H, O3"-Bn), 4.75-4.72 (m, 1H, OBn), 4.72-4.69 (m, 1H, OBn), 4.69-4.61 (m, 6H, O4'-Bn), 4.59-4.54 (m, 3H, O4"-Bn), 4.23 (t, J = 6.6 Hz, 1H, H-5"), 4.21-4.13 (m, 3H, H-1' H-3"), 4.09-4.03 (m, 5H, H-1a H-2"), 3.99-3.91 (m, 4H, =CH_2−All H-2), 3.91-3.87 (m, 3H, H-3 H-4), 3.87-3.82 (m, 3H, H-2'), 3.83-3.78 (m, 2H, H-1b), 3.78-3.72 (m, 3H, H-6' H-6"), 3.69 (dd, J = 10.6, 3.3 Hz, 1H), 3.67-3.60 (m, 3H, H-5), 3.34 (dd, J = 8.0, 5.9 Hz, 1H, H-5'), 1.06 (s, 9H), 1.04 (s, 9H).

^{13}C NMR (125 MHz, $CDCl_3$) δ 138.8, 138.7, 138.63, 138.56, 138.4, 137.6, 135.7, 135.63, 135.62, 135.1 (*C*H=), 133.5, 133.14, 133.10, 133.07, 129.99, 129.96, 129.93, 128.7, 128.4, 128.33, 128.29, 128.19, 128.14, 127.99, 127.95, 127.94, 127.91, 127.8, 127.7, 127.6, 127.54, 127.47 (60 C-Ar), 116.7 (=*C*H_2), 102.3 (C-1'), 94.5 (C-1"), 78.8 (C-2/C-3/C-4), 78.7 (C-2/C-3/C-4), 78.2 (C-2/C-3/C-4), 77.8 (C-3"), 75.8, 75.0 (O4"-Bn), 74.85 (O4'-Bn), 74.79 (C-5'), 74.0 (OBn), 73.7, 72.7 (3"-OBn), 72.6 (OBn), 72.5 (OBn), 72.3 (*C*H_2−All), 71.9 (C-5"), 70.6, 70.4 (C-5), 69.2 (C-1), 63.0 (C-2'), 62.8 (C-6"), 61.9 (C-6'), 59.9 (C-2"), 27.0 (*t*Bu-Me), 19.4 (*t*Bu-C), 19.3 (*t*Bu-C).

HRMS (MALDI) m/z: $[M + Na]^+$ Calcd for $C_{94}H_{106}N_6O_{13}Si_2Na^+$ 1605.7249; Found 1605.7225.

Spectra can be seen on page S88 - S92.

S2 Spectra

S2.1 Spectra for 1

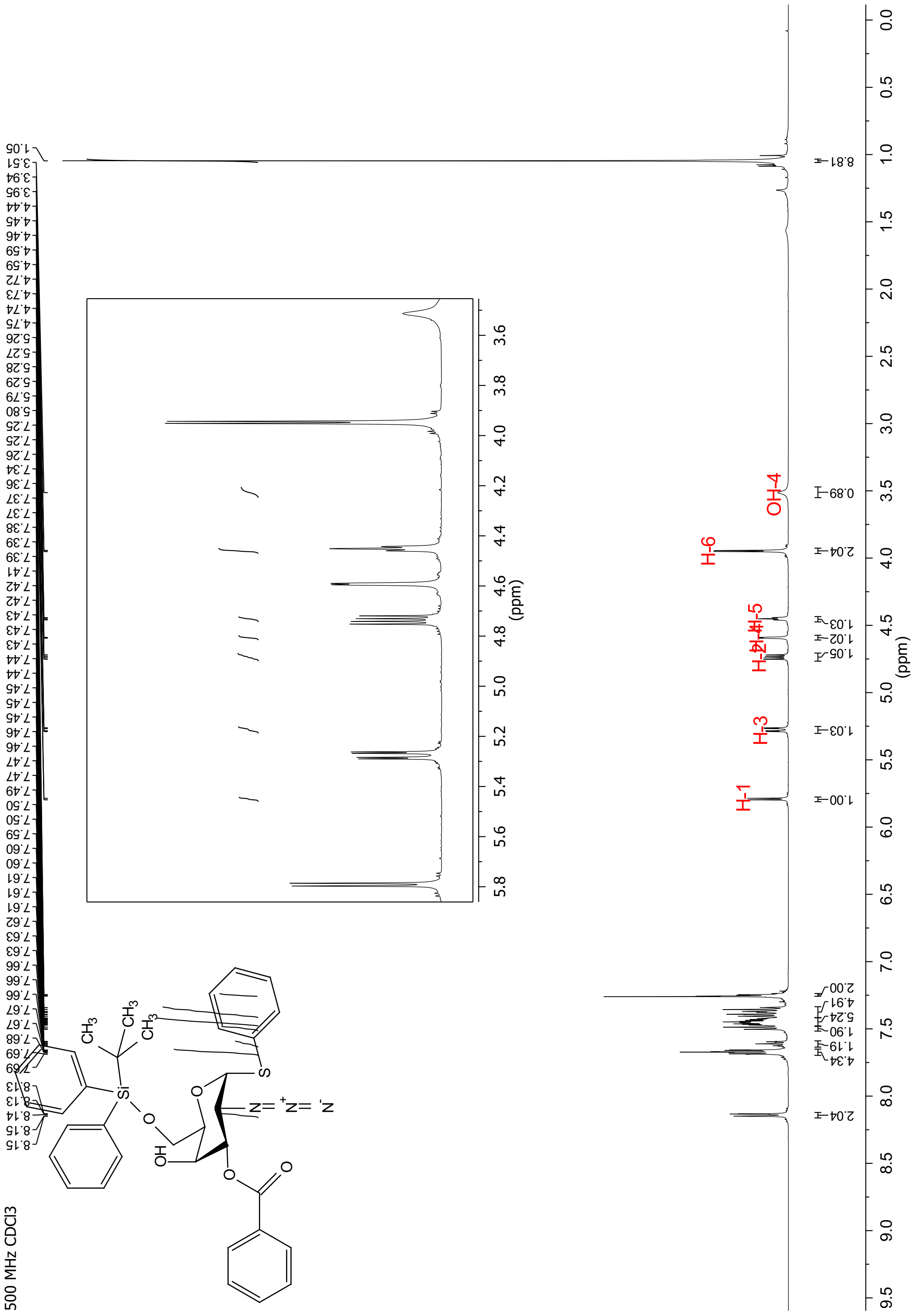

Figure S1: ^{1}H NMR (500 MHz, $CDCl_3$)

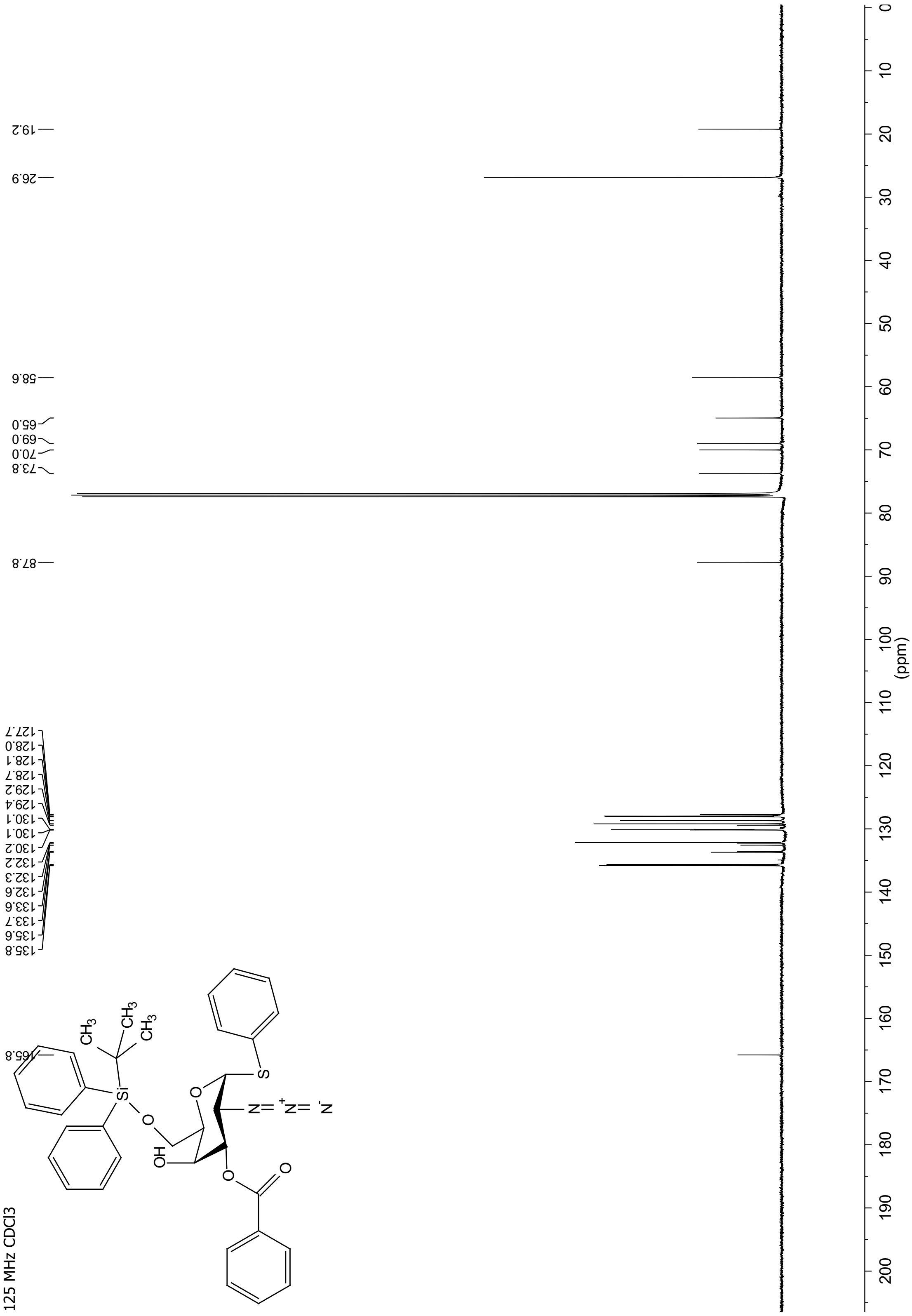

Figure S2: ^{13}C NMR (125 MHz, $CDCl_3$)

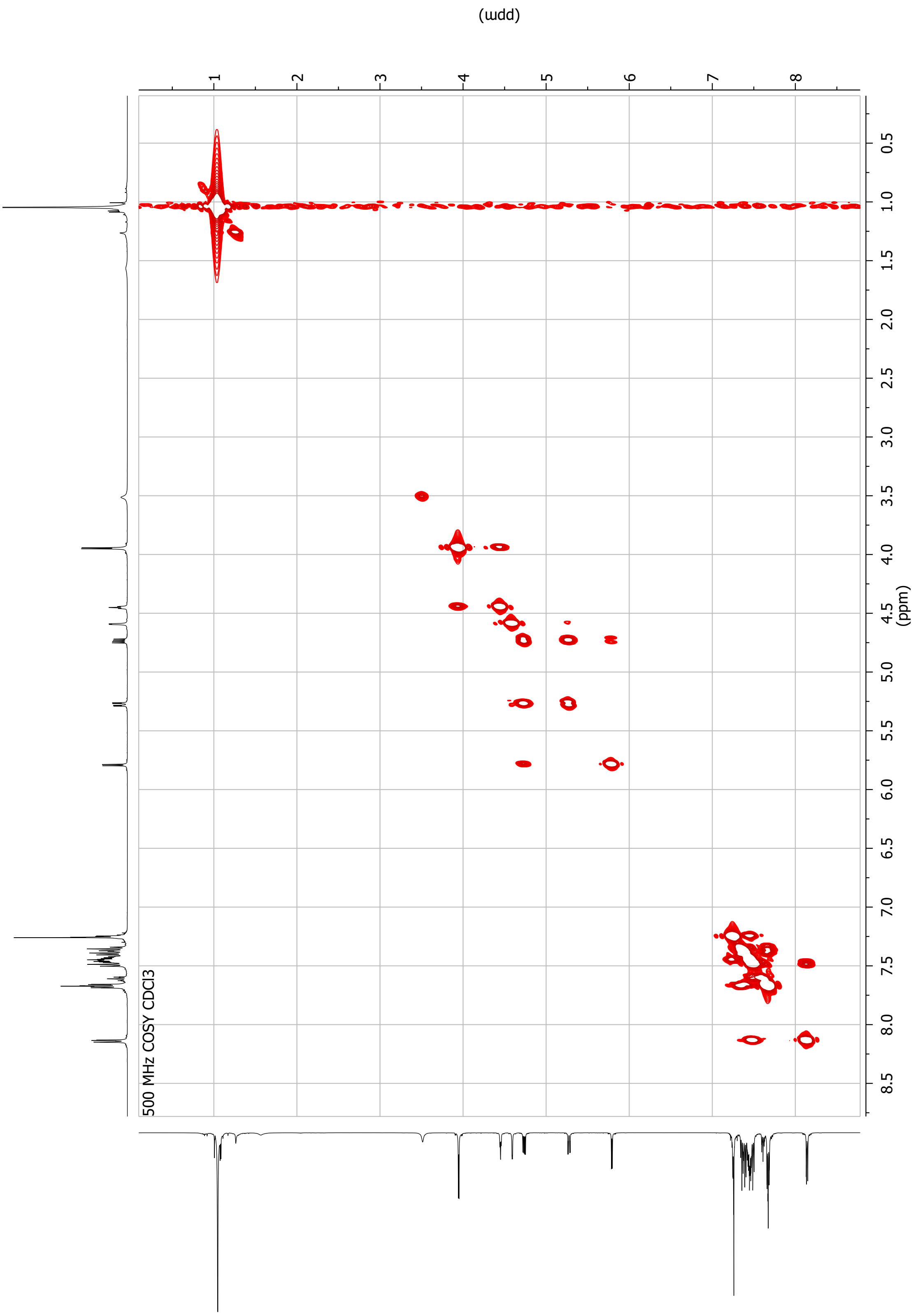

Figure S3: COSY NMR (500 MHz, $CDCl_3$)

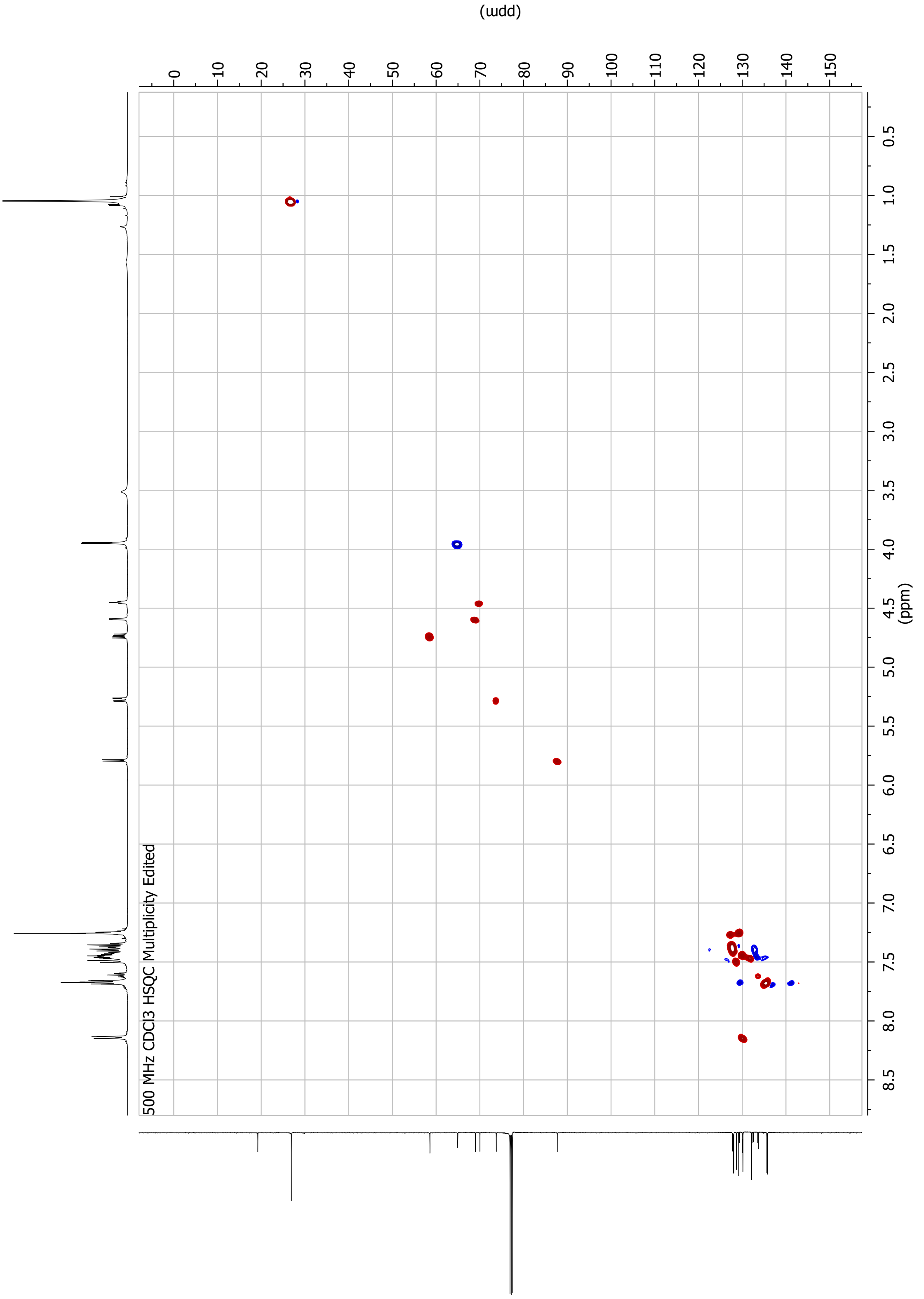

Figure S4: HSQC Multiplicity Edited NMR (500 MHz, $CDCl_3$)

S2.2 Spectra for 9

Spectral data (peak assignment) were not in full agreement with literature.[2]

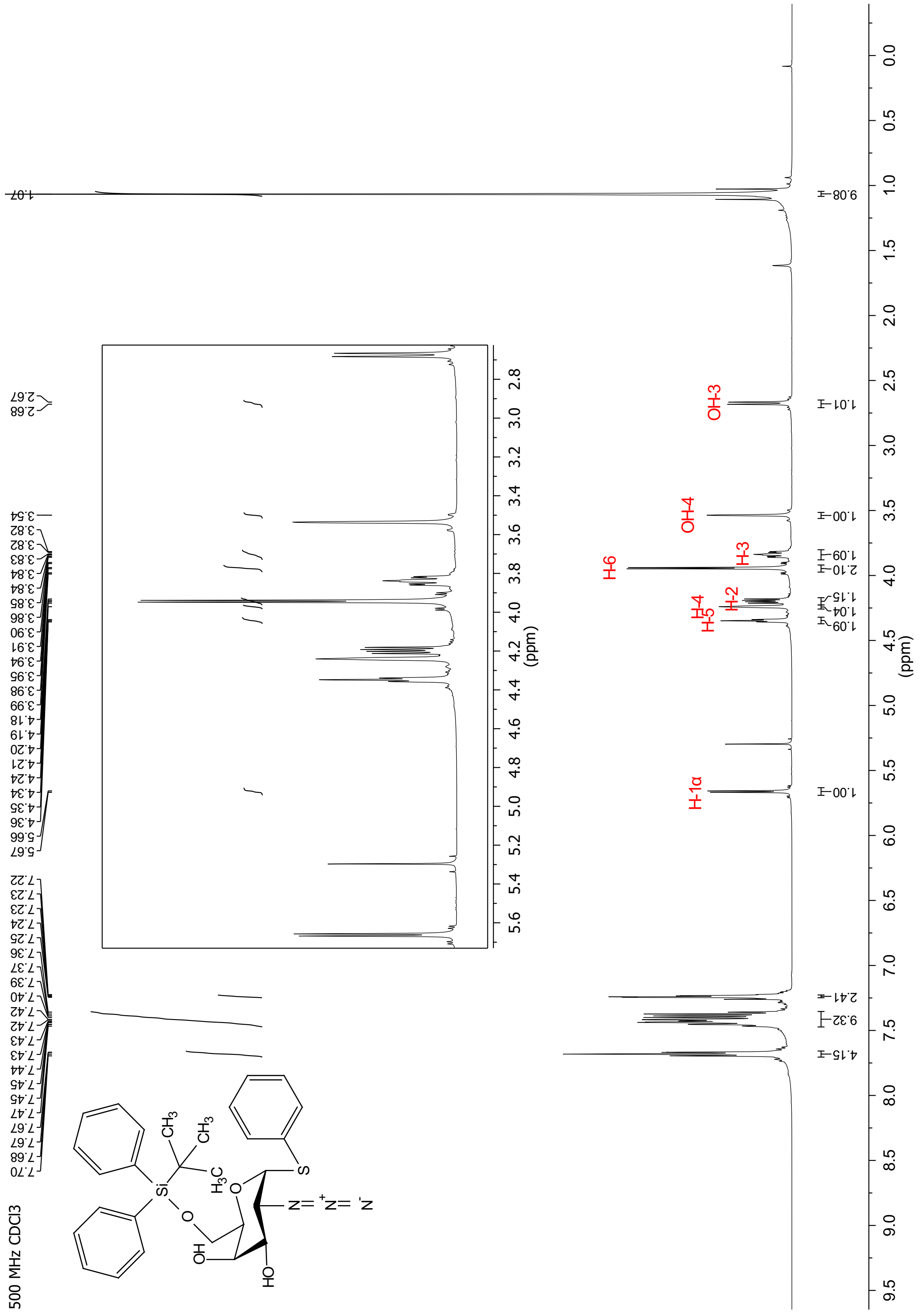

Figure S5: ^{1}H NMR (500 MHz, $CDCl_3$)

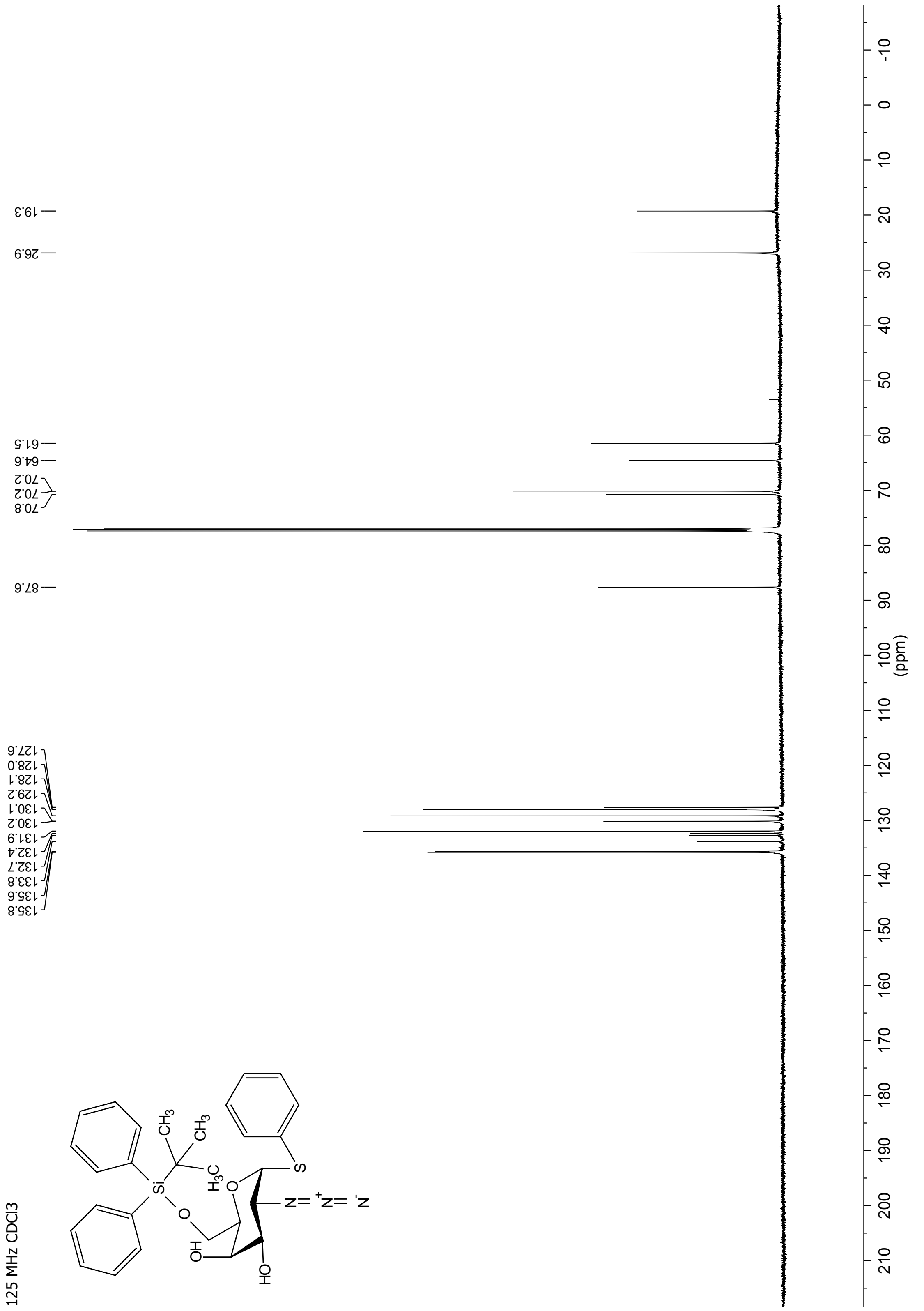

Figure S6: ^{13}C NMR (125 MHz, $CDCl_3$)

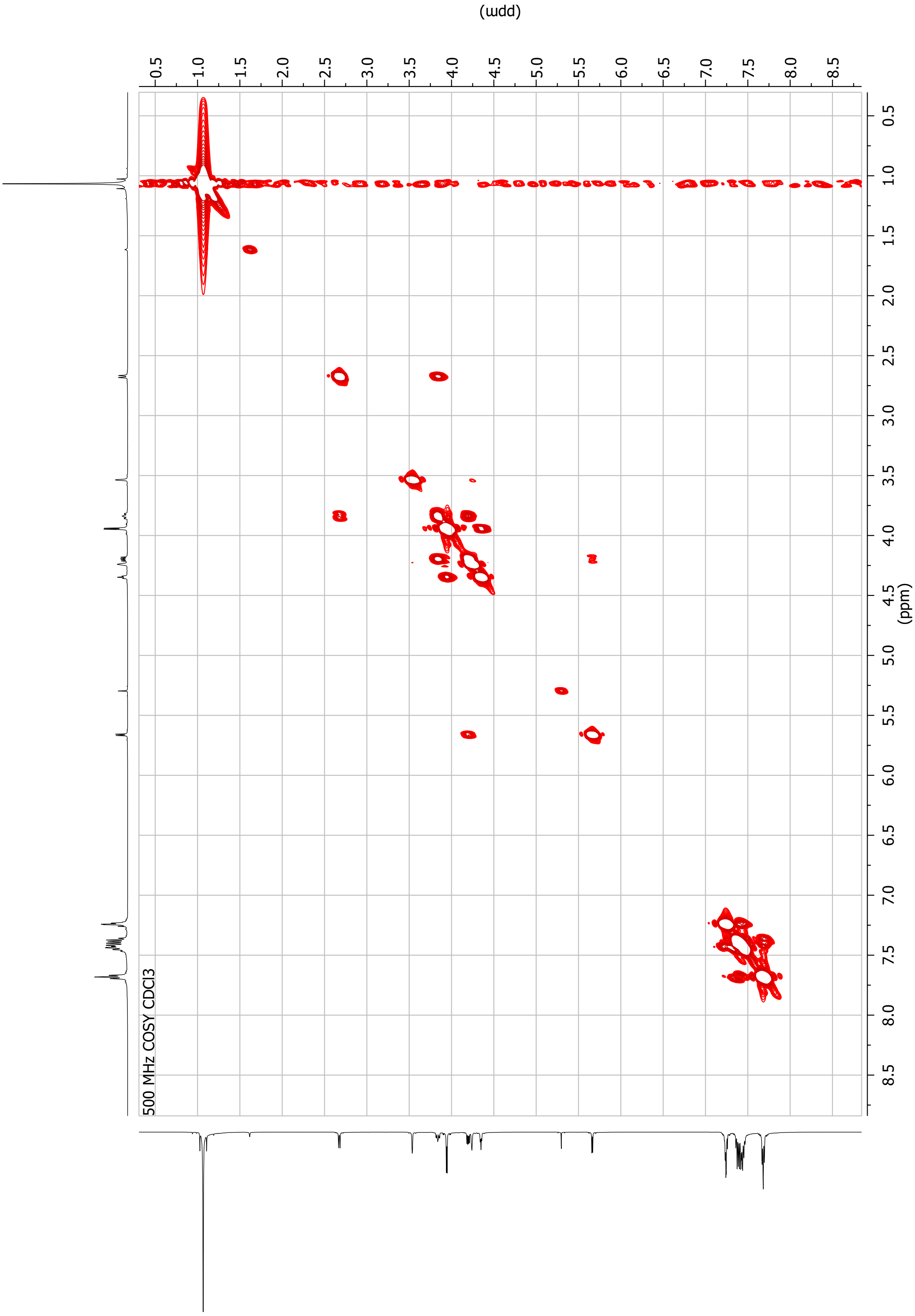

Figure S7: COSY NMR (500 MHz, $CDCl_3$)

S2.3 Spectra for 11

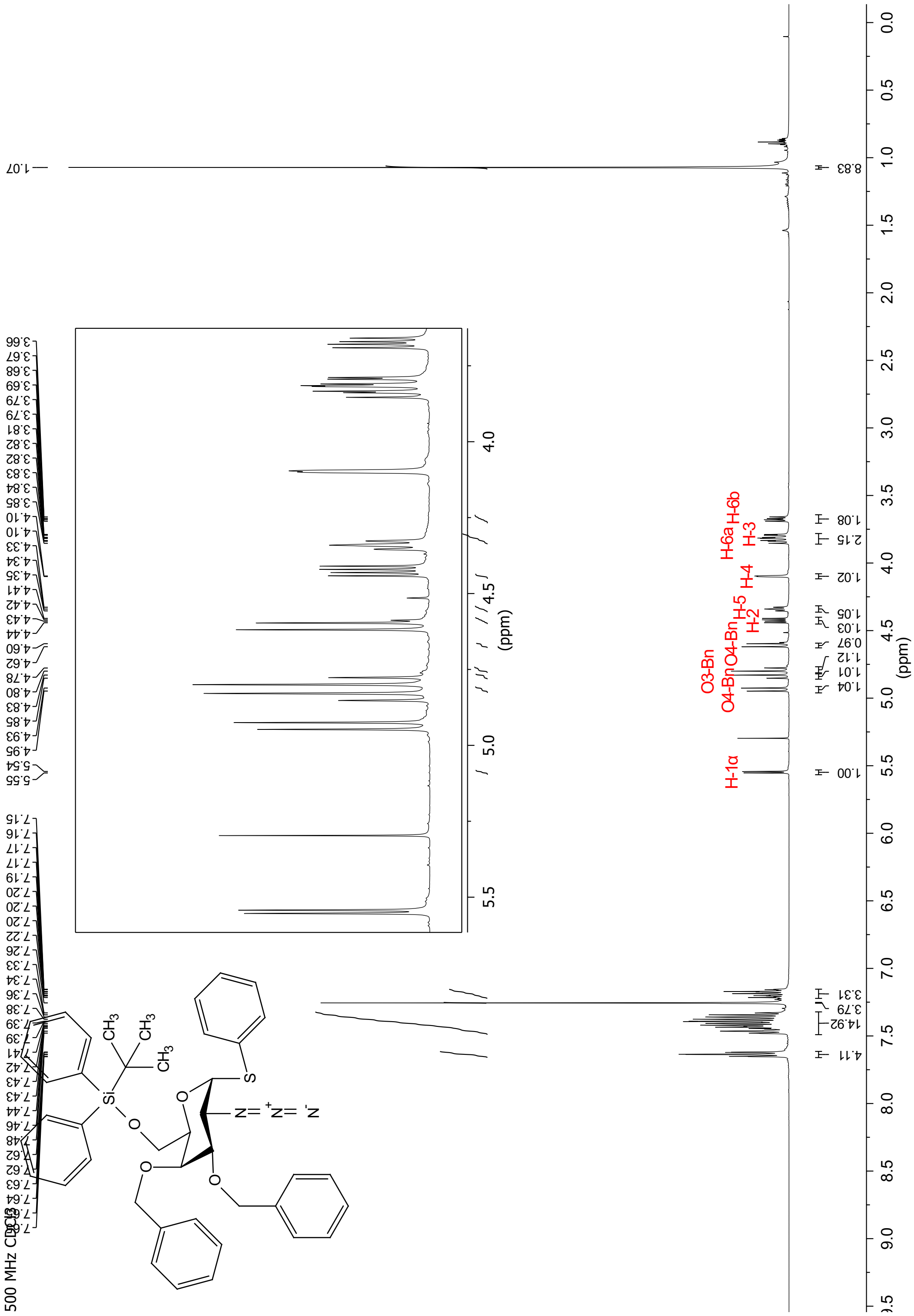

Figure S8: ^{1}H NMR (500 MHz, $CDCl_3$)

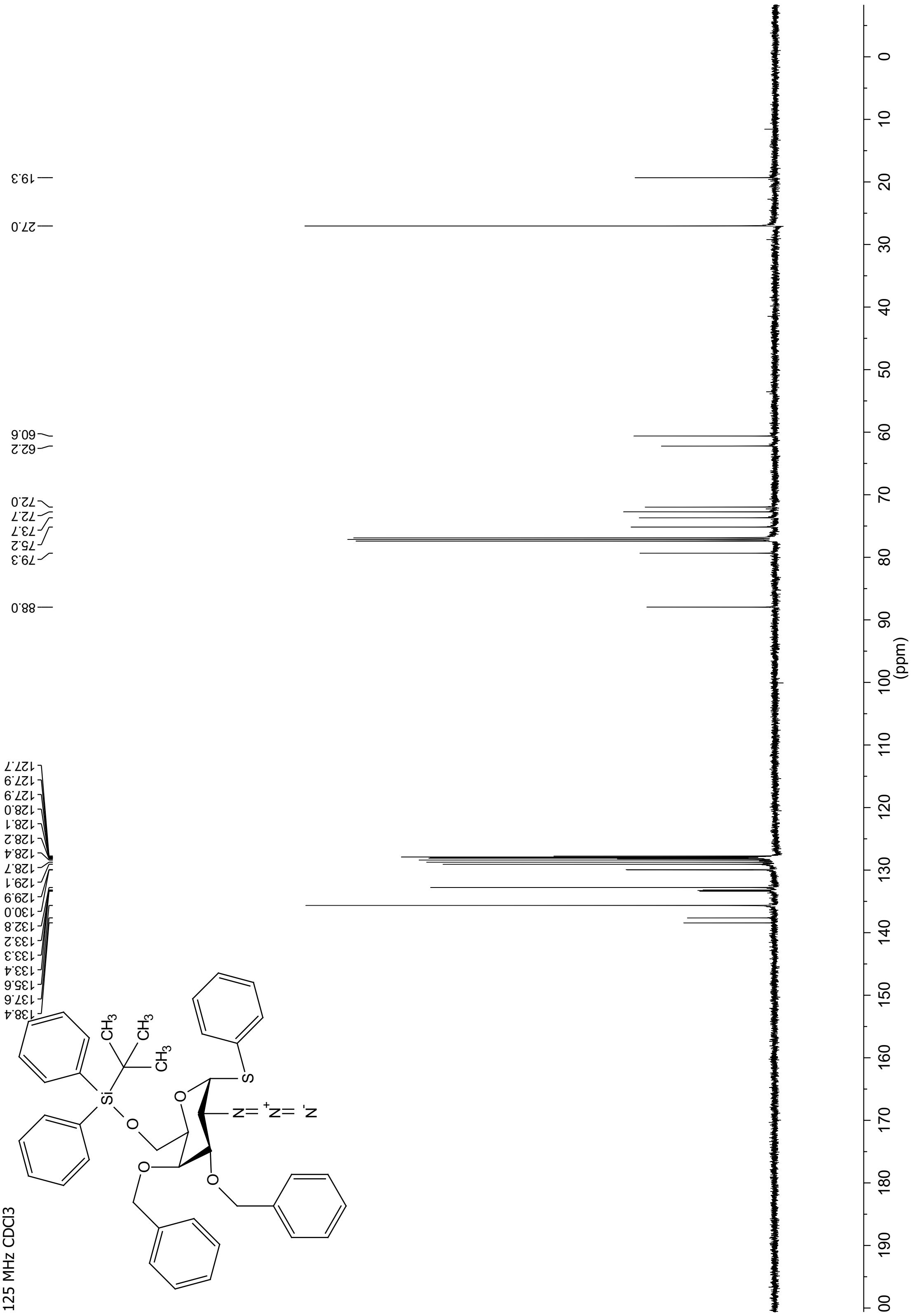

Figure S9: ^{13}C NMR (125 MHz, $CDCl_3$)

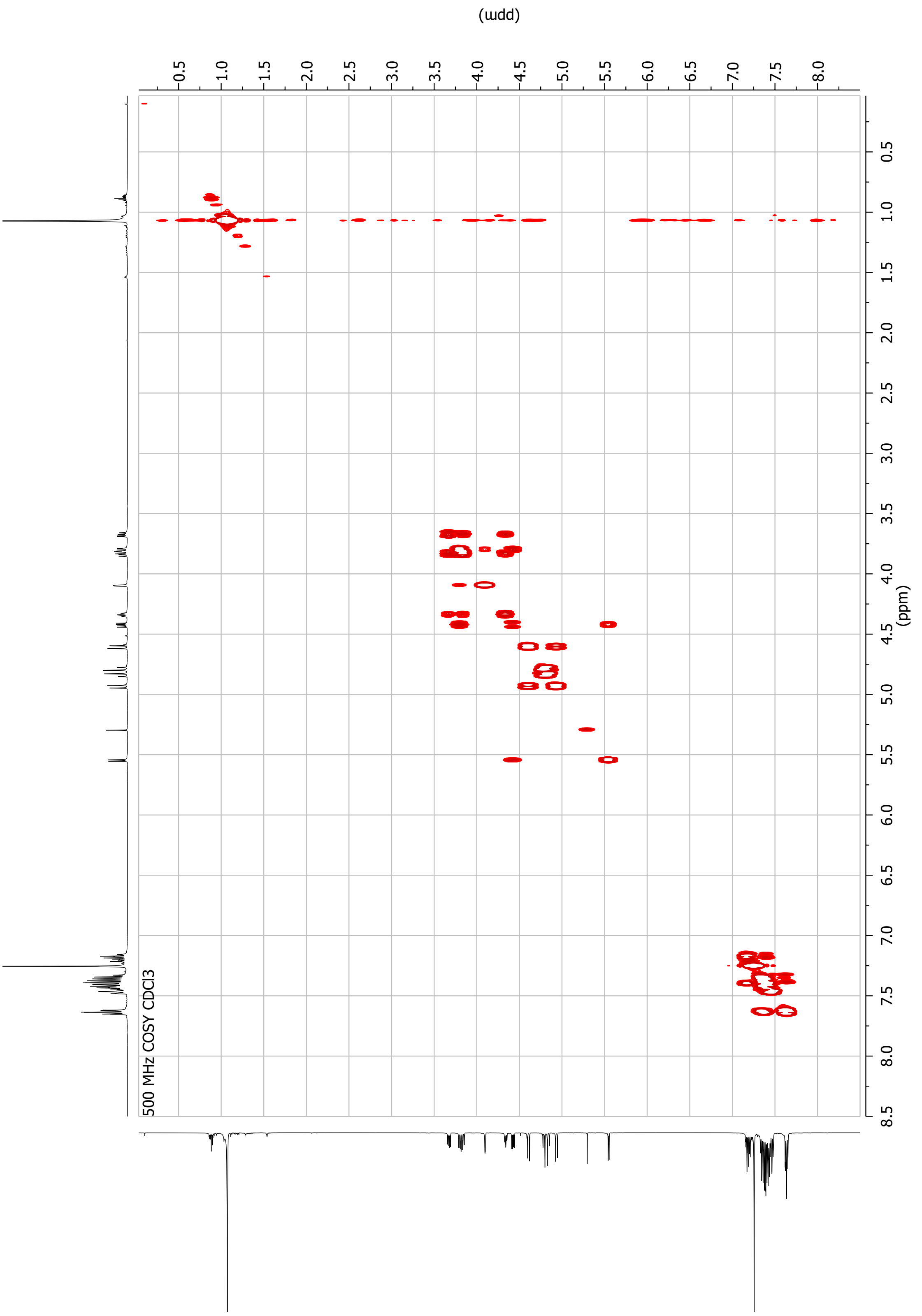

Figure S10: COSY NMR (500 MHz, $CDCl_3$)

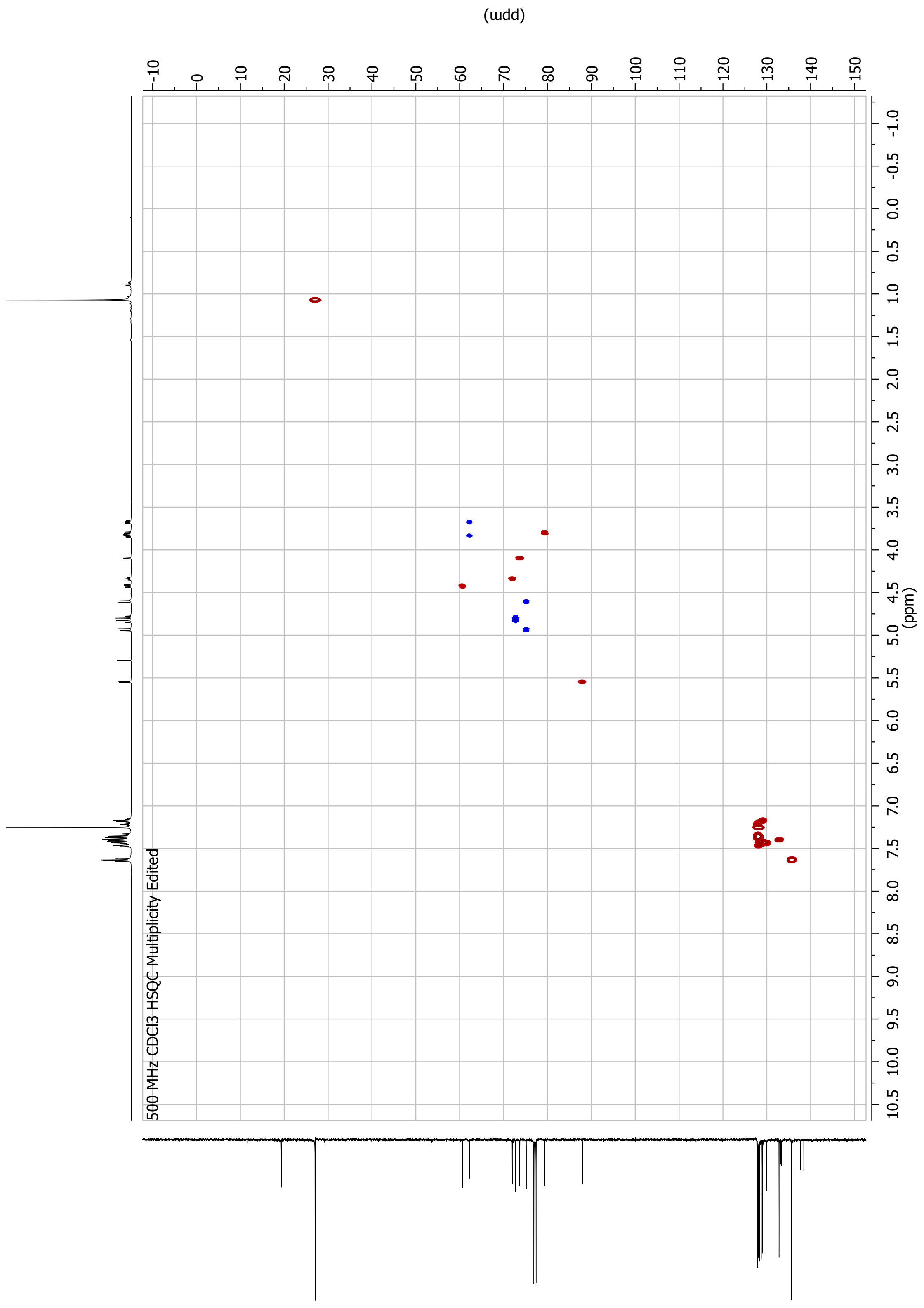

Figure S11: HSQC Multiplicity Edited NMR (500 MHz, $CDCl_3$)

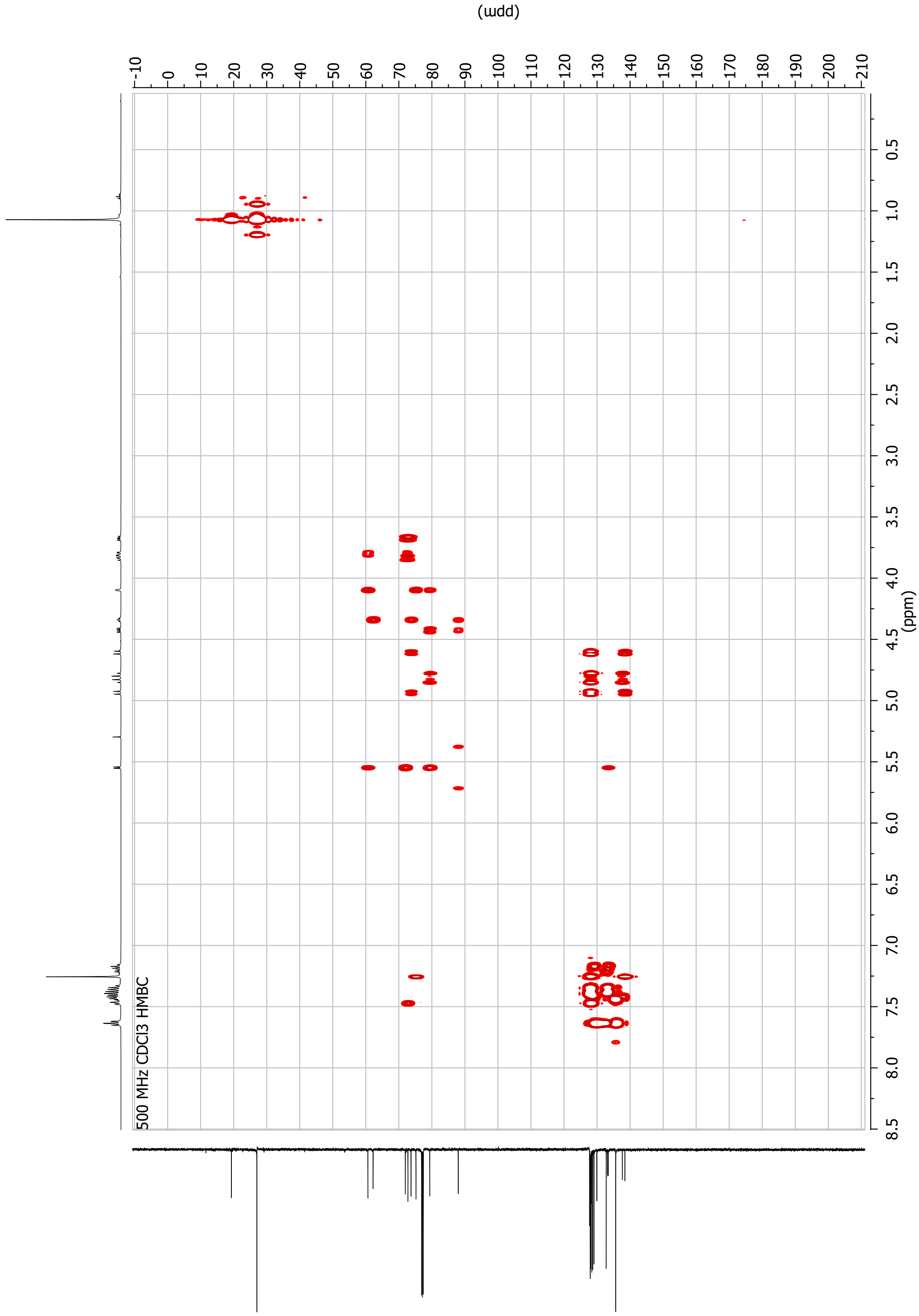

Figure S12: HMBC NMR (500 MHz, $CDCl_3$)

S2.4 Spectra for 15

Compound was wrongly reported in literature twice and spectral data were therefore not in accordance.[4,5]

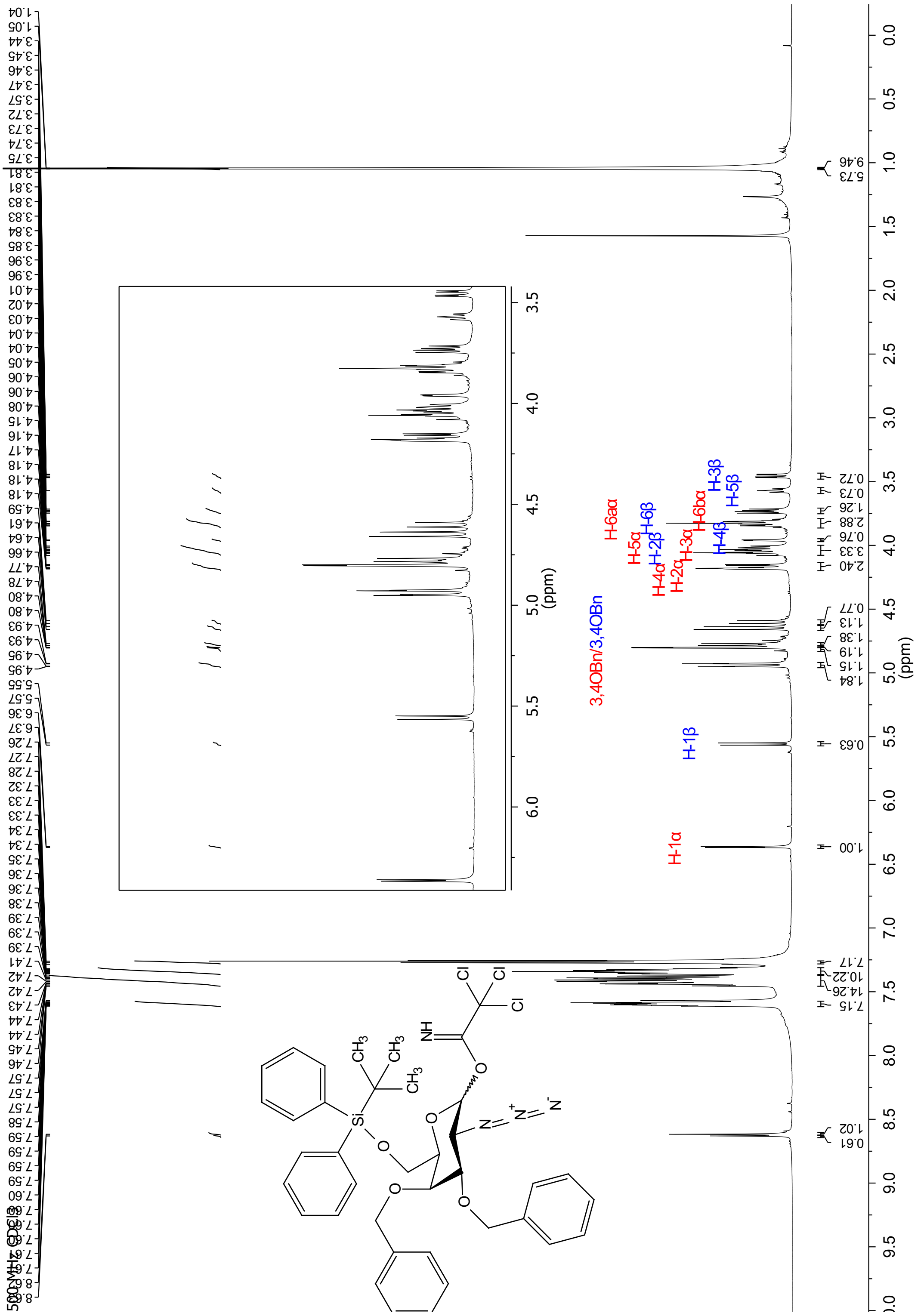

Figure S13: ^{1}H NMR (500 MHz, $CDCl_3$)

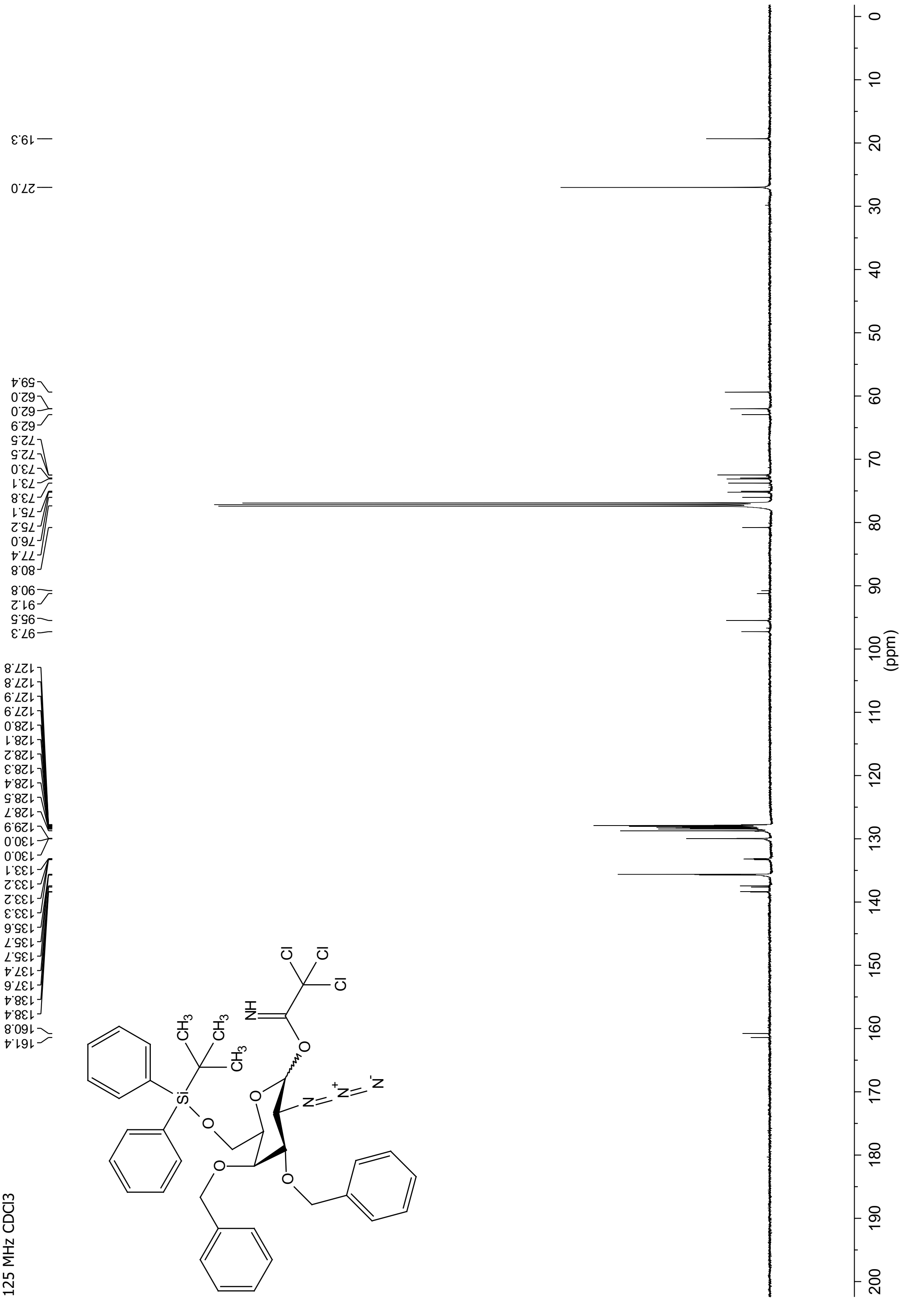

Figure S14: ^{13}C NMR (125 MHz, $CDCl_3$)

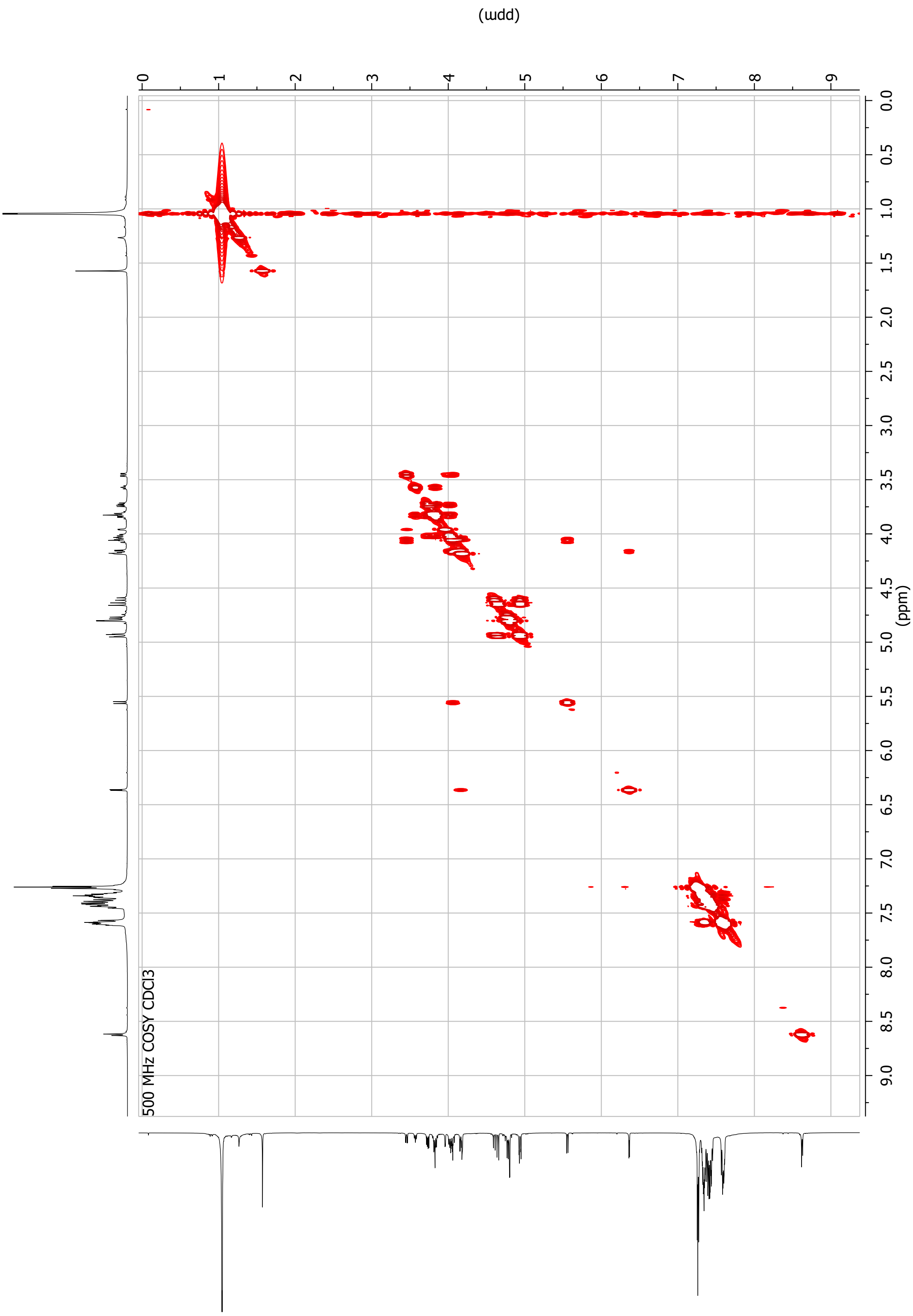

Figure S15: COSY NMR (500 MHz, $CDCl_3$)

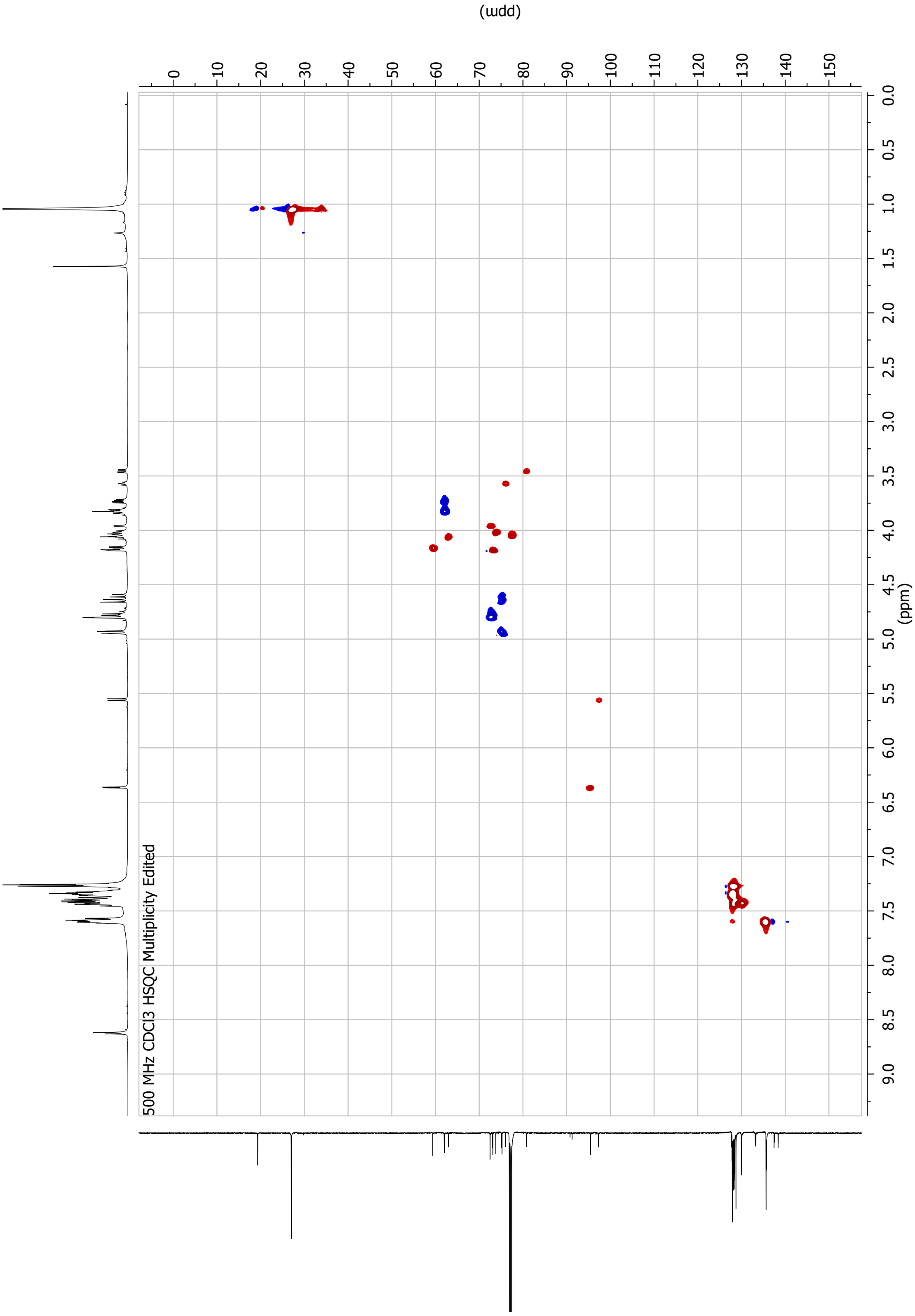

Figure S16: HSQC Multiplicity Edited NMR (500 MHz, $CDCl_3$)

S2.5 Spectra for 12α/β

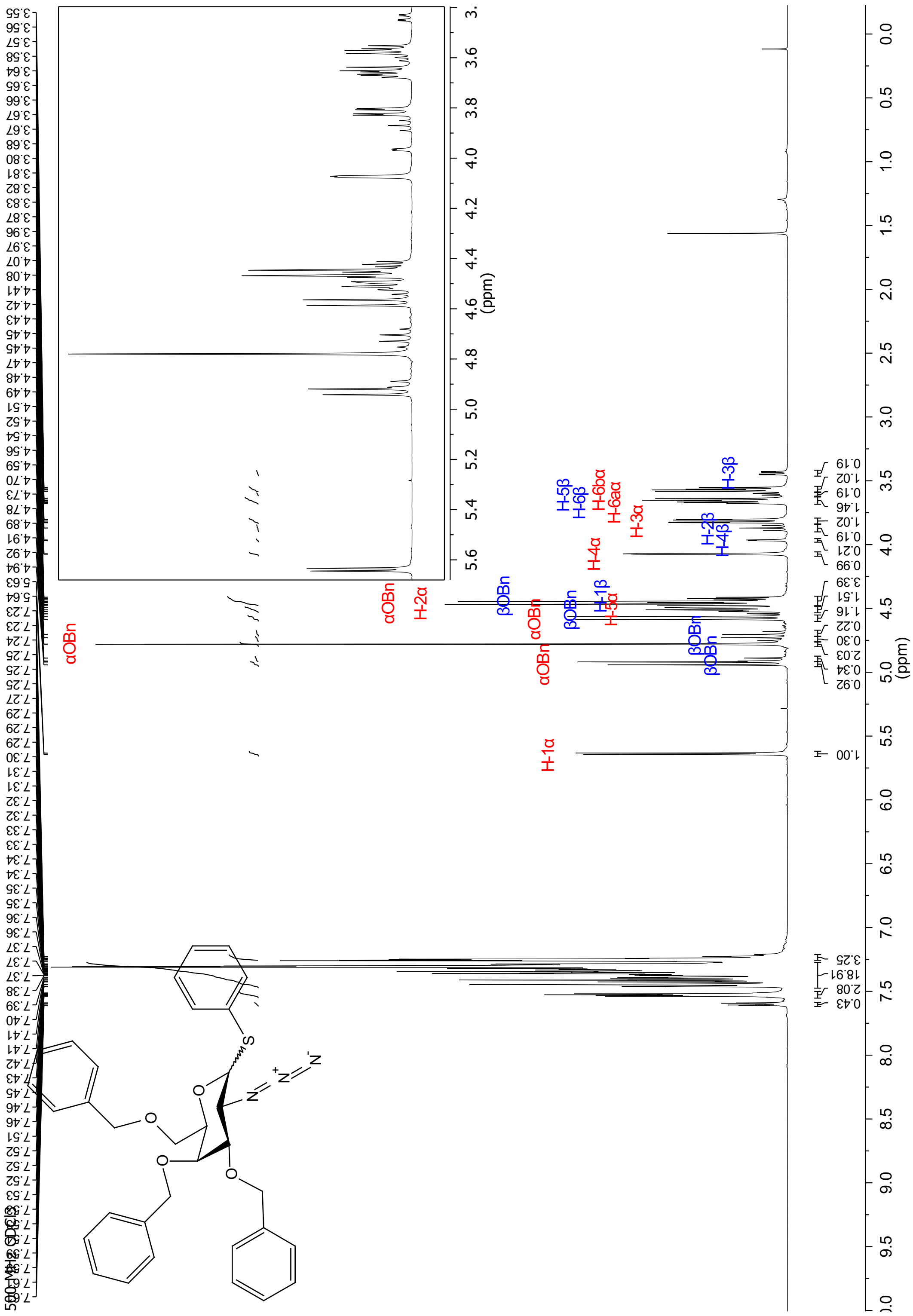

Figure S17: ^{1}H NMR (500 MHz, $CDCl_3$)

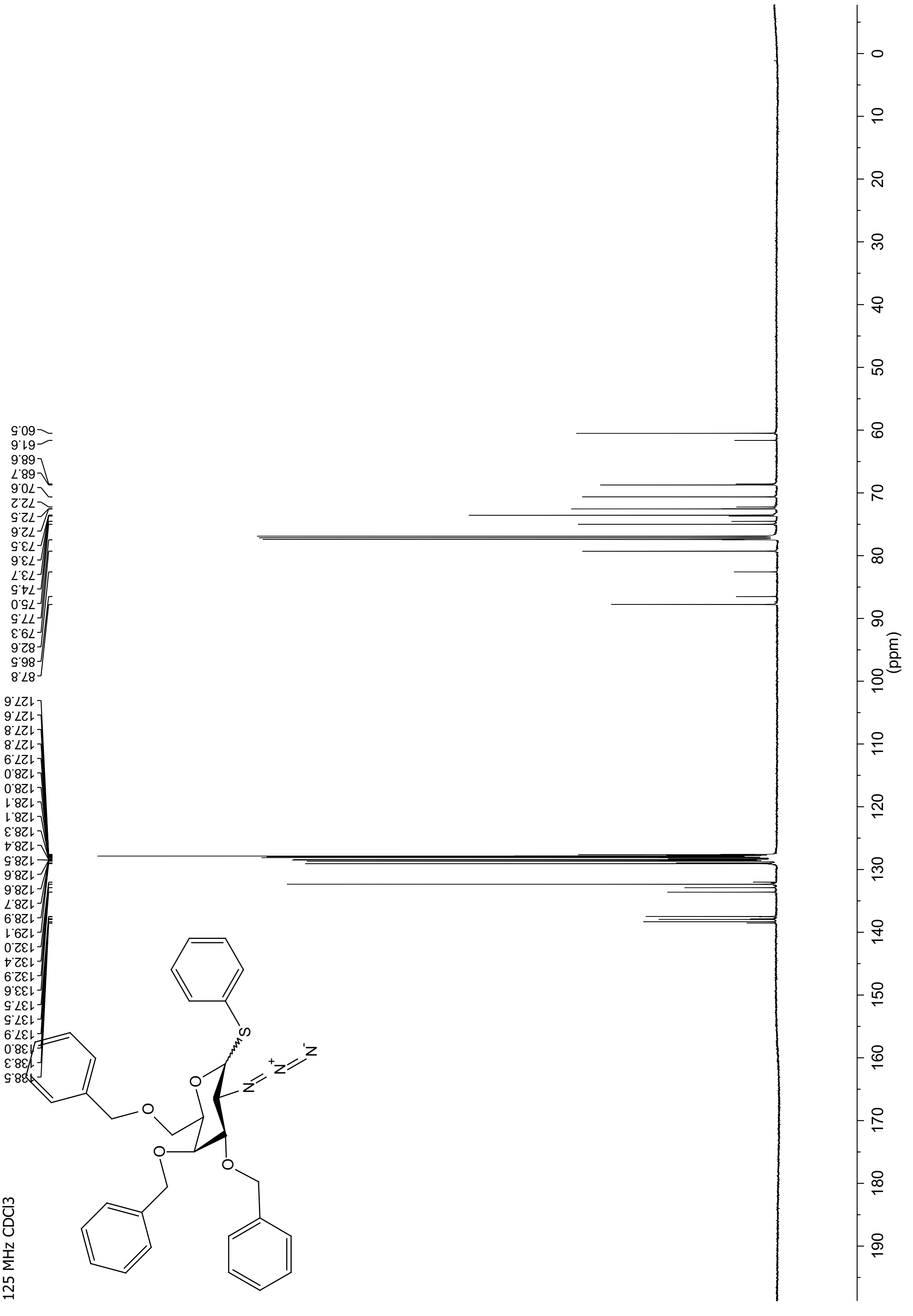

Figure S18: ^{13}C NMR (125 MHz, $CDCl_3$)

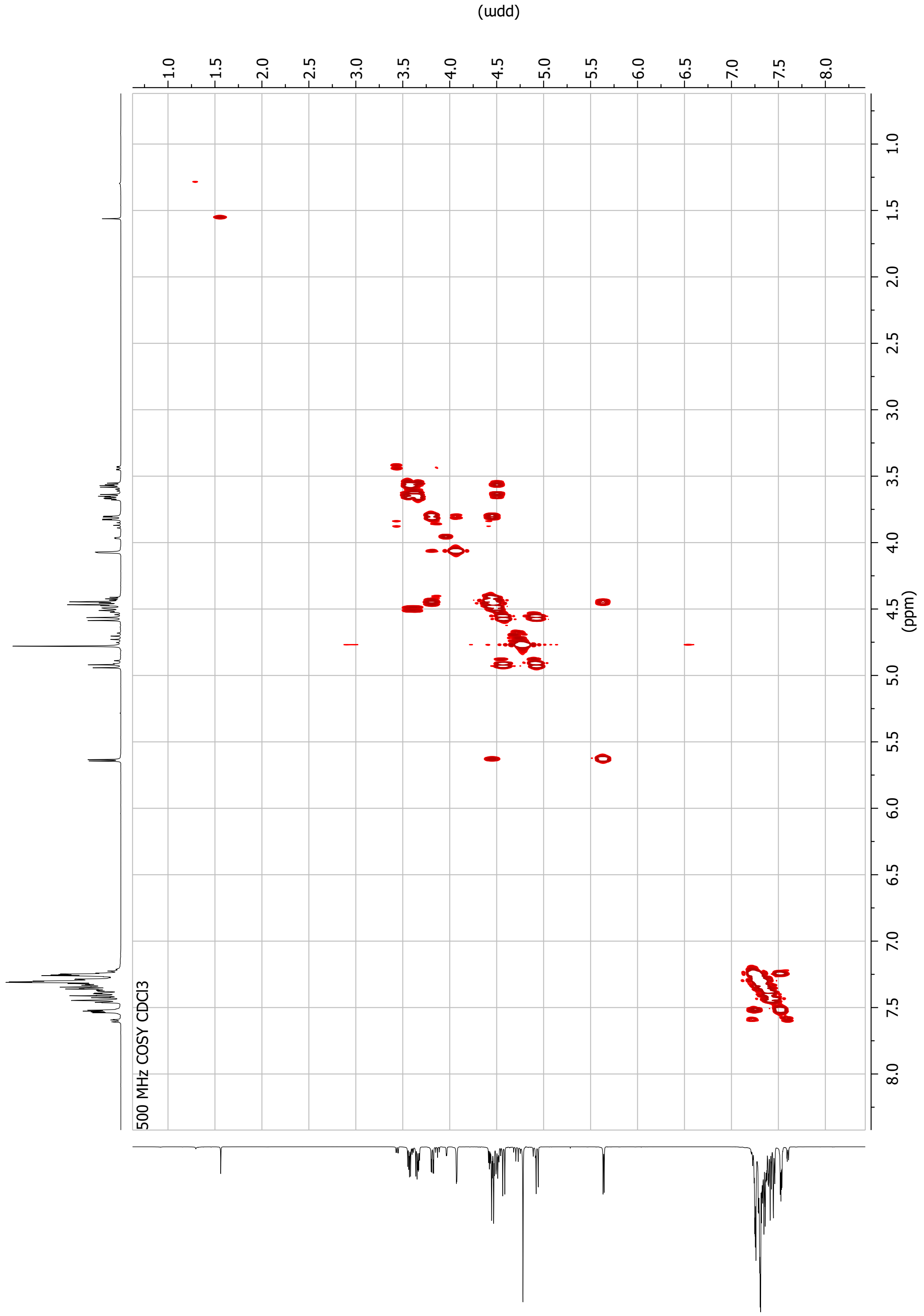

Figure S19: COSY NMR (500 MHz, $CDCl_3$)

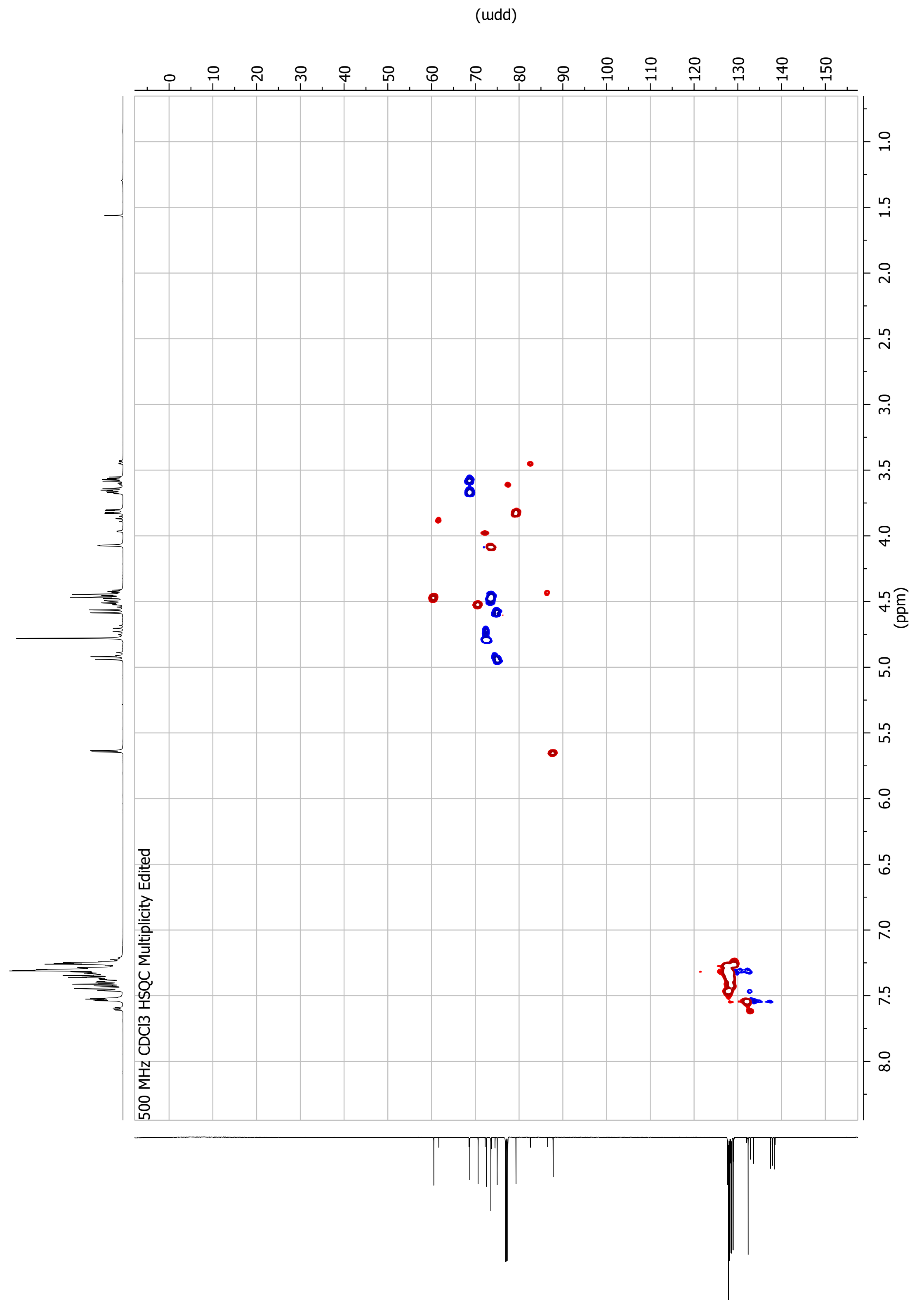

Figure S20: HSQC Multiplicity Edited NMR (500 MHz, $CDCl_3$)

S2.6 Spectra for 17

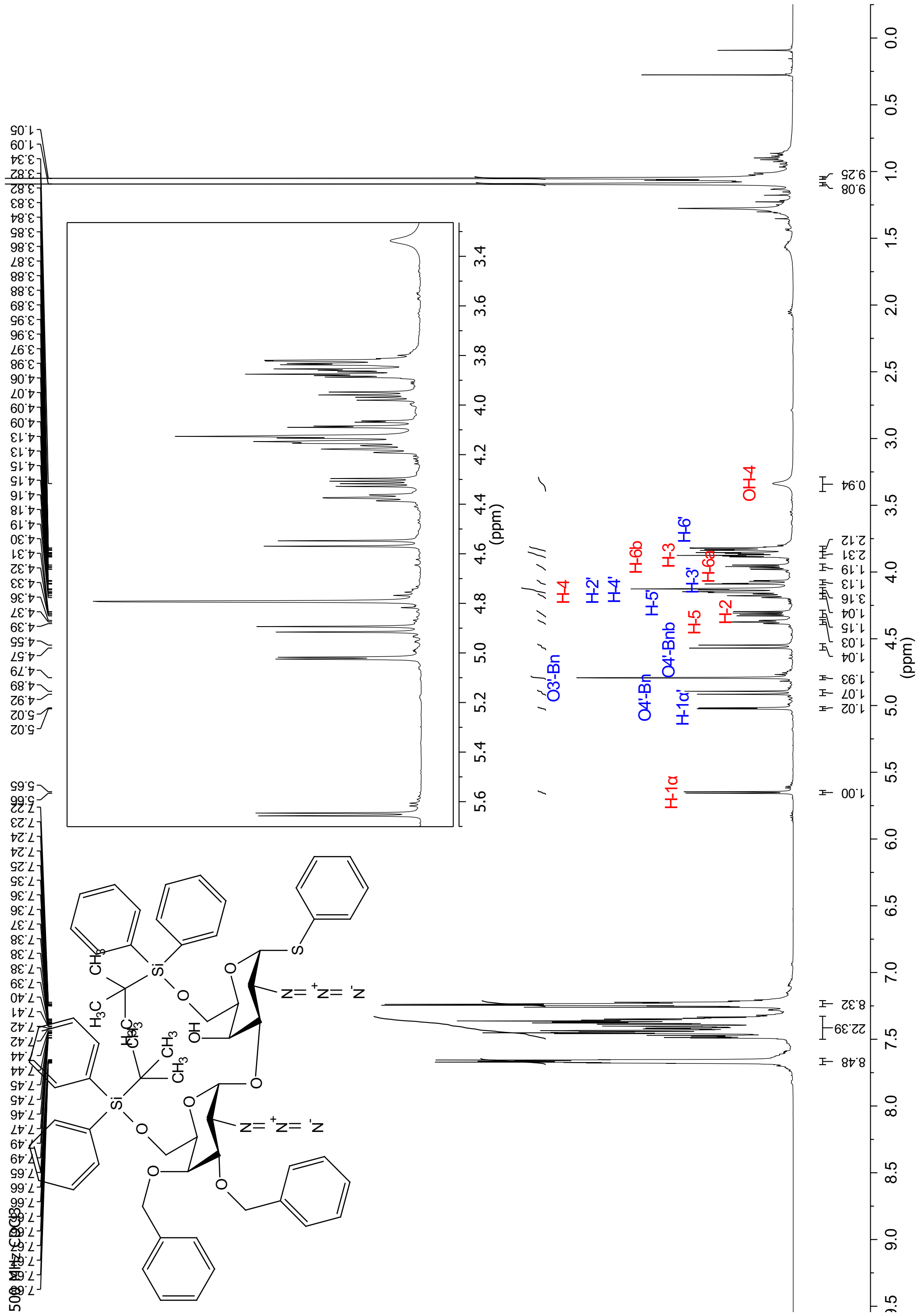

Figure S21: ^{1}H NMR (500 MHz, $CDCl_3$)

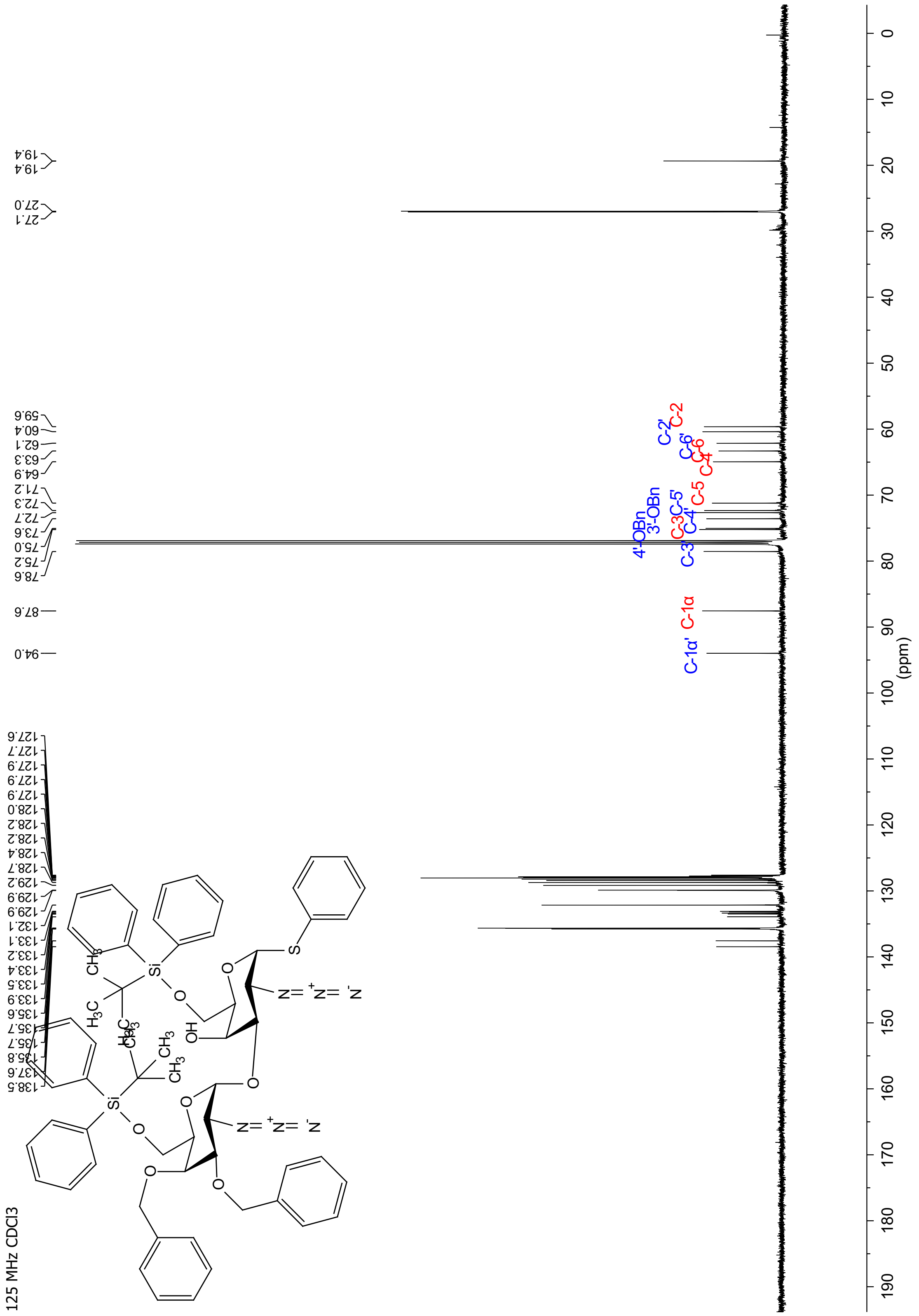

Figure S22: ^{13}C NMR (125 MHz, $CDCl_3$)

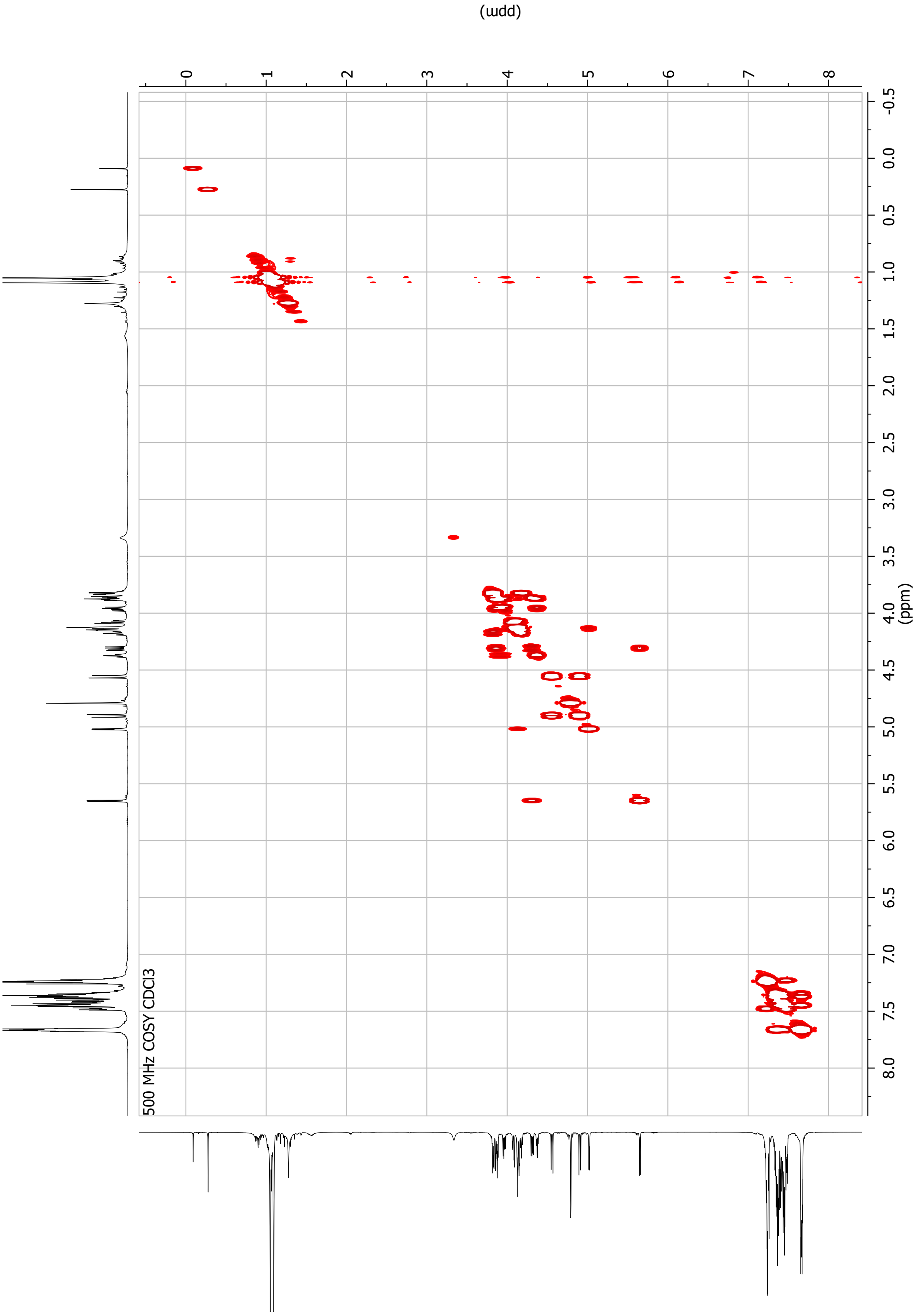

Figure S23: COSY NMR (500 MHz, $CDCl_3$)

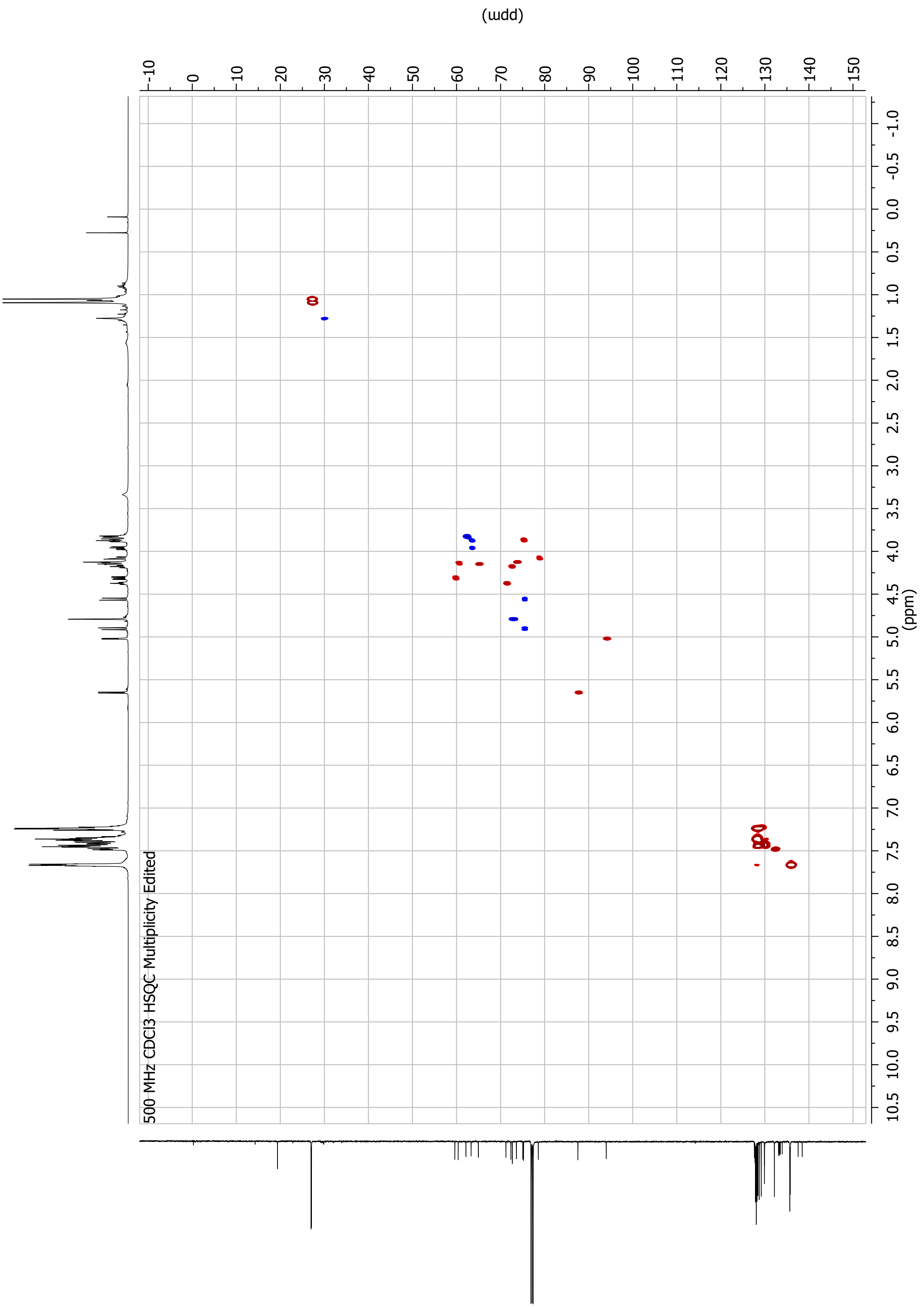

Figure S24: HSQC Multiplicity Edited NMR (500 MHz, $CDCl_3$)

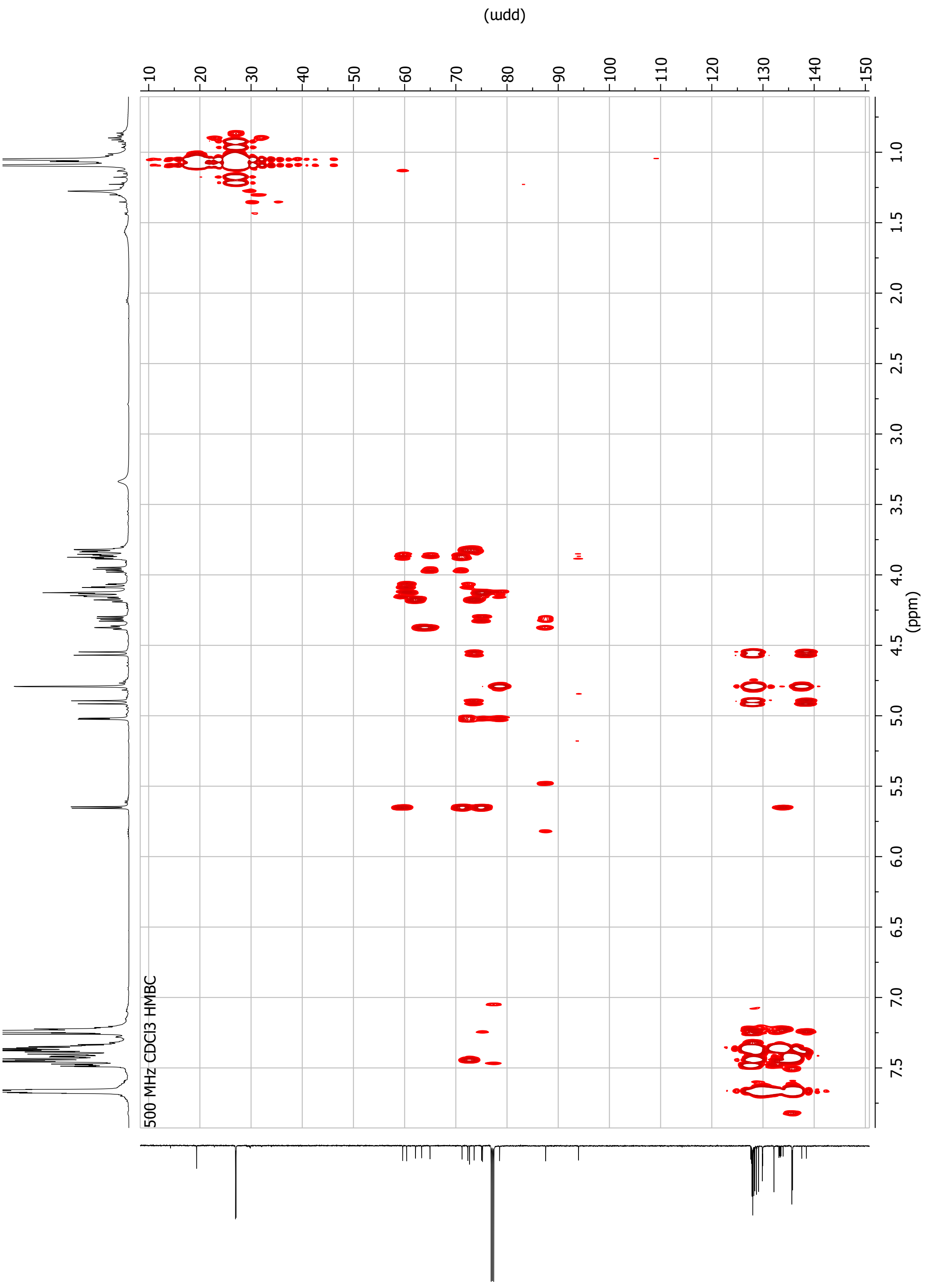

Figure S25: HMBC NMR (500 MHz, $CDCl_3$)

S2.7 Spectra for 19

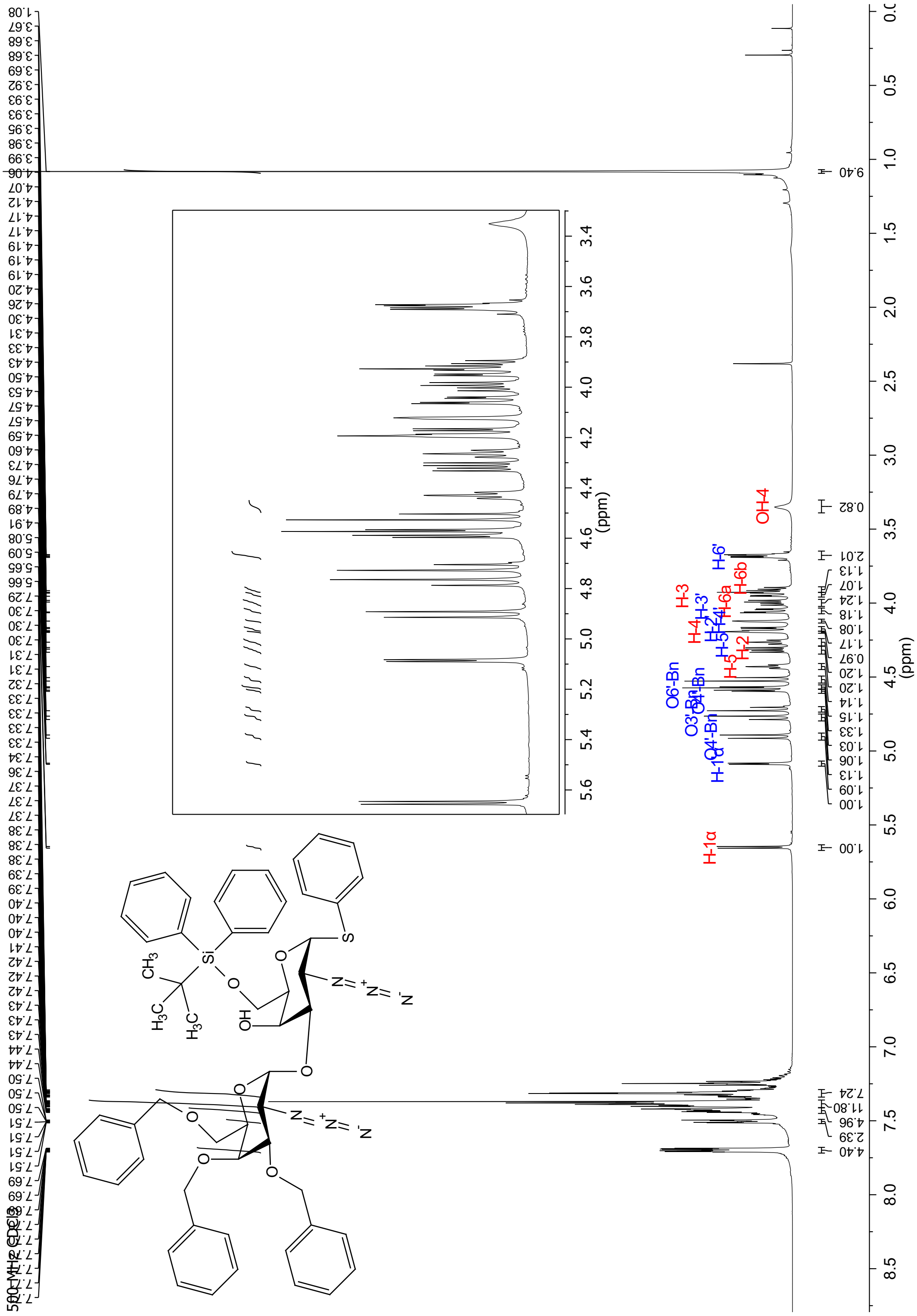

Figure S26: ^{1}H NMR (500 MHz, $CDCl_3$)

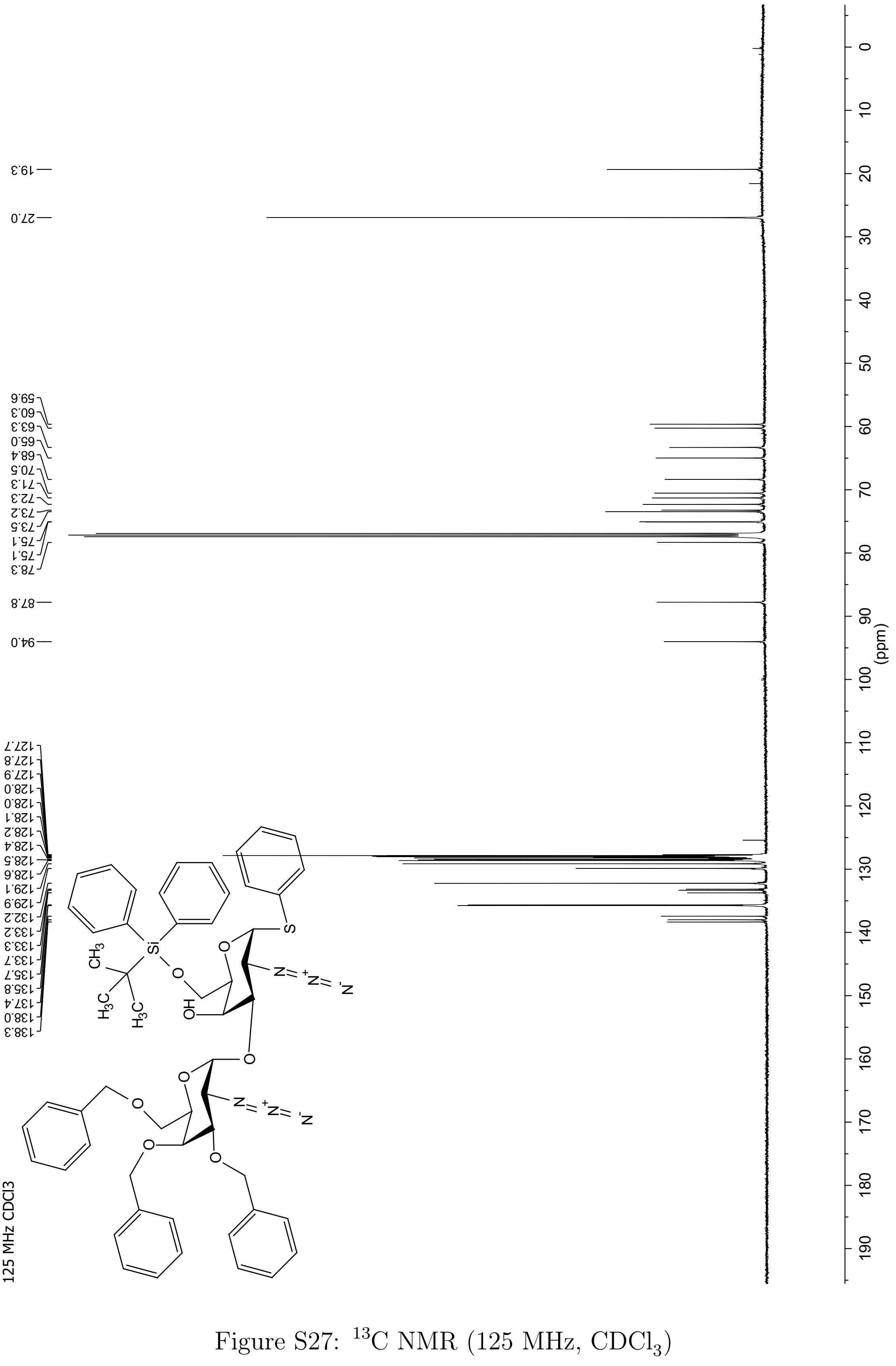

Figure S27: ^{13}C NMR (125 MHz, $CDCl_3$)

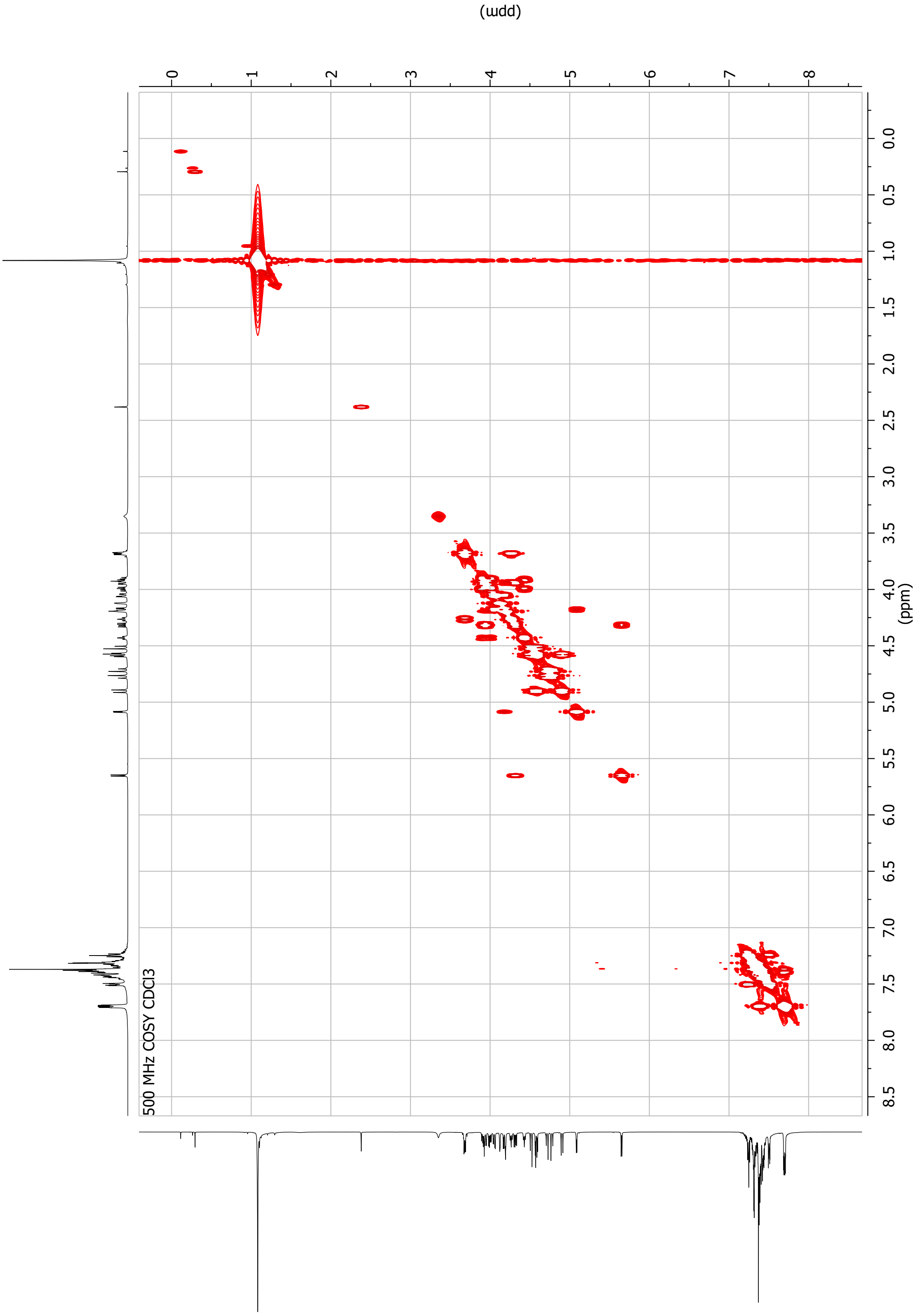

Figure S28: COSY NMR (500 MHz, $CDCl_3$)

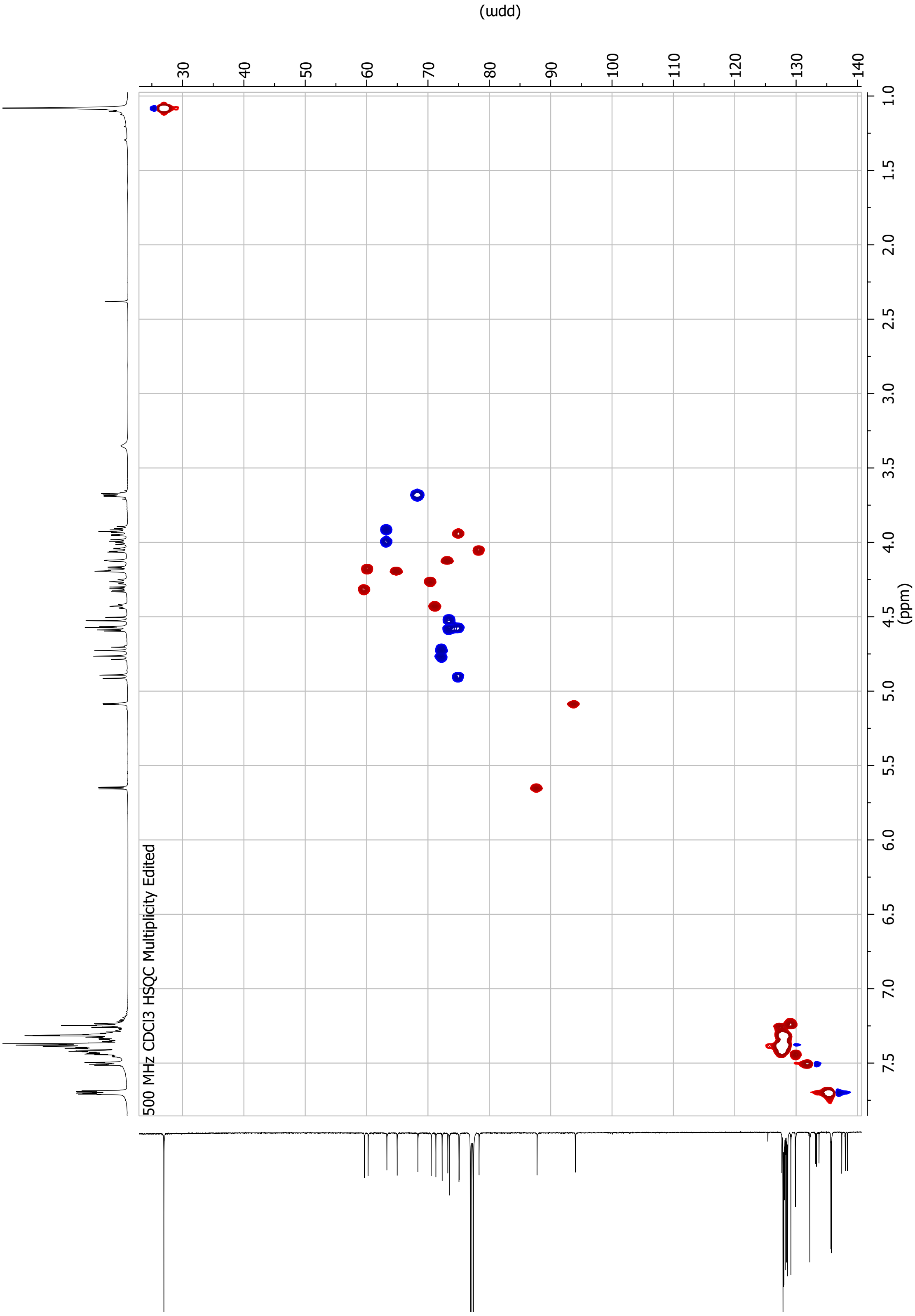

Figure S29: HSQC Multiplicity Edited NMR (500 MHz, $CDCl_3$)

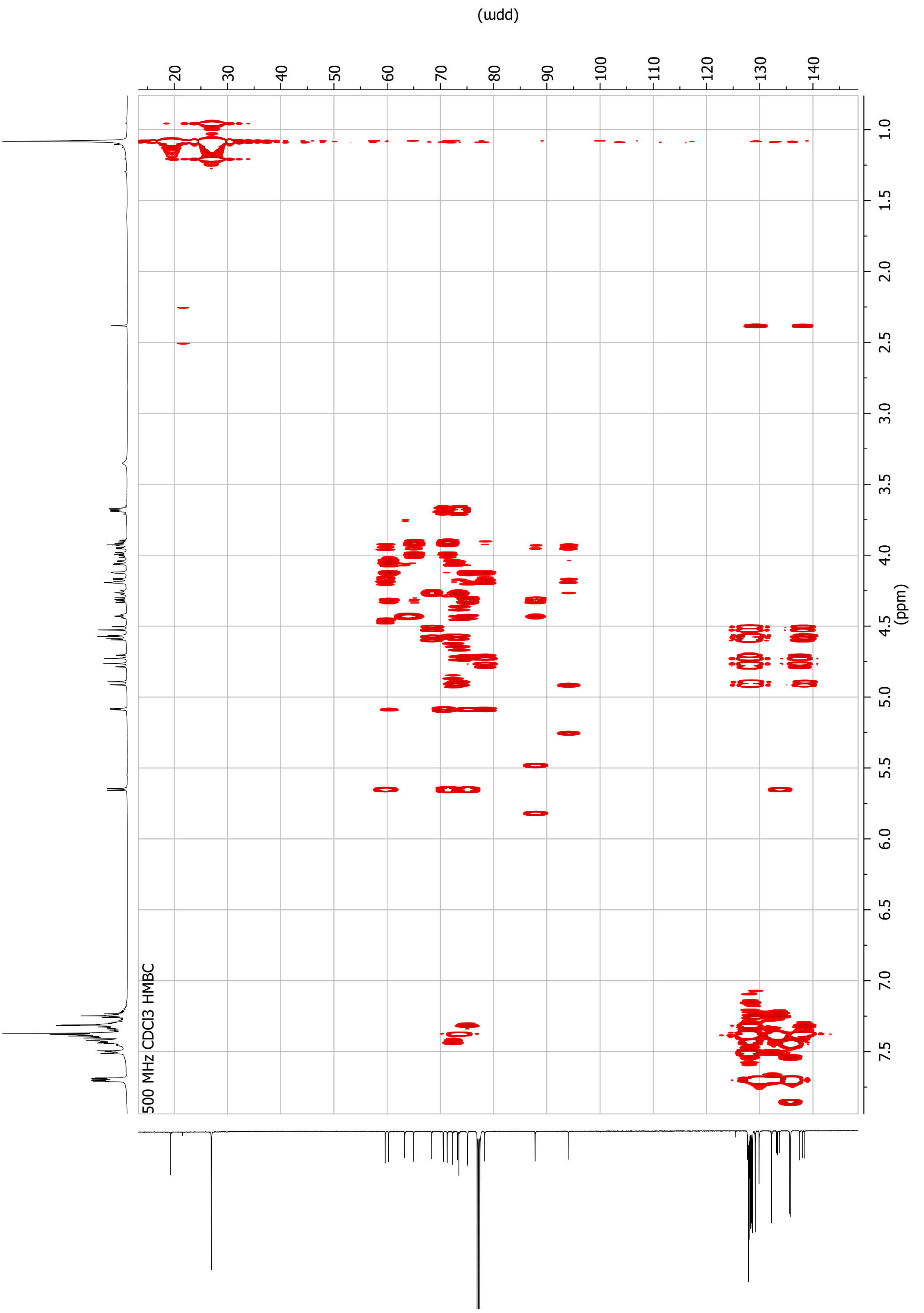

Figure S30: HMBC NMR (500 MHz, $CDCl_3$)

S2.8 Spectra for 20

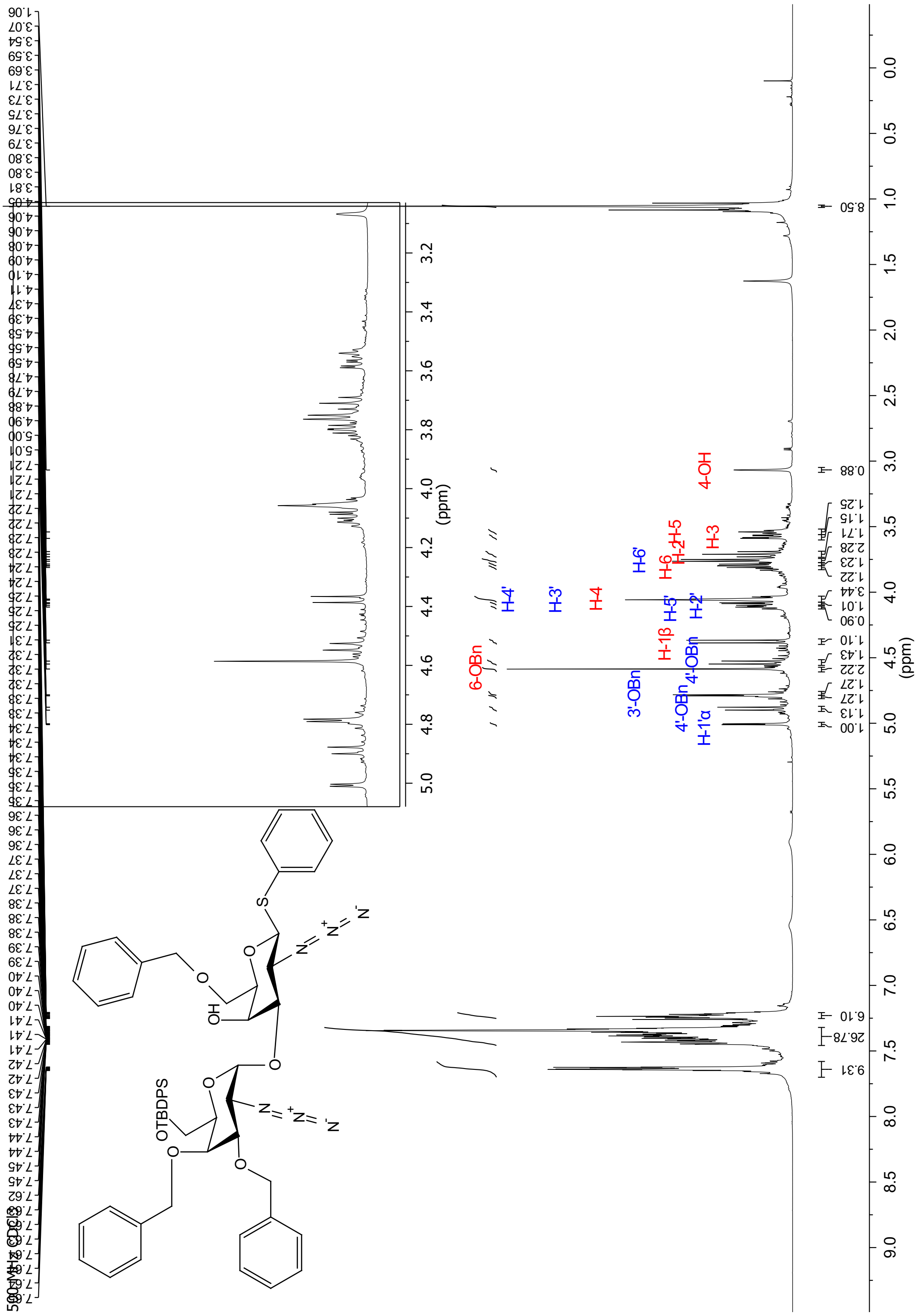

Figure S31: ^{1}H NMR (500 MHz, $CDCl_3$)

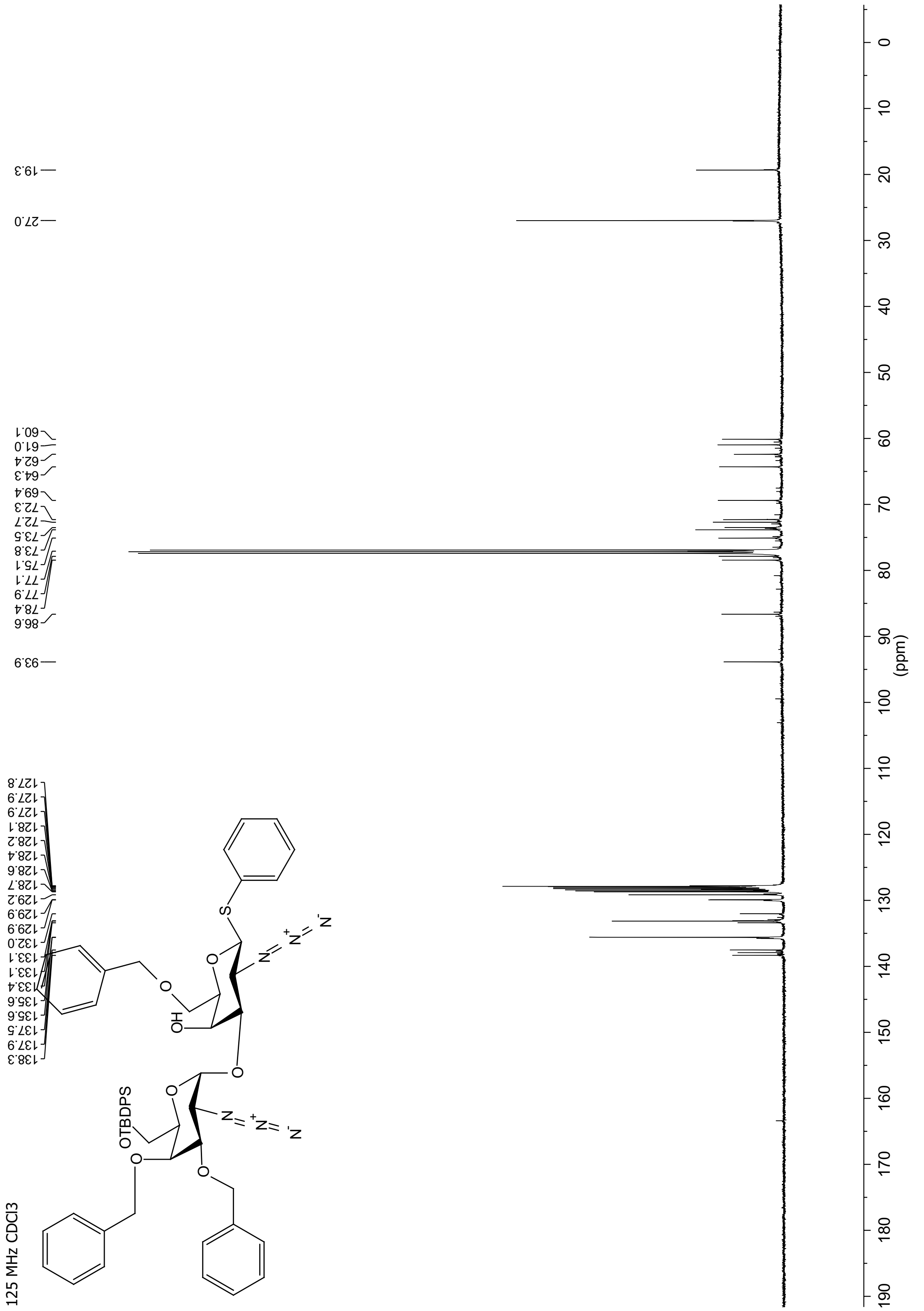

Figure S32: ^{13}C NMR (125 MHz, $CDCl_3$)

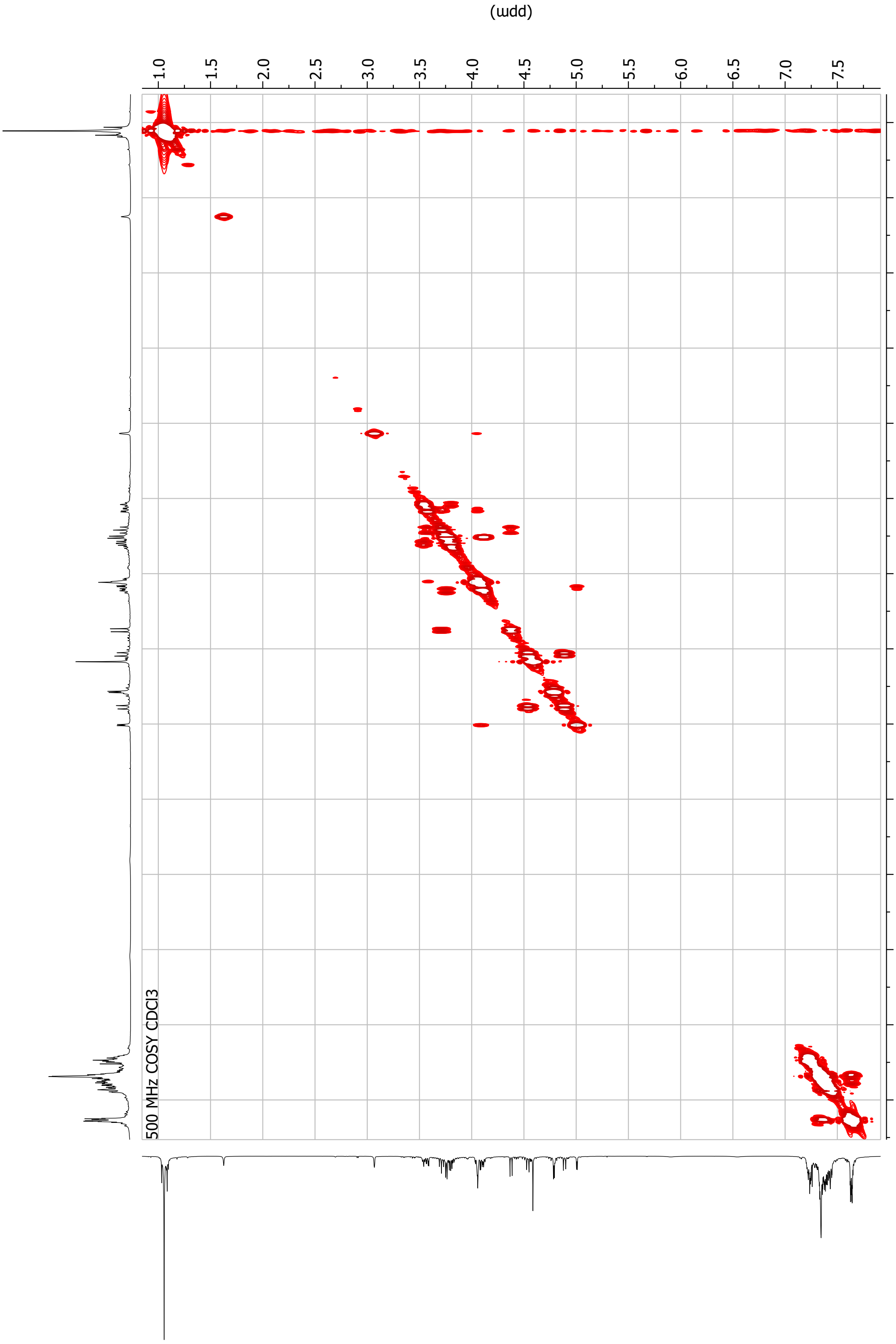

Figure S33: COSY NMR (500 MHz, $CDCl_3$)

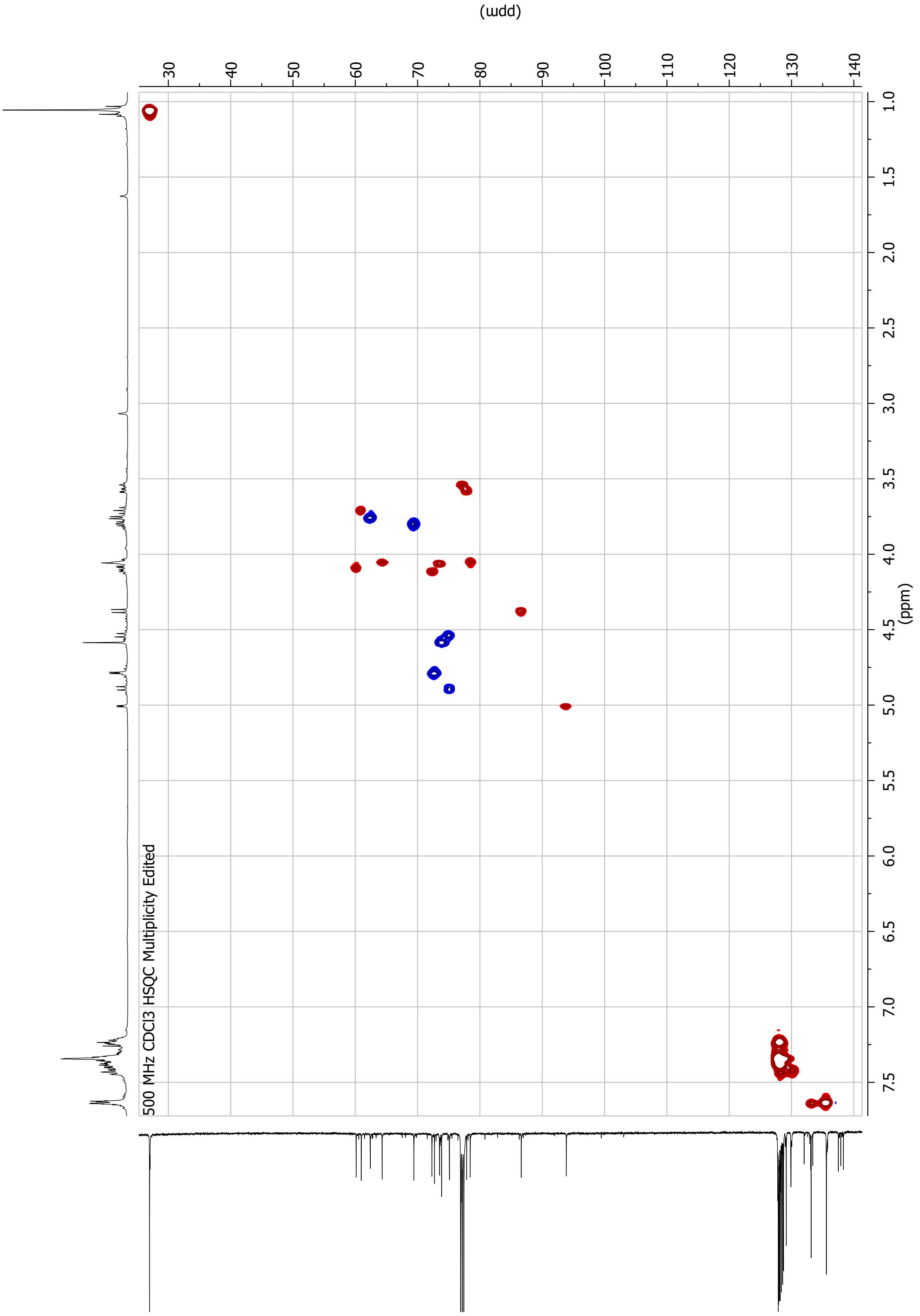

Figure S34: HSQC Multiplicity Edited NMR (500 MHz, $CDCl_3$)

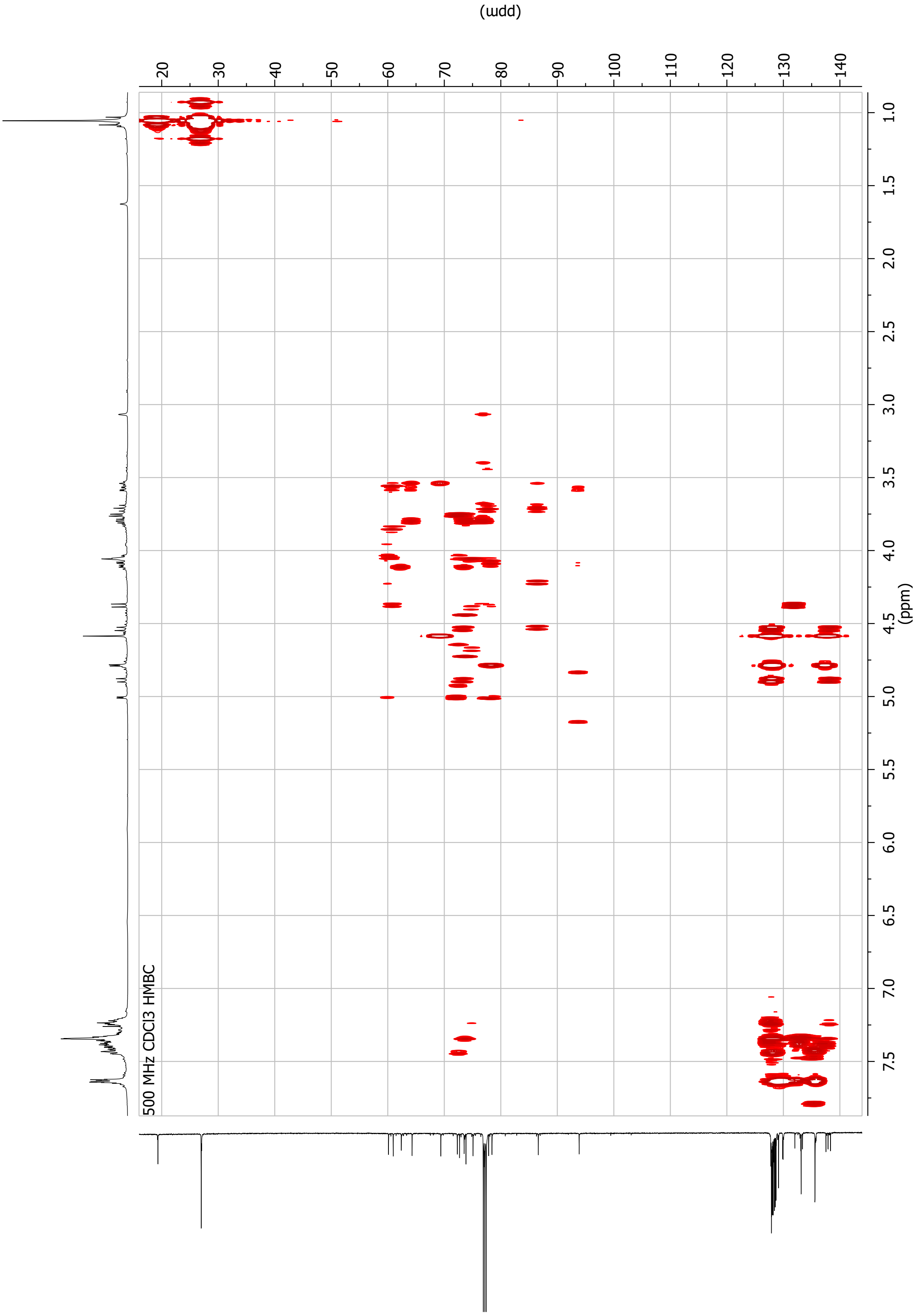

Figure S35: HMBC NMR (500 MHz, $CDCl_3$)

S2.9 Spectra for 26

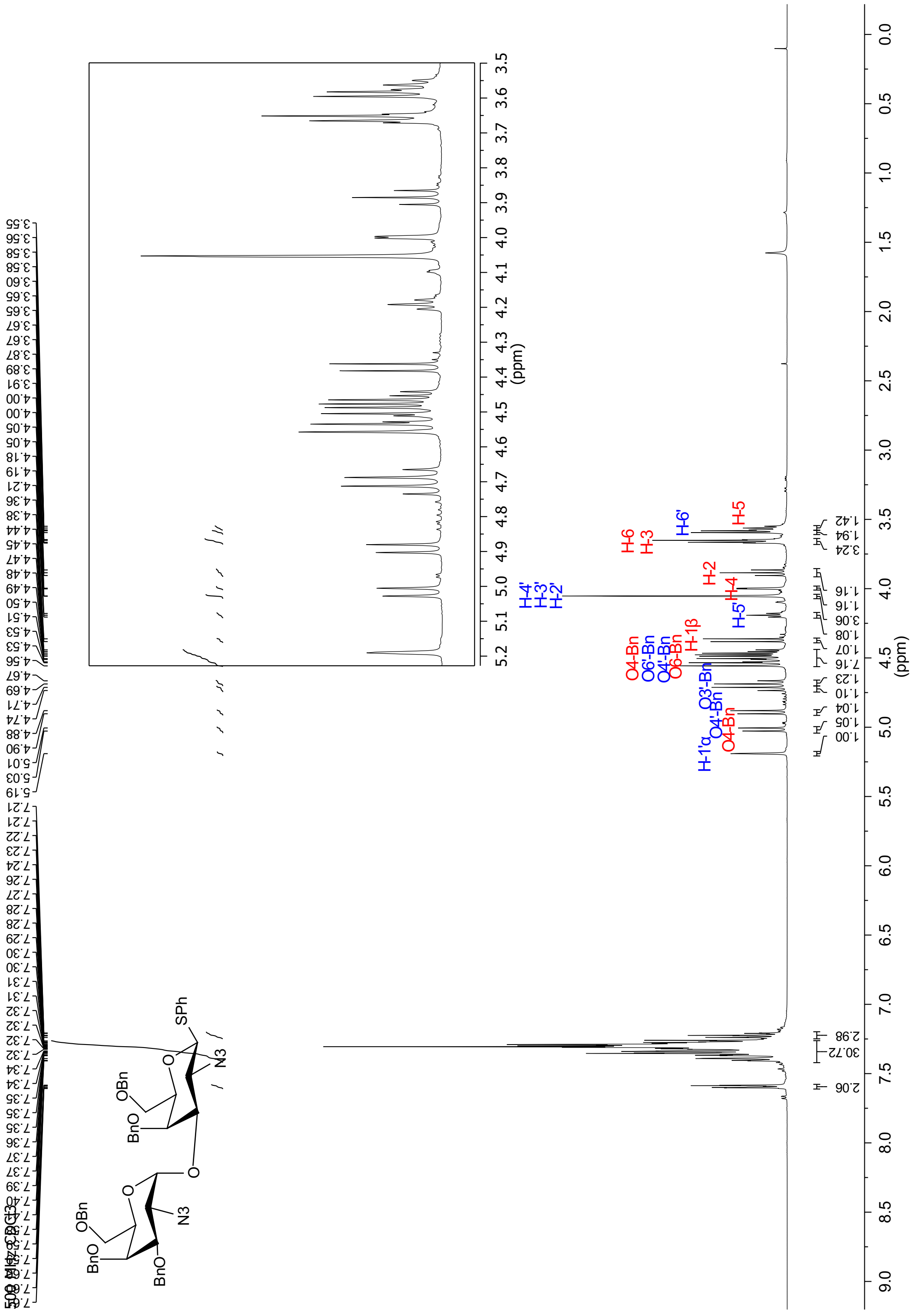

Figure S36: ^{1}H NMR (500 MHz, $CDCl_3$)

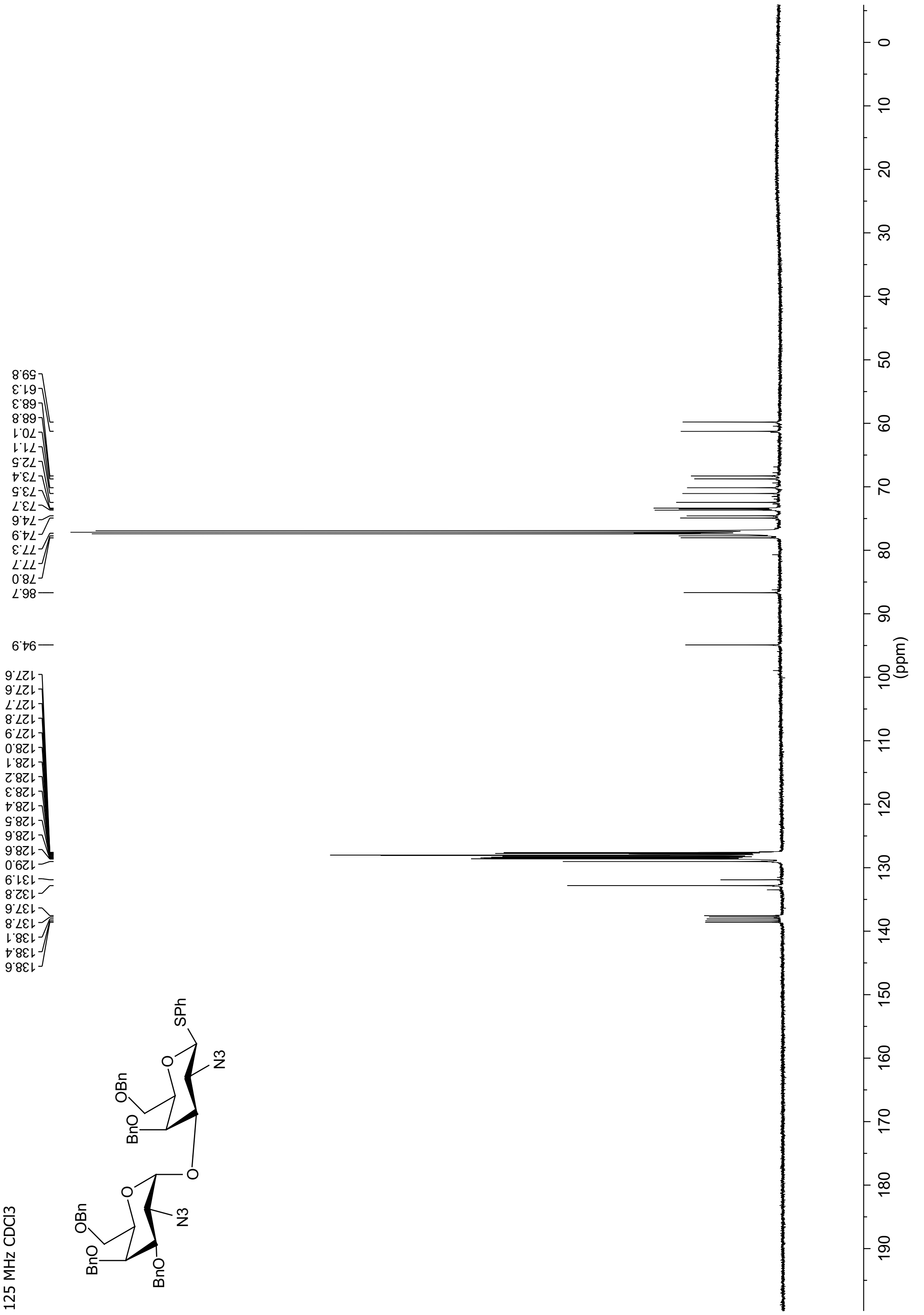

Figure S37: ^{13}C NMR (125 MHz, $CDCl_3$)

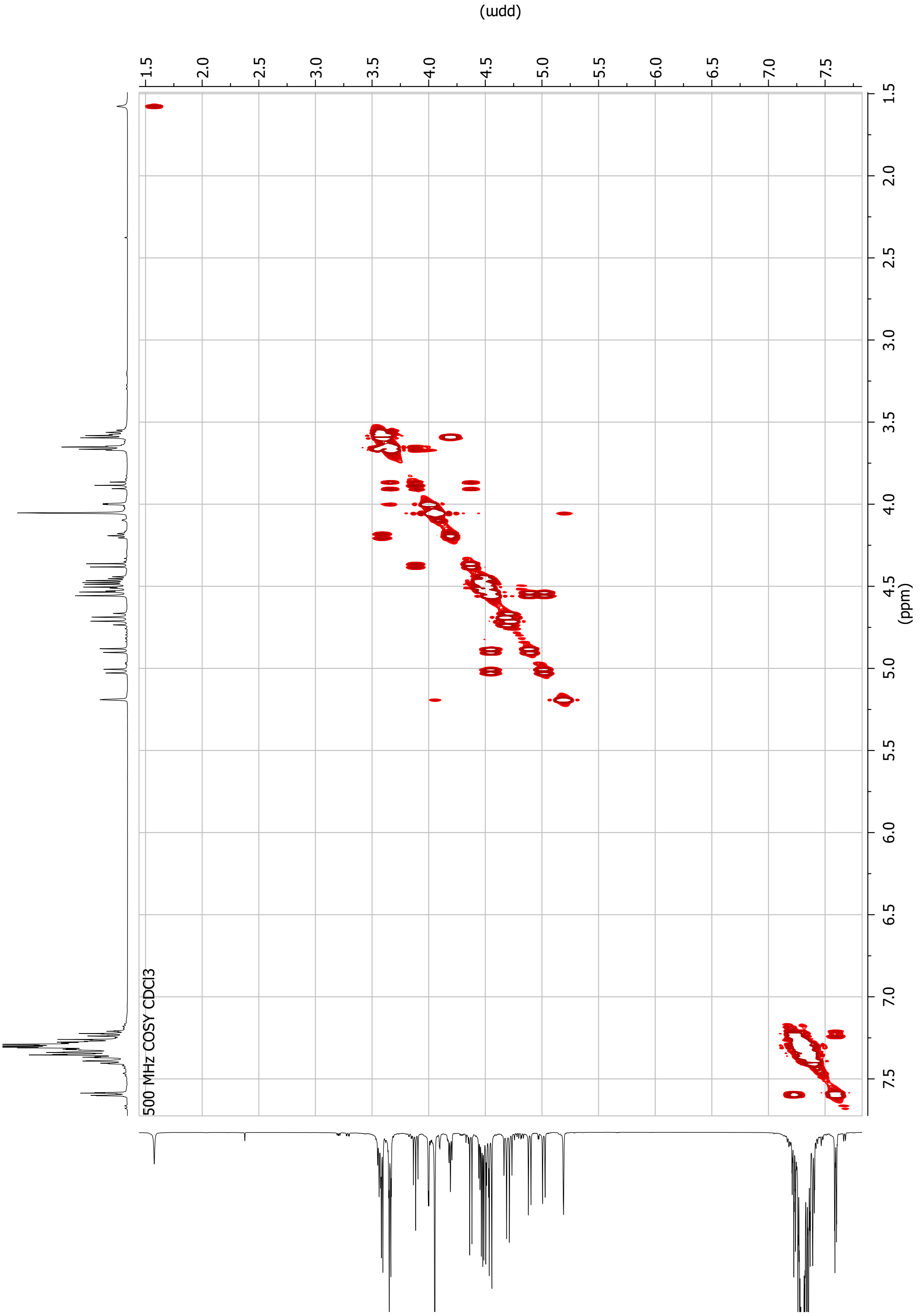

Figure S38: COSY NMR (500 MHz, $CDCl_3$)

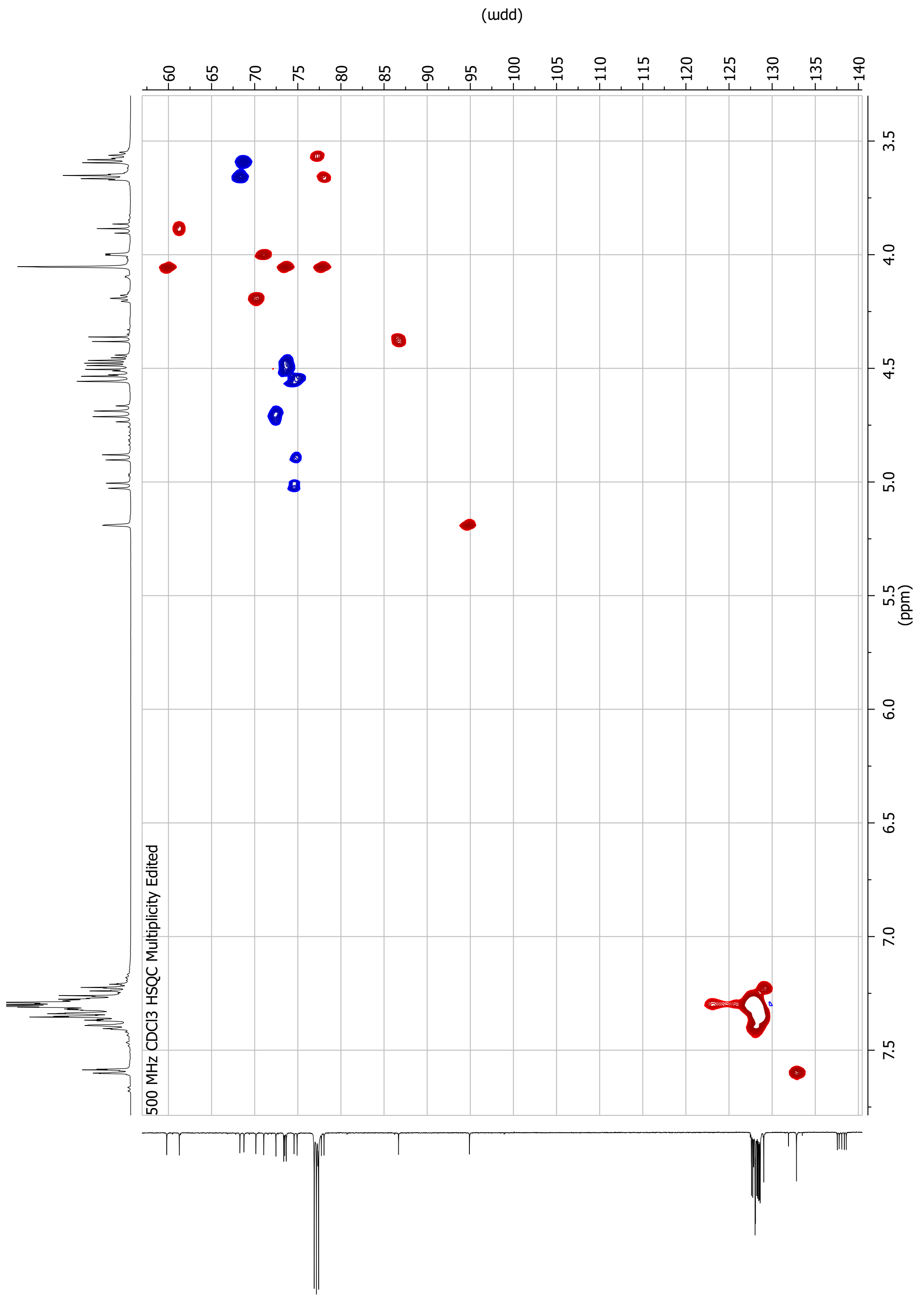

Figure S39: HSQC Multiplicity Edited NMR (500 MHz, $CDCl_3$)

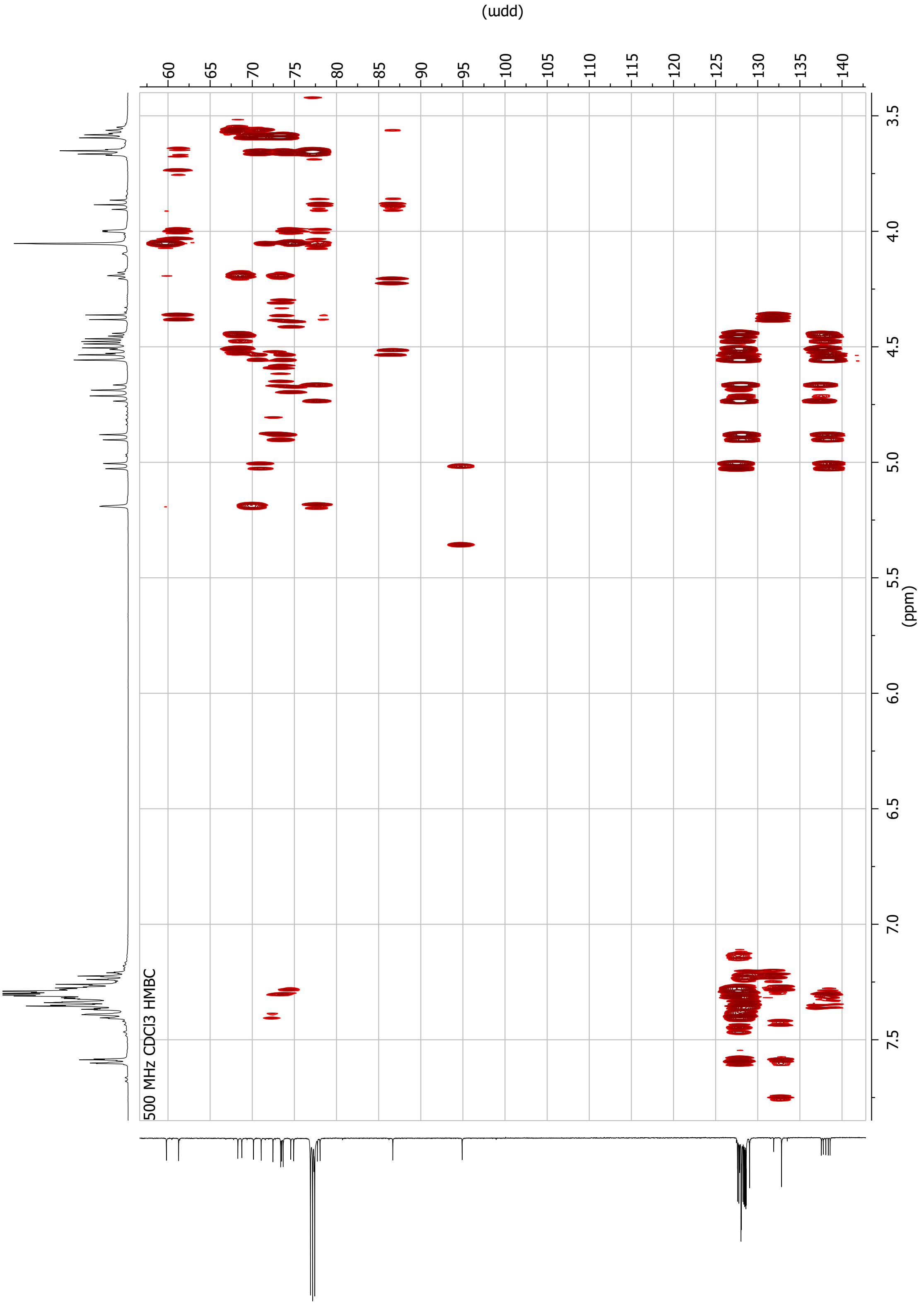

Figure S40: HMBC NMR (500 MHz, $CDCl_3$)

S2.10 Spectra for 22

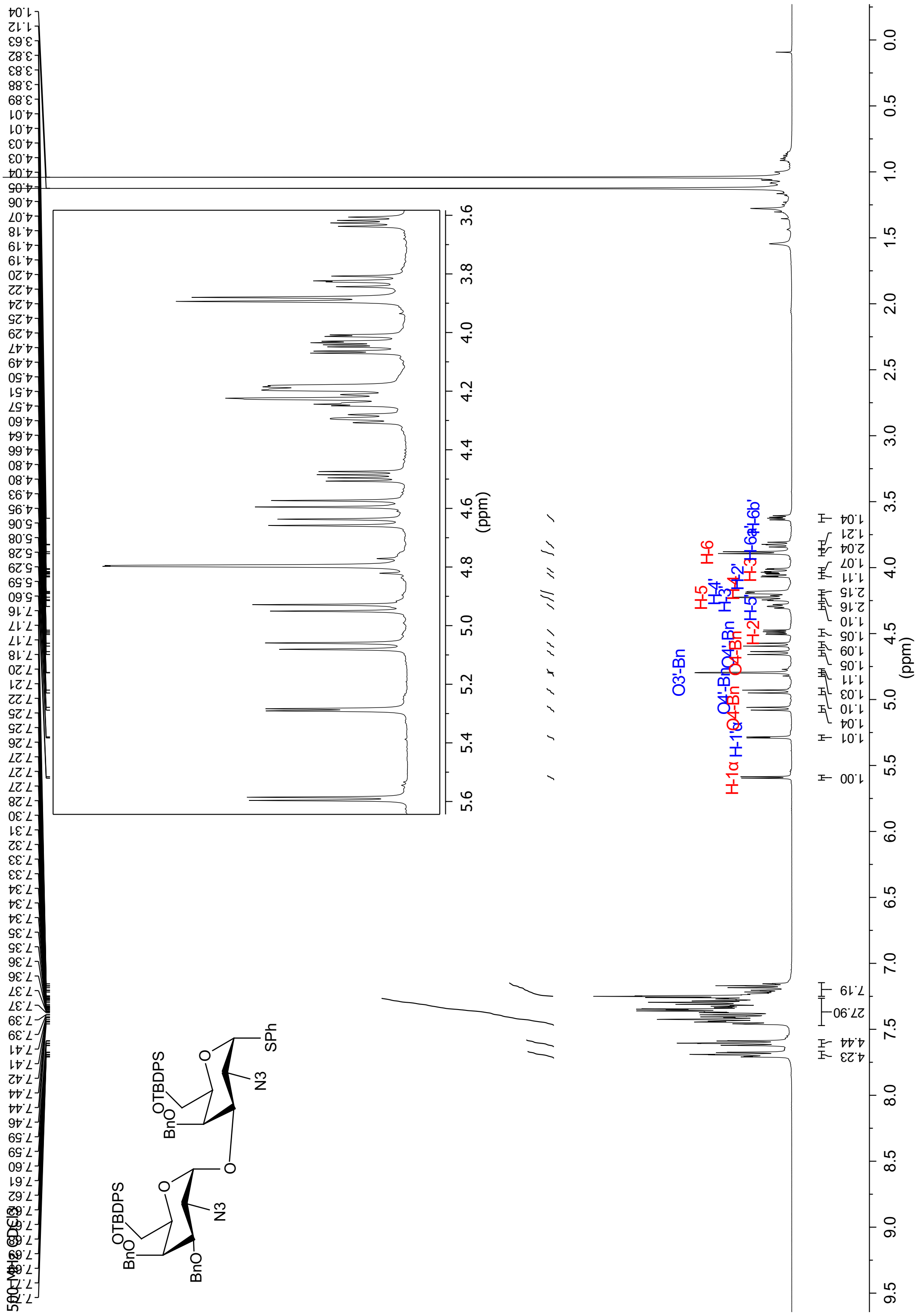

Figure S41: ^{1}H NMR (500 MHz, $CDCl_3$)

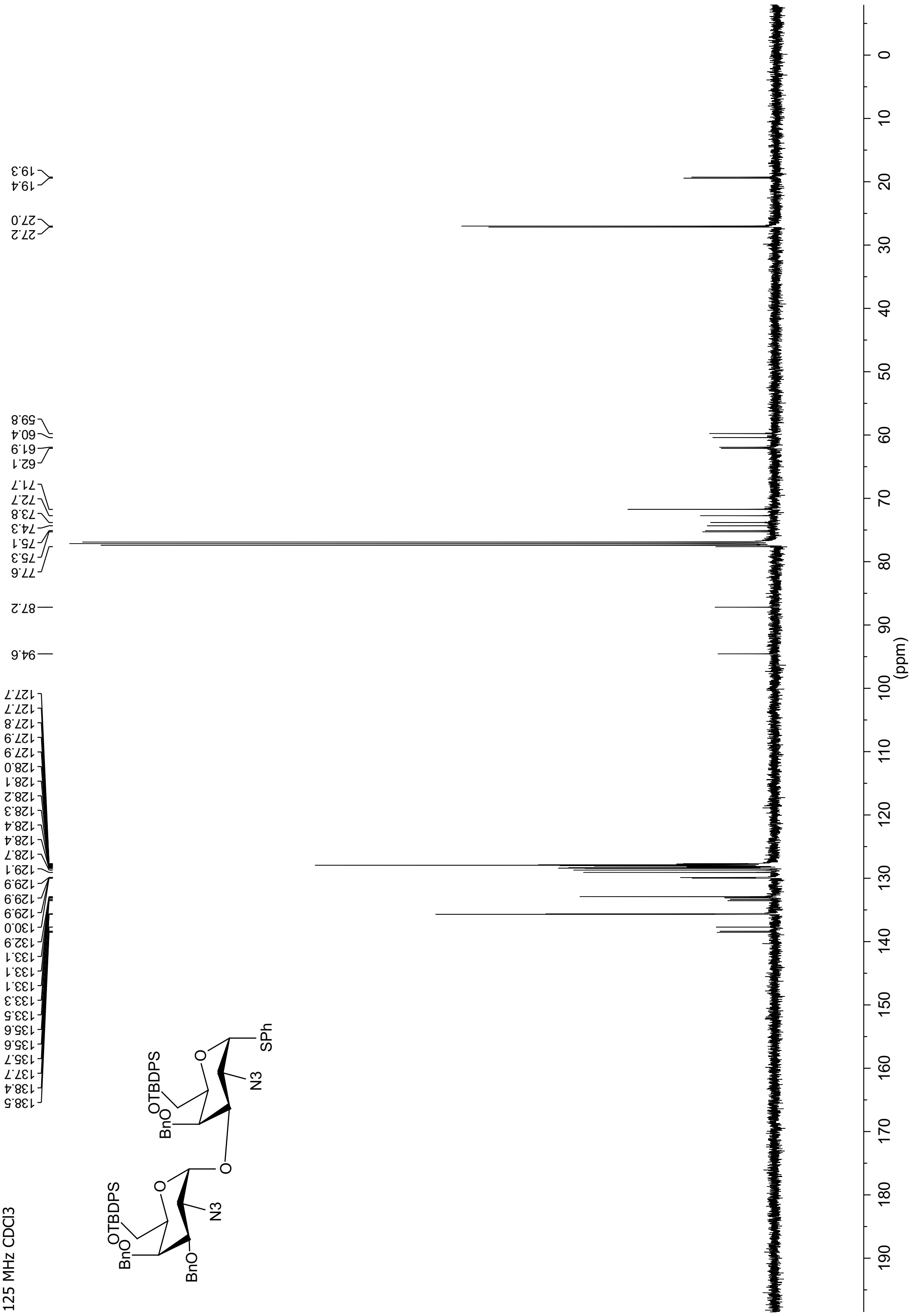

Figure S42: ^{13}C NMR (125 MHz, $CDCl_3$)

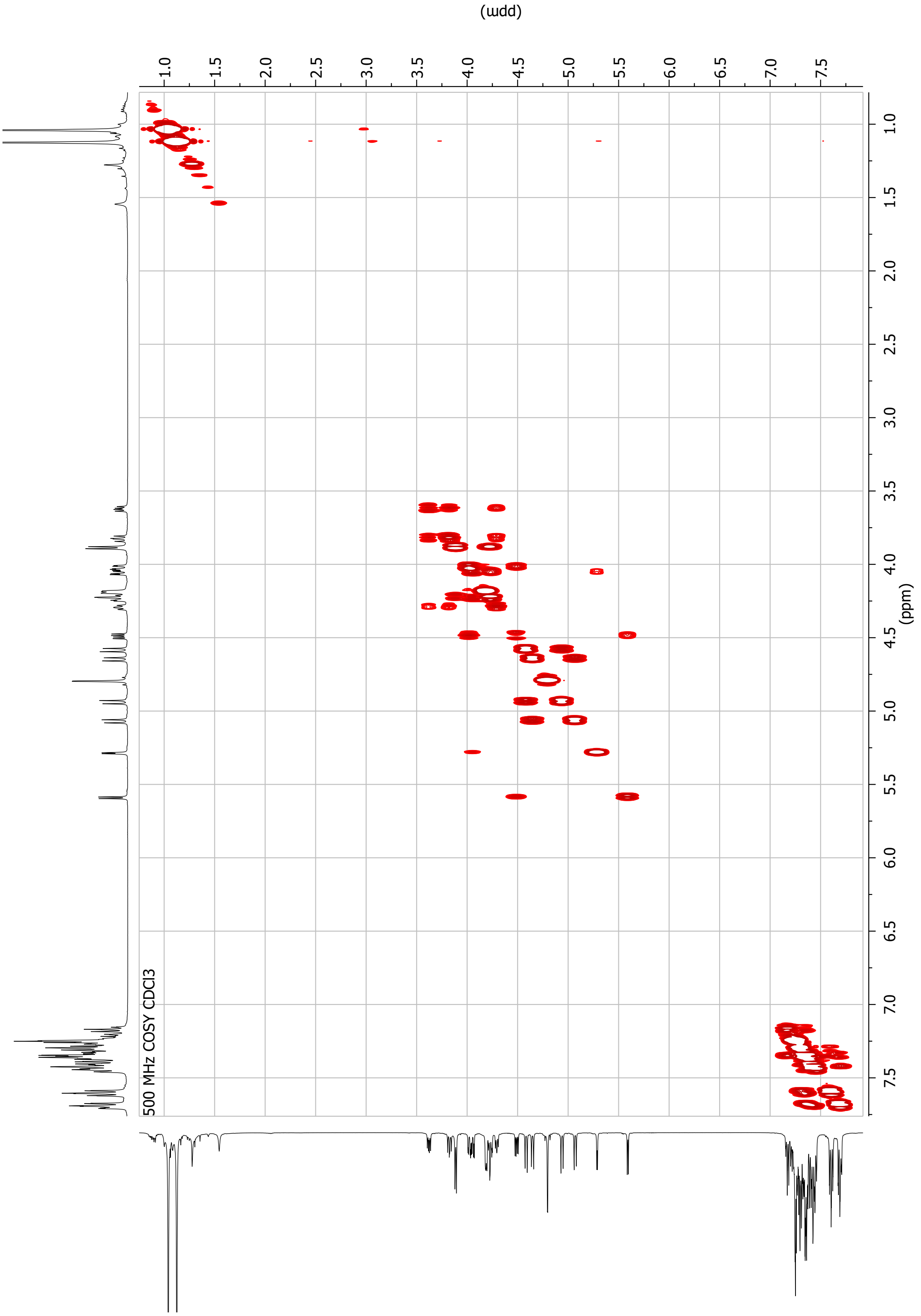

Figure S43: COSY NMR (500 MHz, $CDCl_3$)

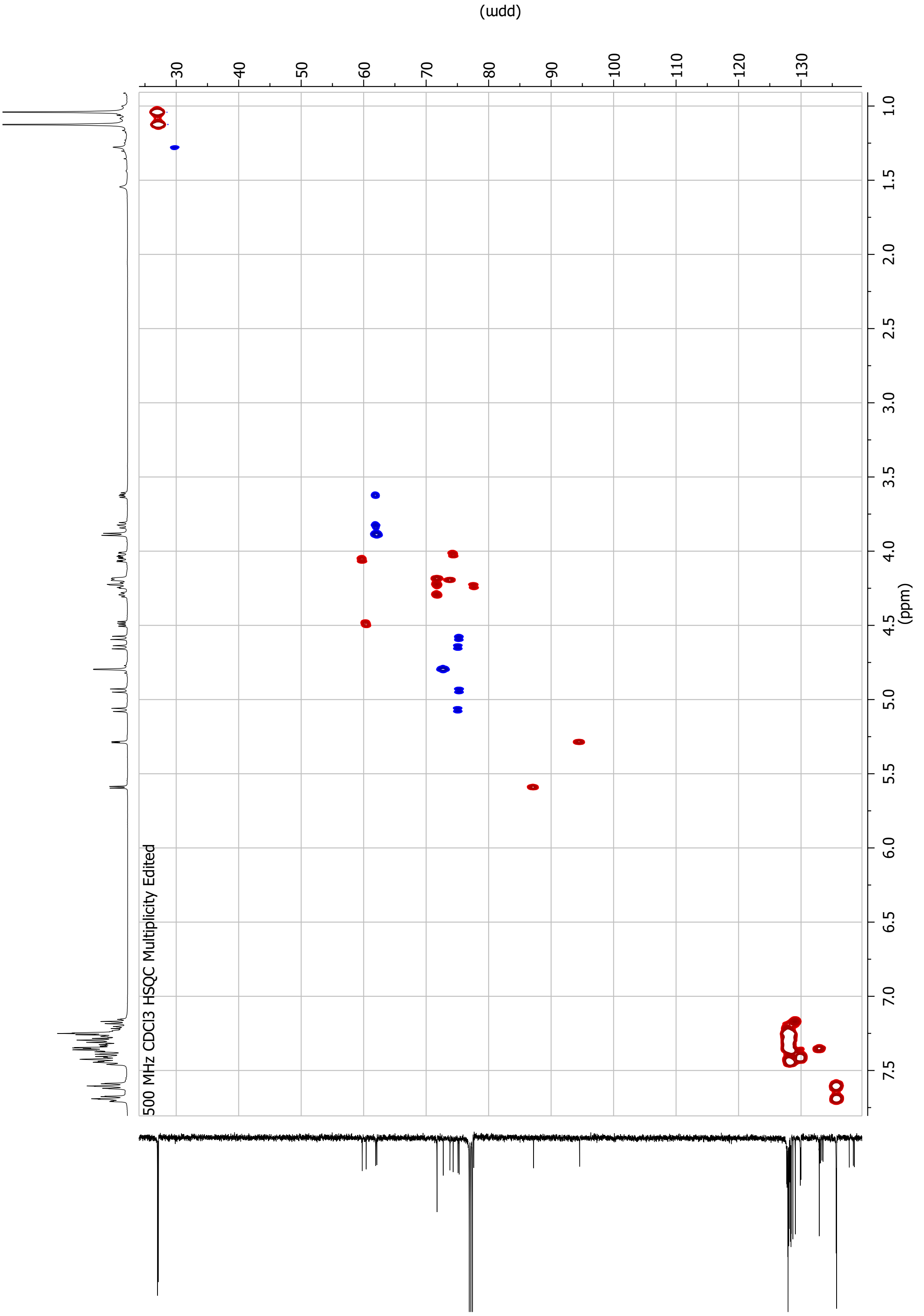

Figure S44: HSQC Multiplicity Edited NMR (500 MHz, $CDCl_3$)

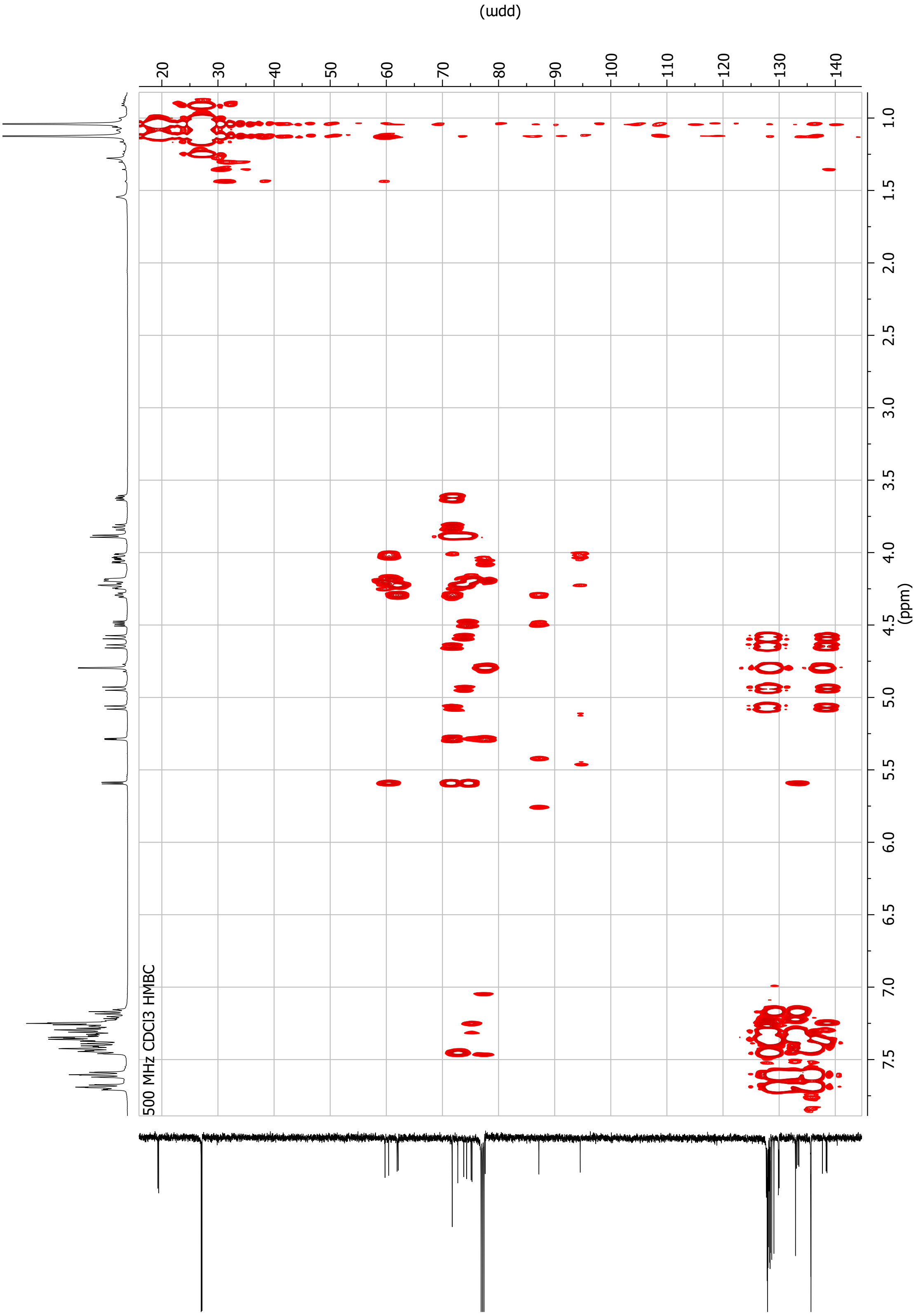

Figure S45: HMBC NMR (500 MHz, $CDCl_3$)

S2.11 Spectra for 23

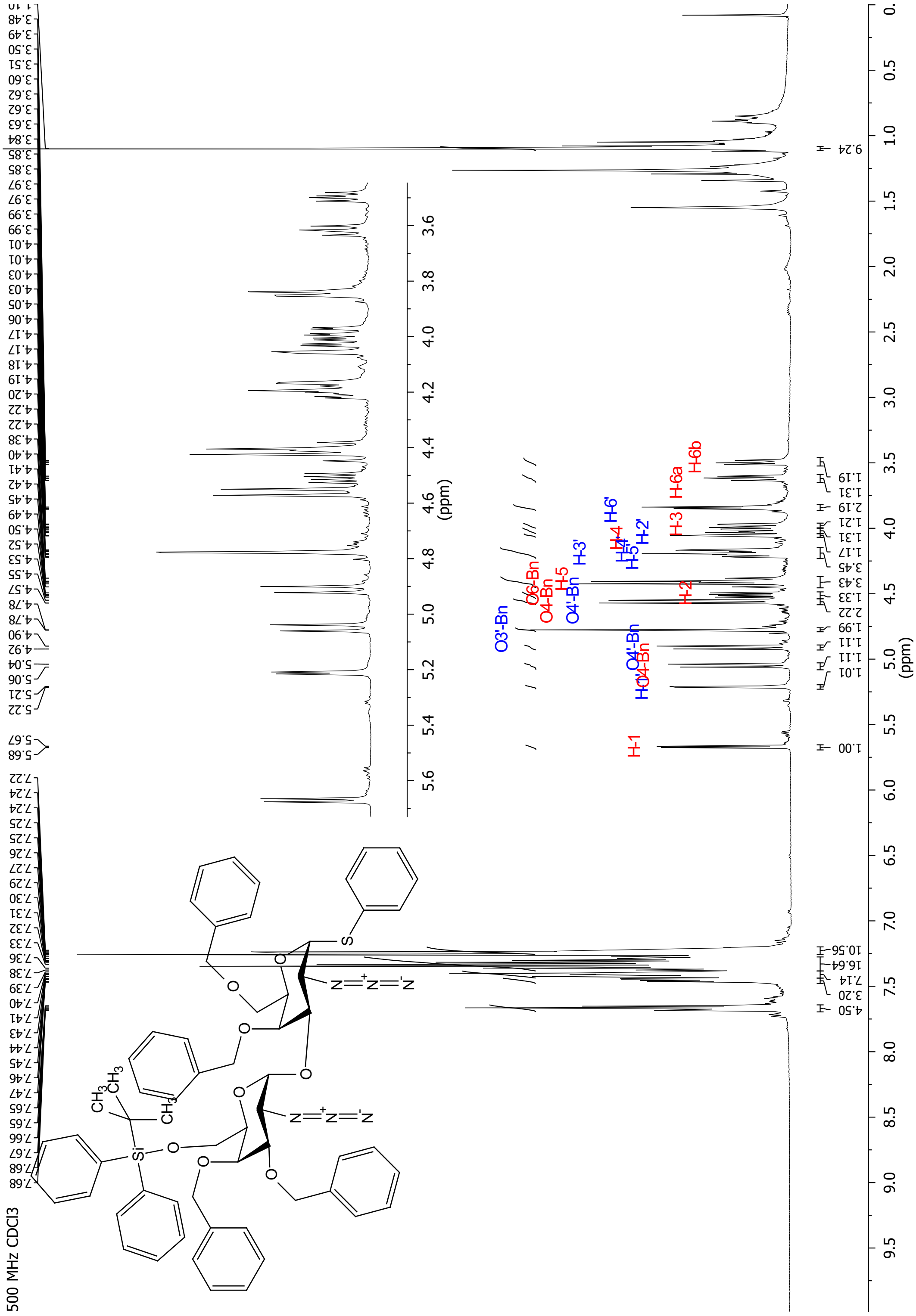

Figure S46: ^{1}H NMR (500 MHz, $CDCl_3$)

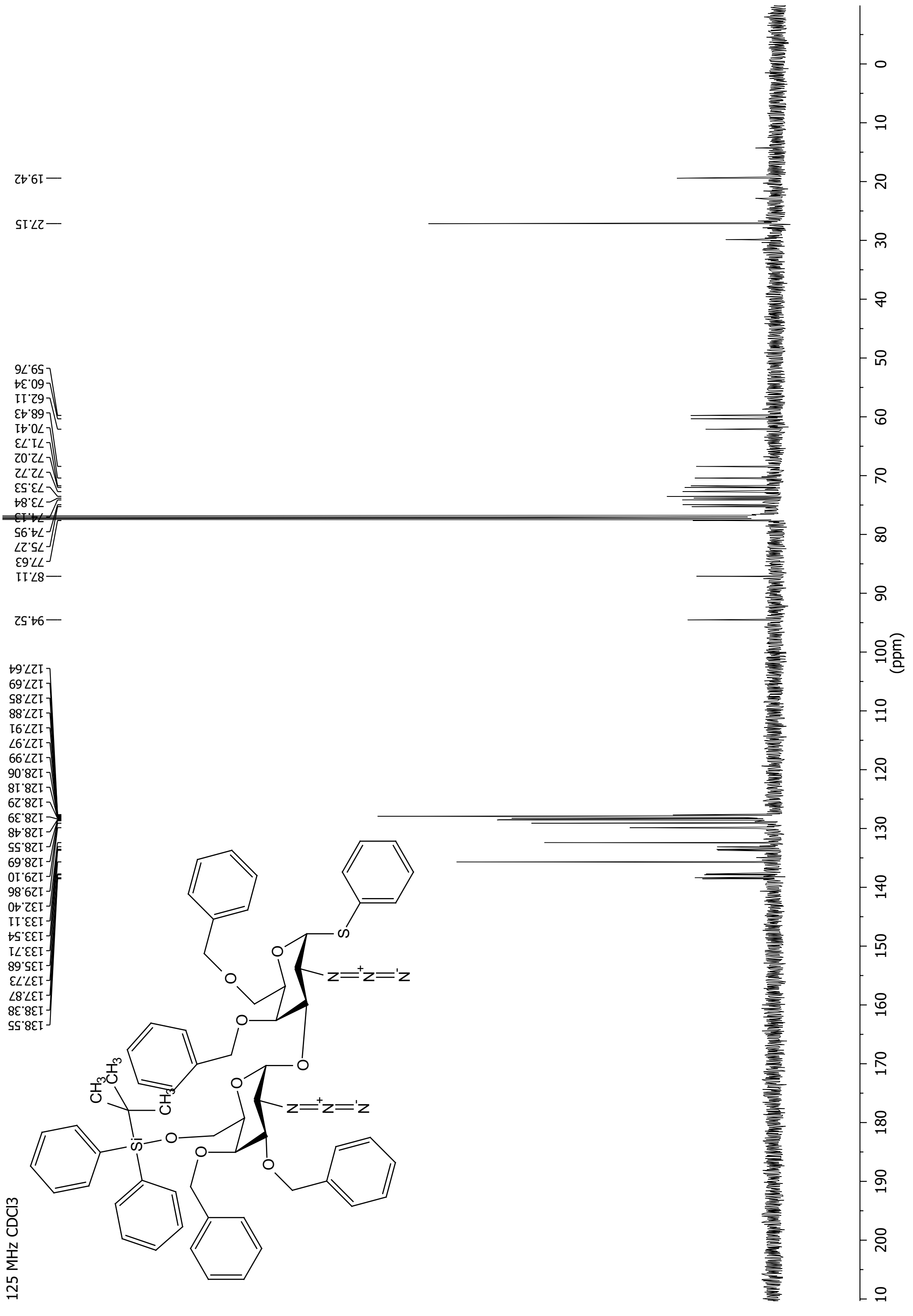

Figure S47: ^{13}C NMR (125 MHz, $CDCl_3$)

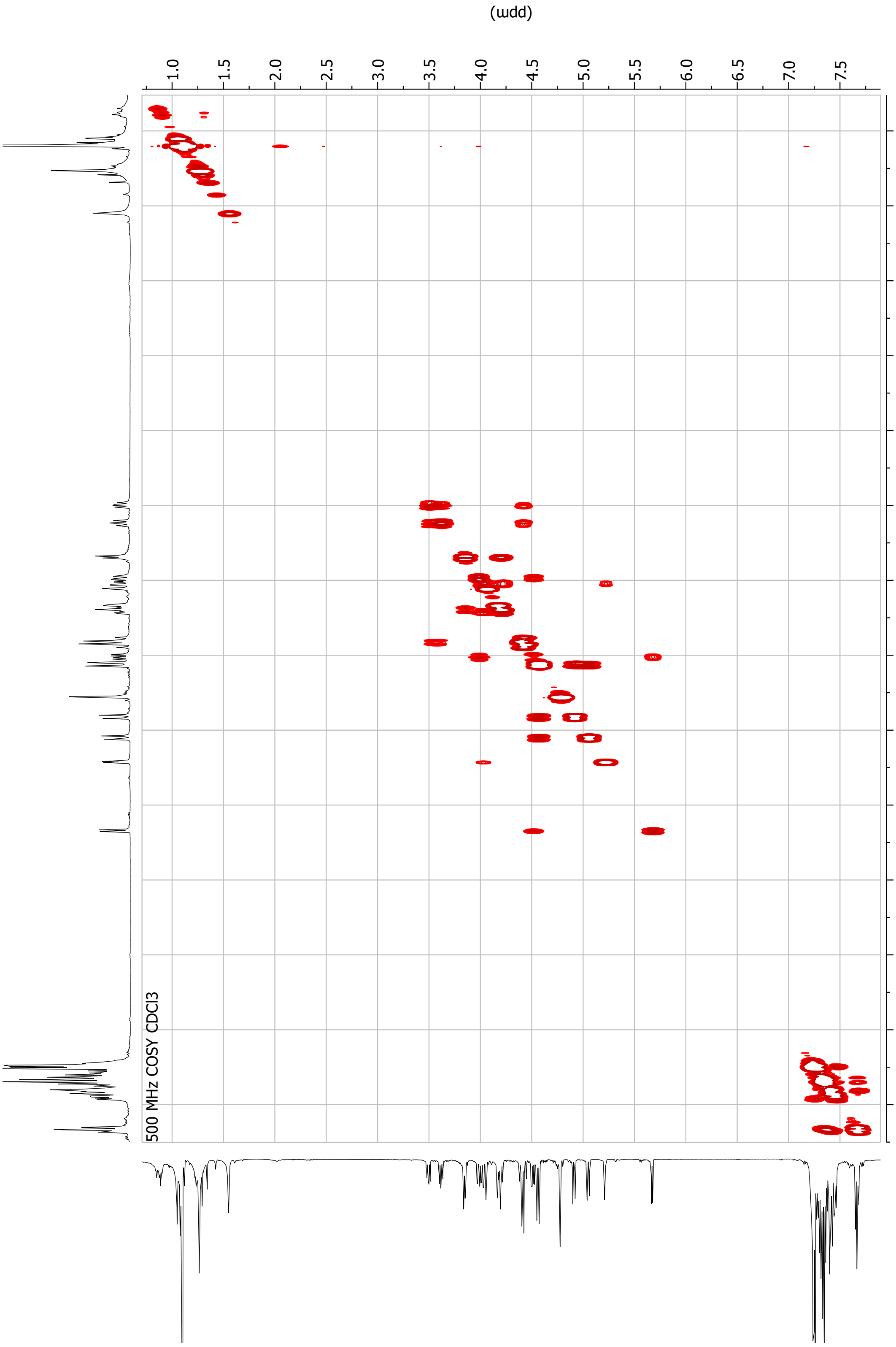

Figure S48: COSY NMR (500 MHz, $CDCl_3$)

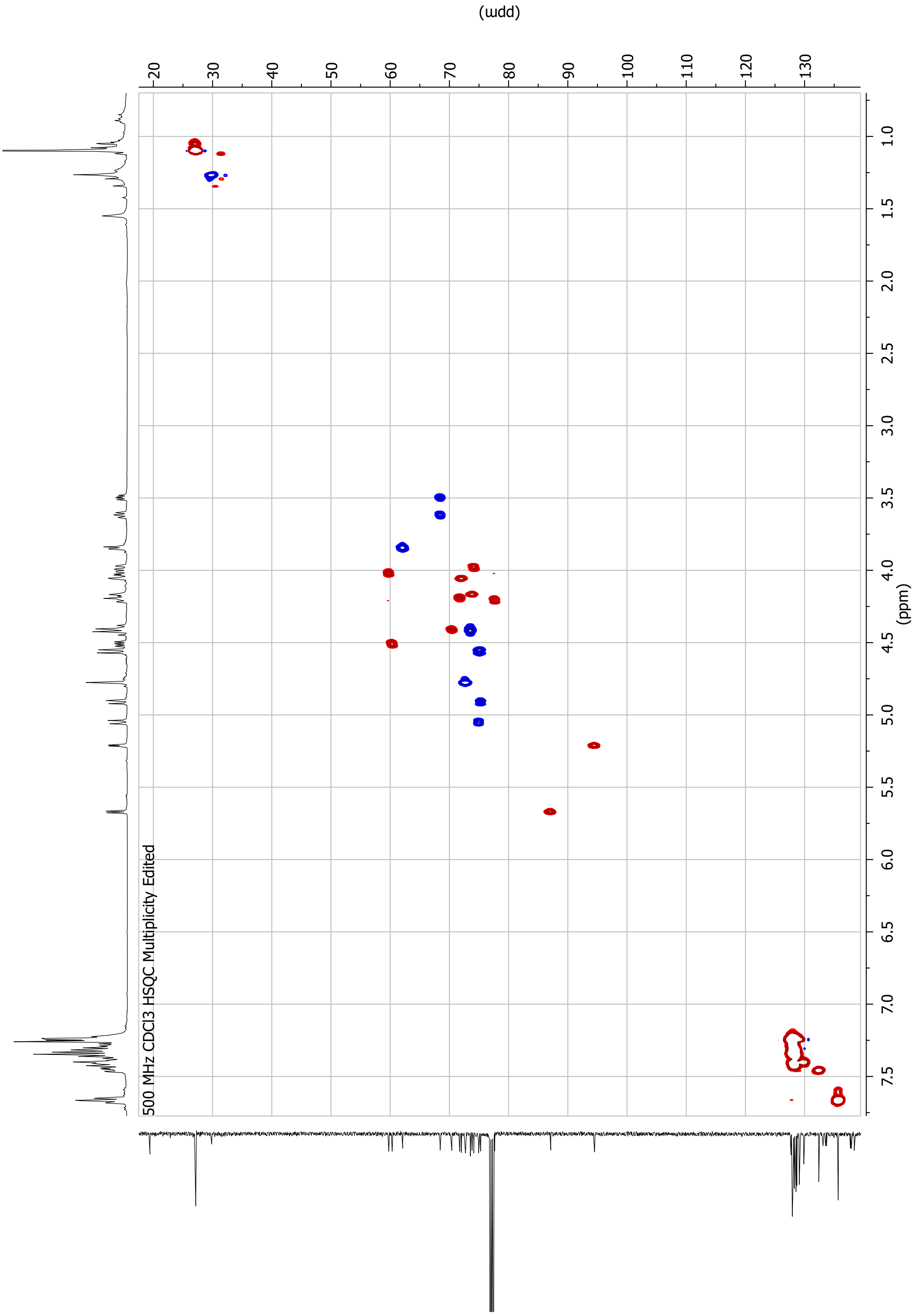

Figure S49: HSQC Multiplicity Edited NMR (500 MHz, $CDCl_3$)

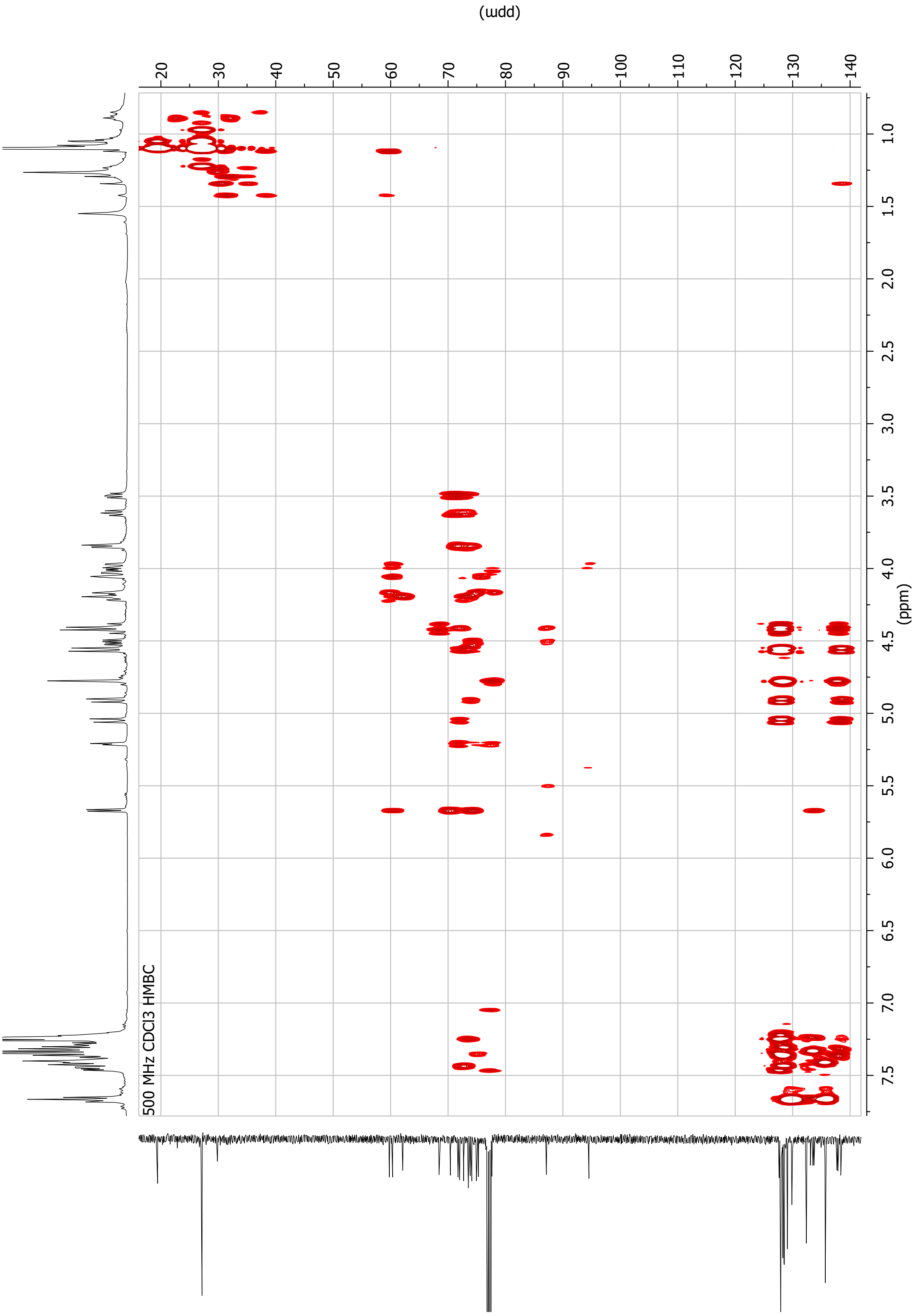

Figure S50: HMBC NMR (500 MHz, $CDCl_3$)

S2.12 Spectra for 24

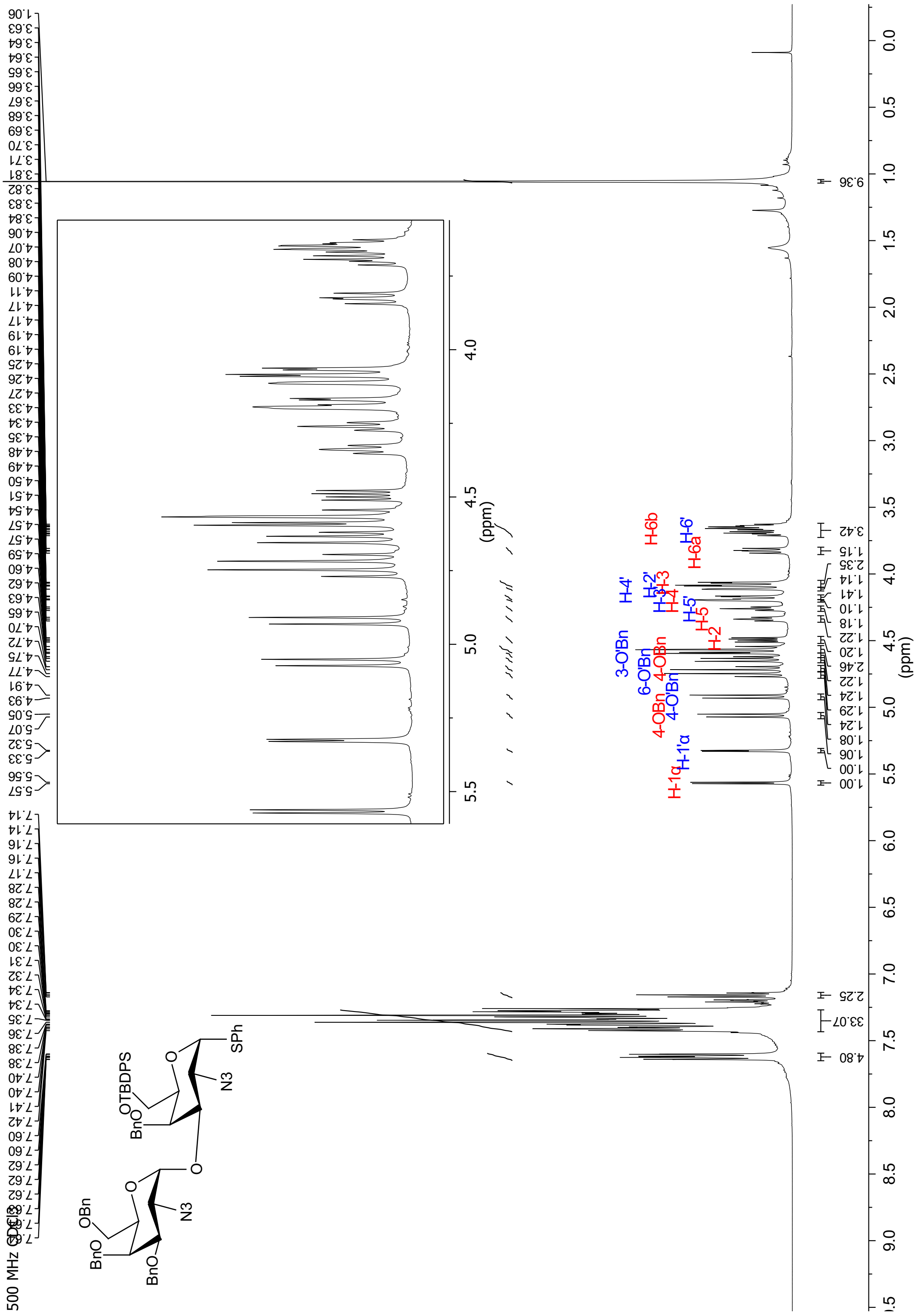

Figure S51: ^{1}H NMR (500 MHz, $CDCl_3$)

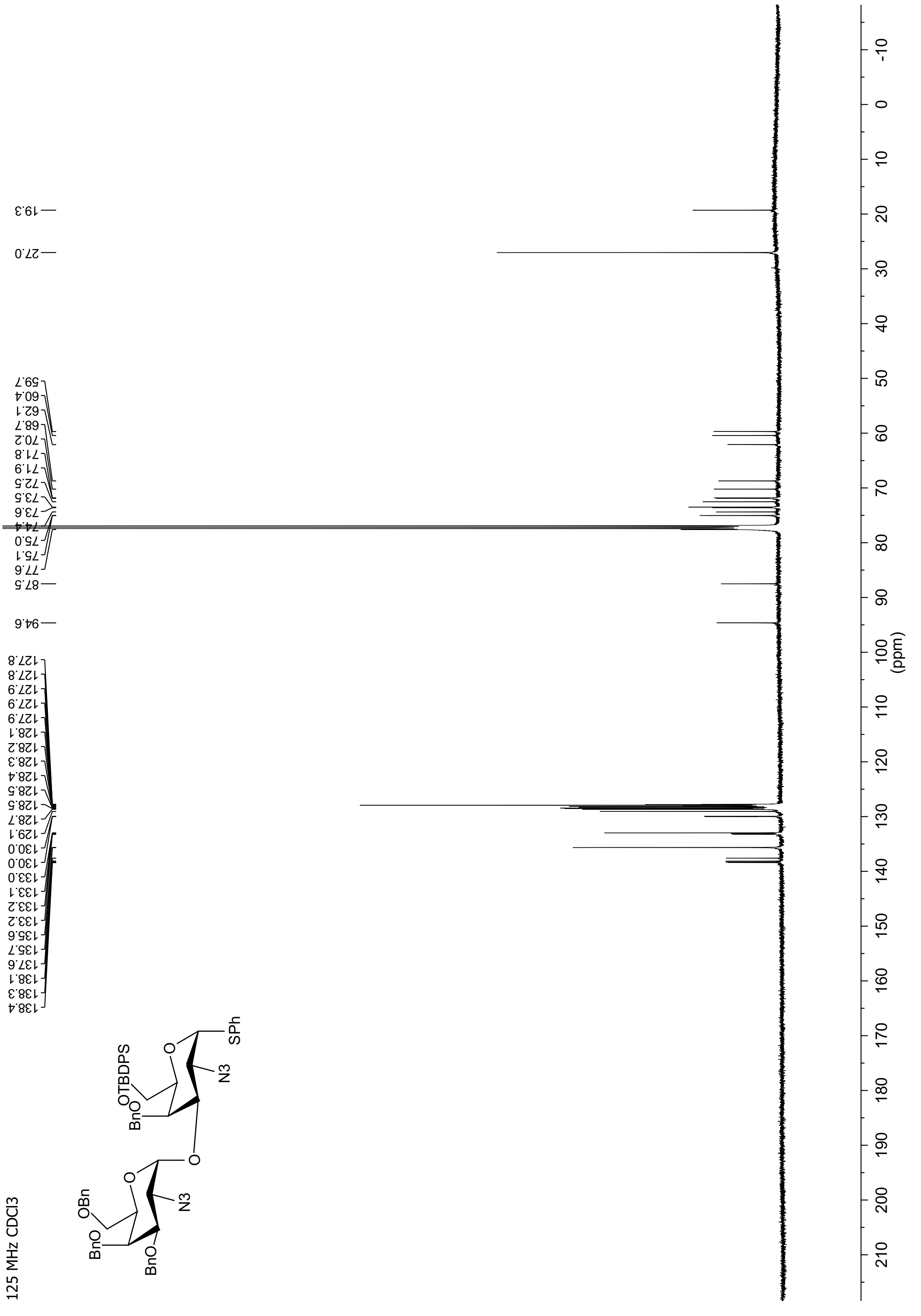

Figure S52: ^{13}C NMR (125 MHz, $CDCl_3$)

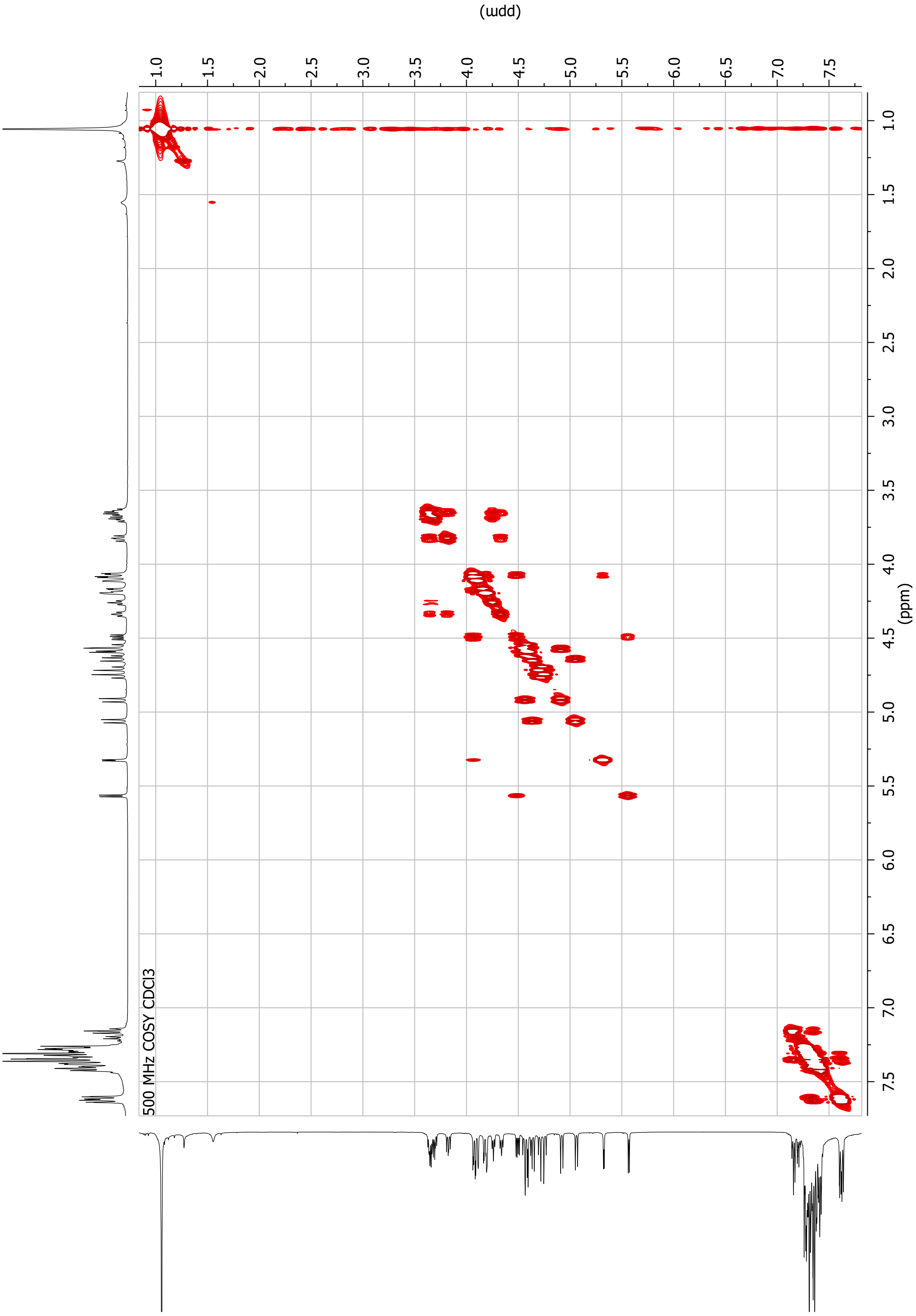

Figure S53: COSY NMR (500 MHz, $CDCl_3$)

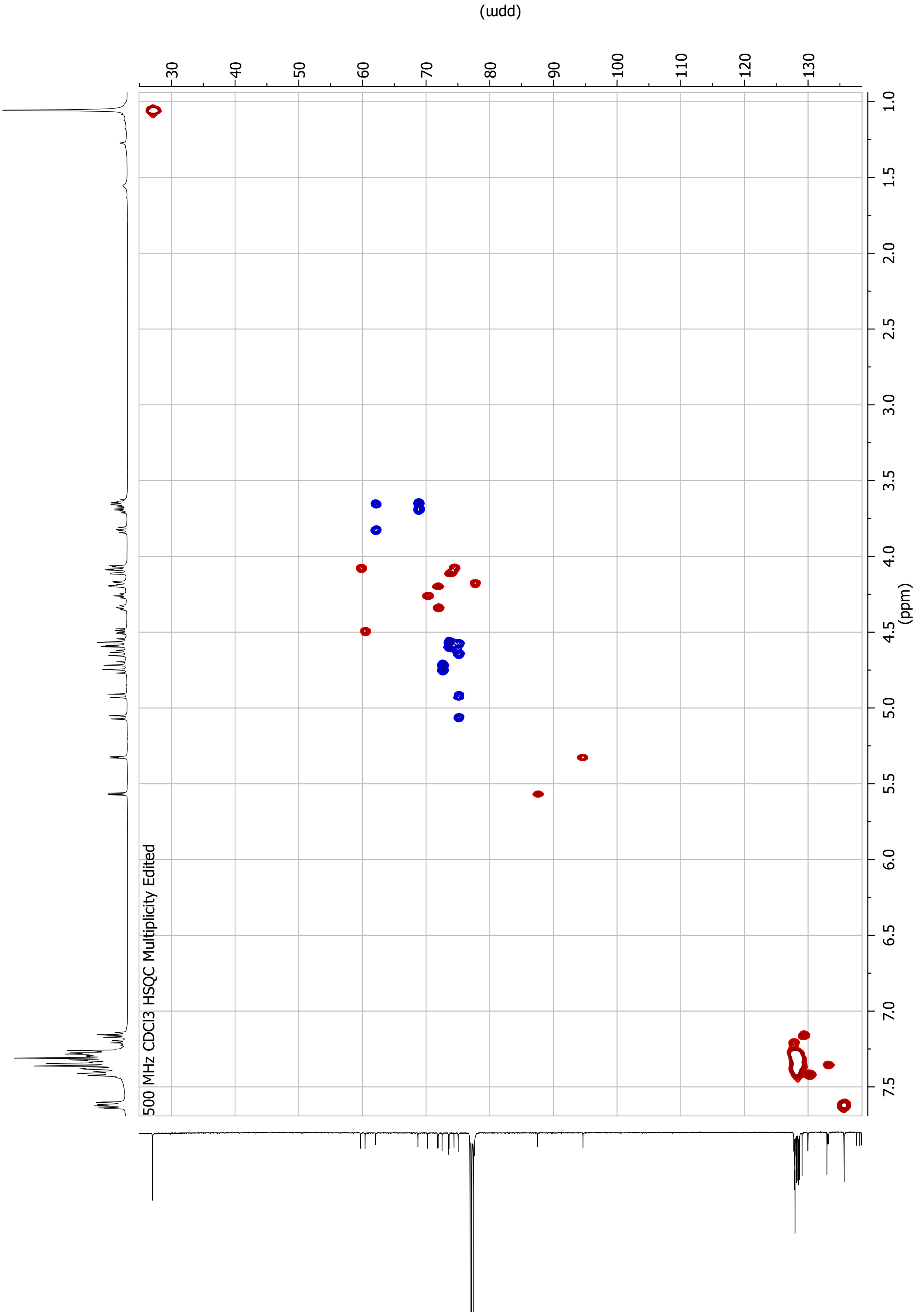

Figure S54: HSQC Multiplicity Edited NMR (500 MHz, $CDCl_3$)

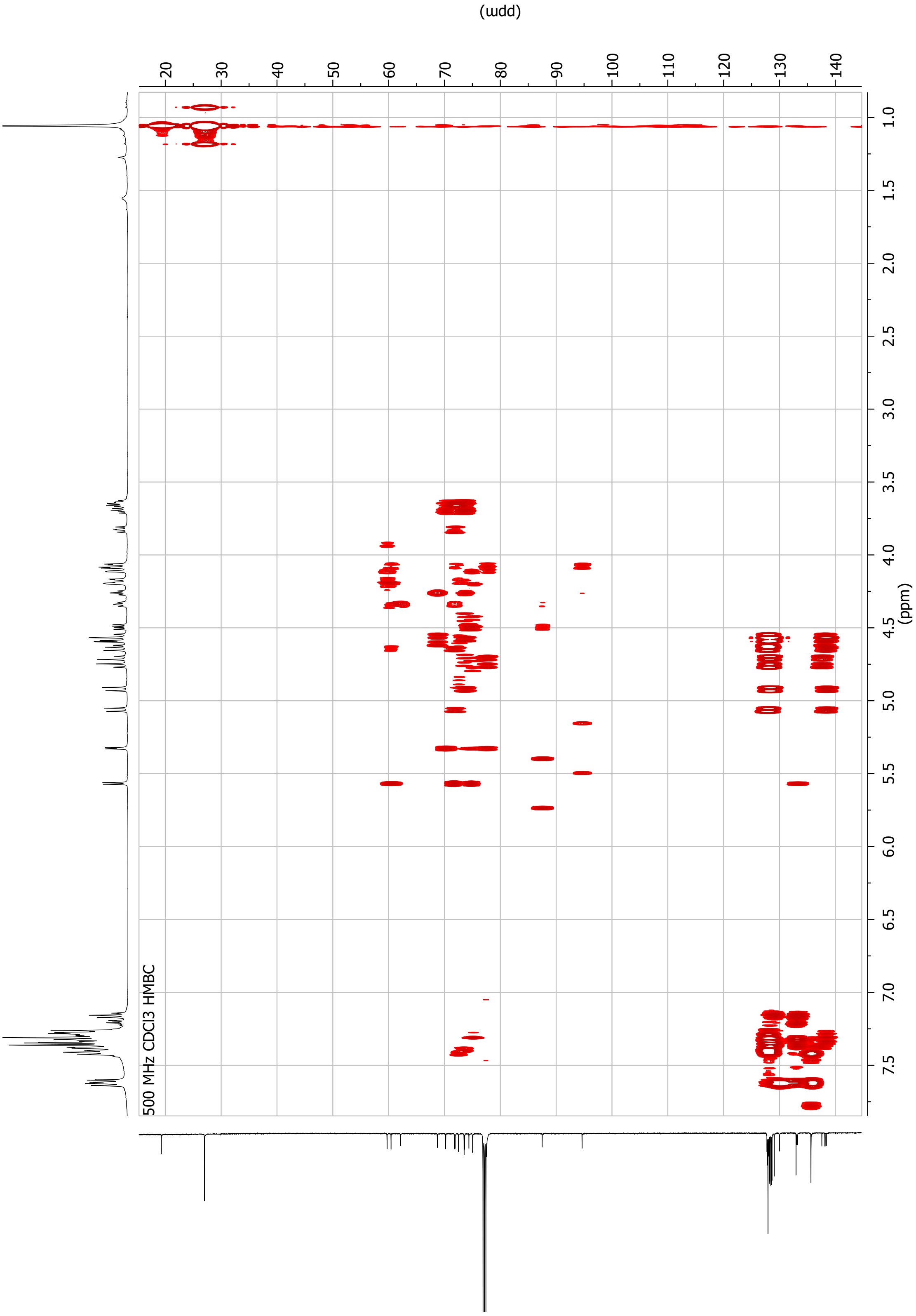

Figure S55: HMBC NMR (500 MHz, $CDCl_3$)

S2.13 Spectra for 25

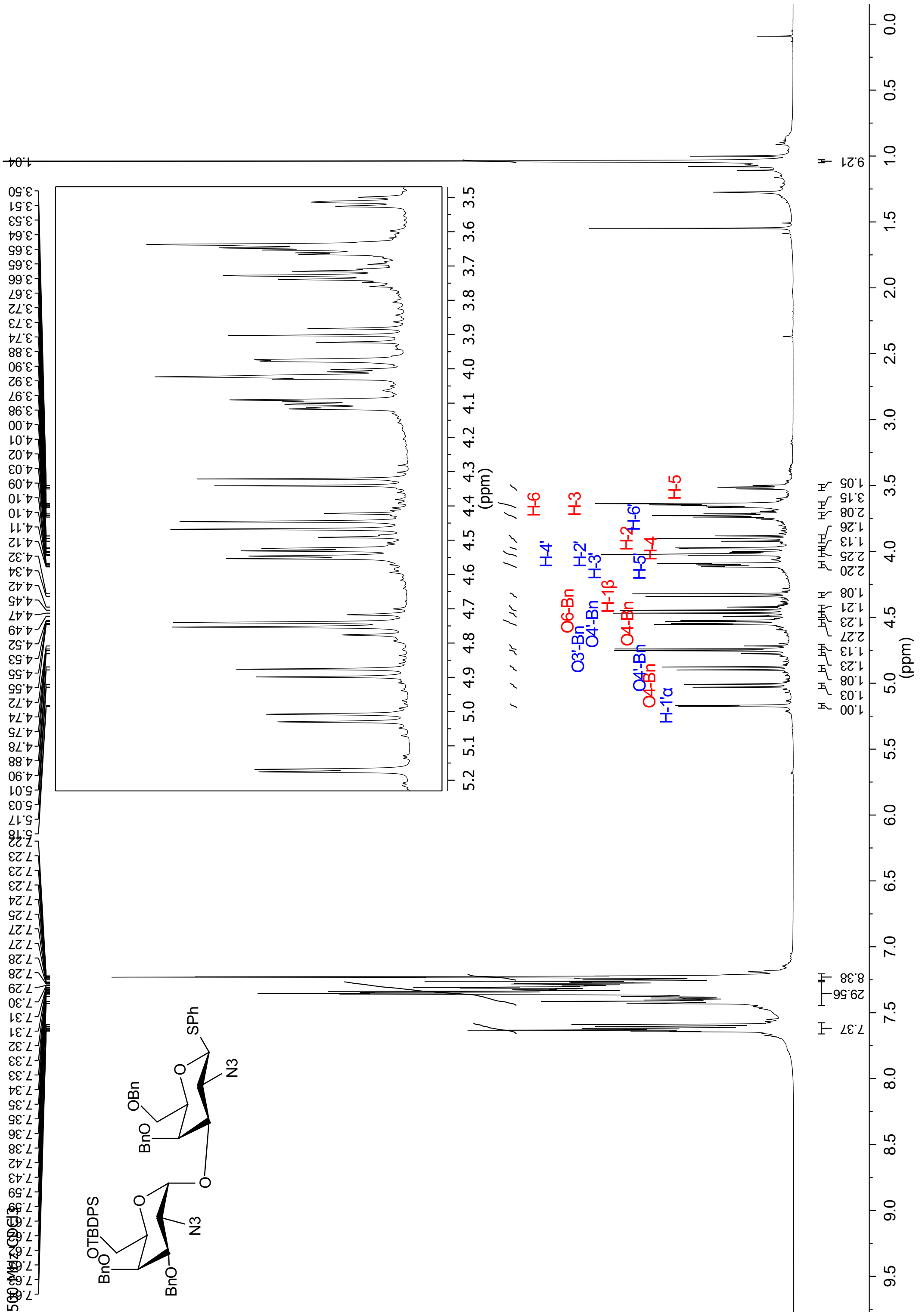

Figure S56: ^{1}H NMR (500 MHz, $CDCl_3$)

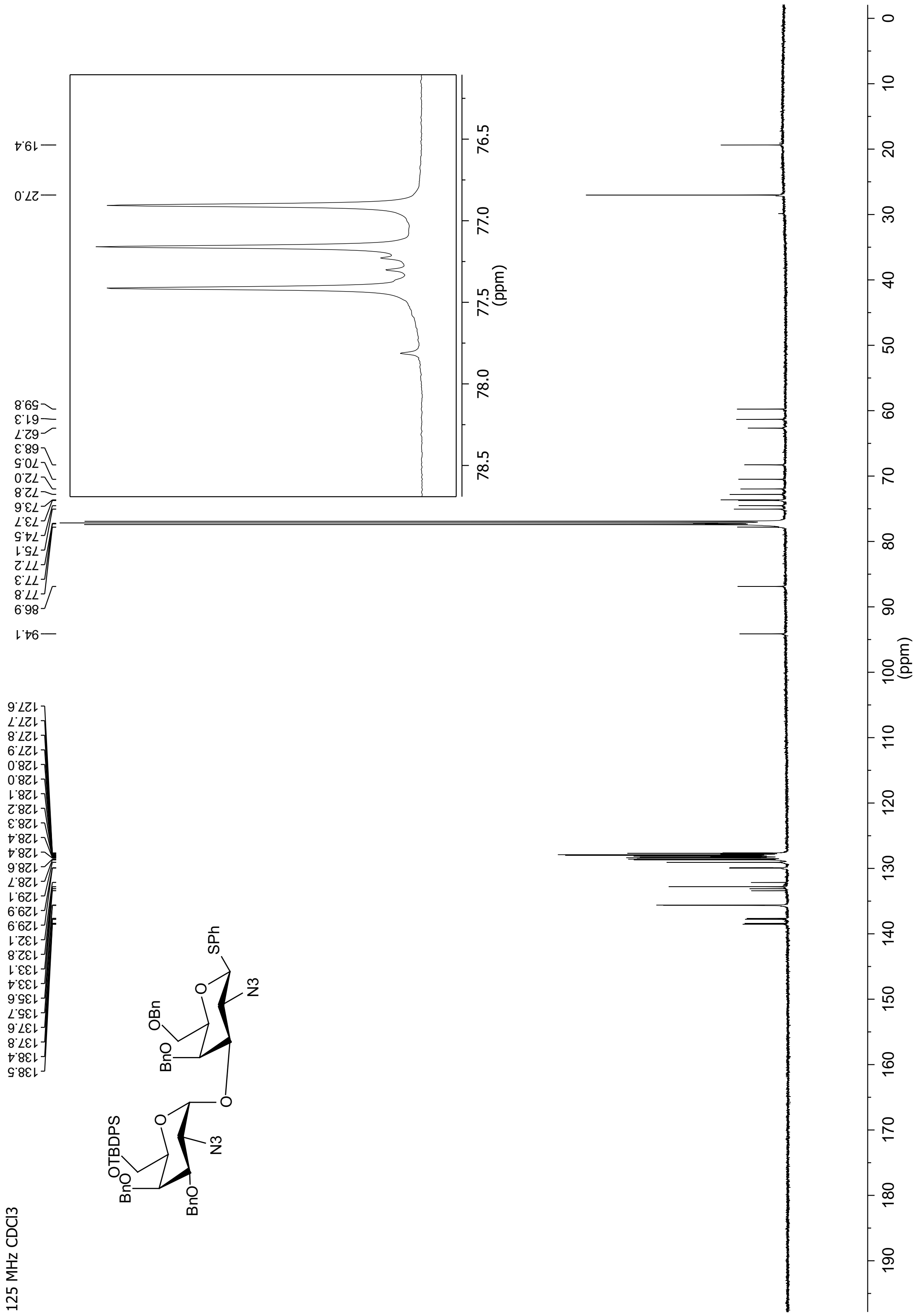

Figure S57: ^{13}C NMR (125 MHz, $CDCl_3$)

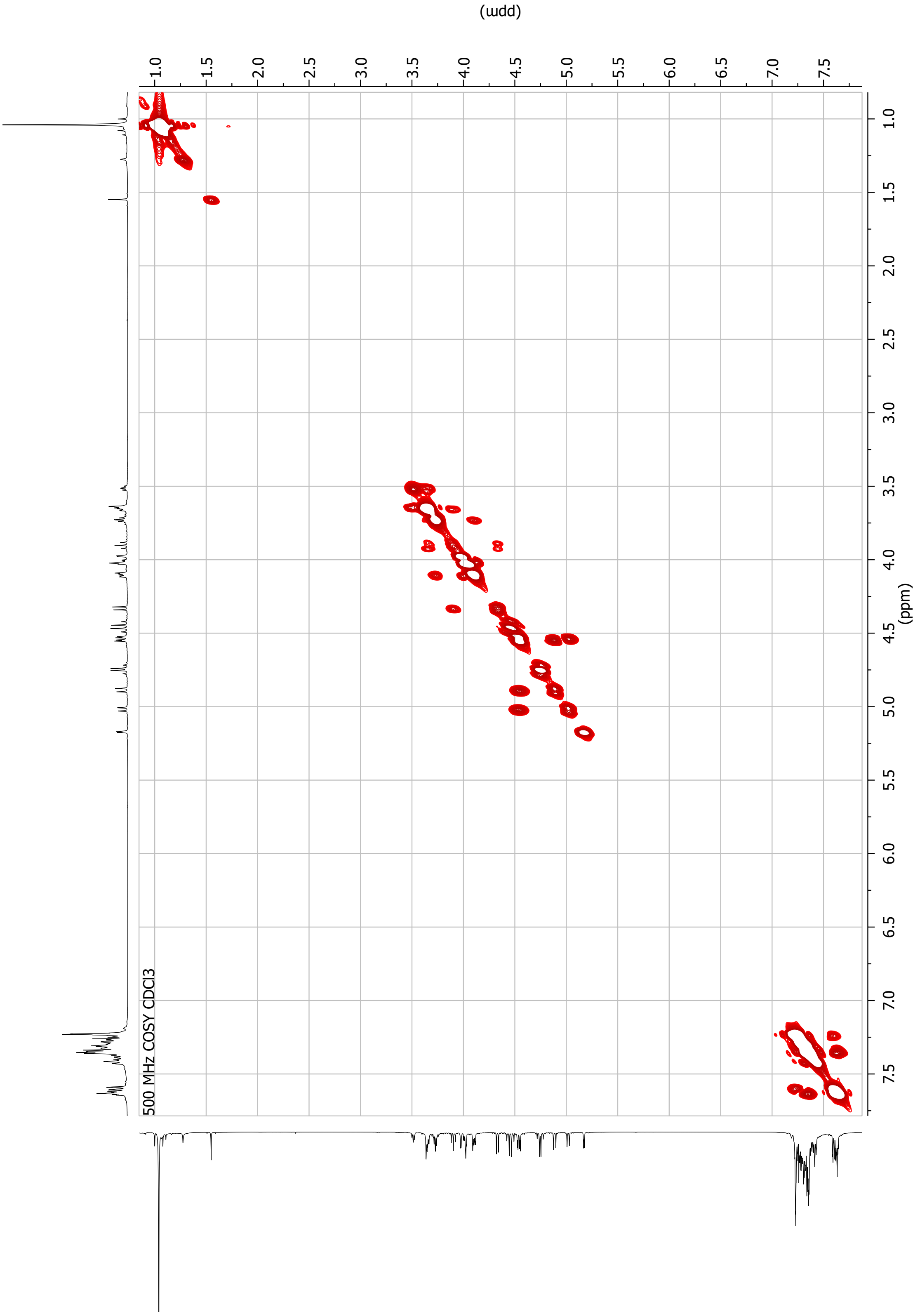

Figure S58: COSY NMR (500 MHz, $CDCl_3$)

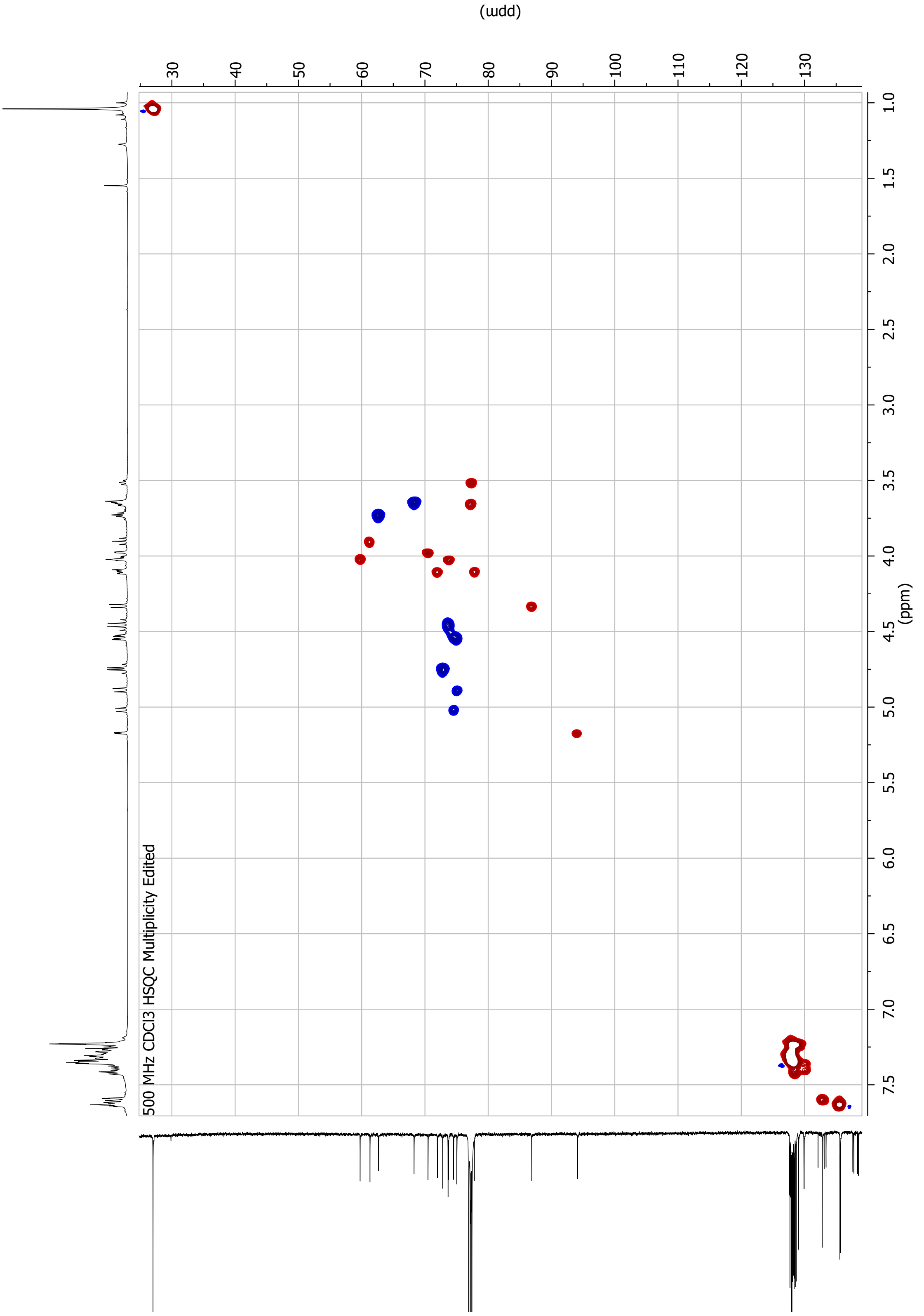

Figure S59: HSQC Multiplicity Edited NMR (500 MHz, $CDCl_3$)

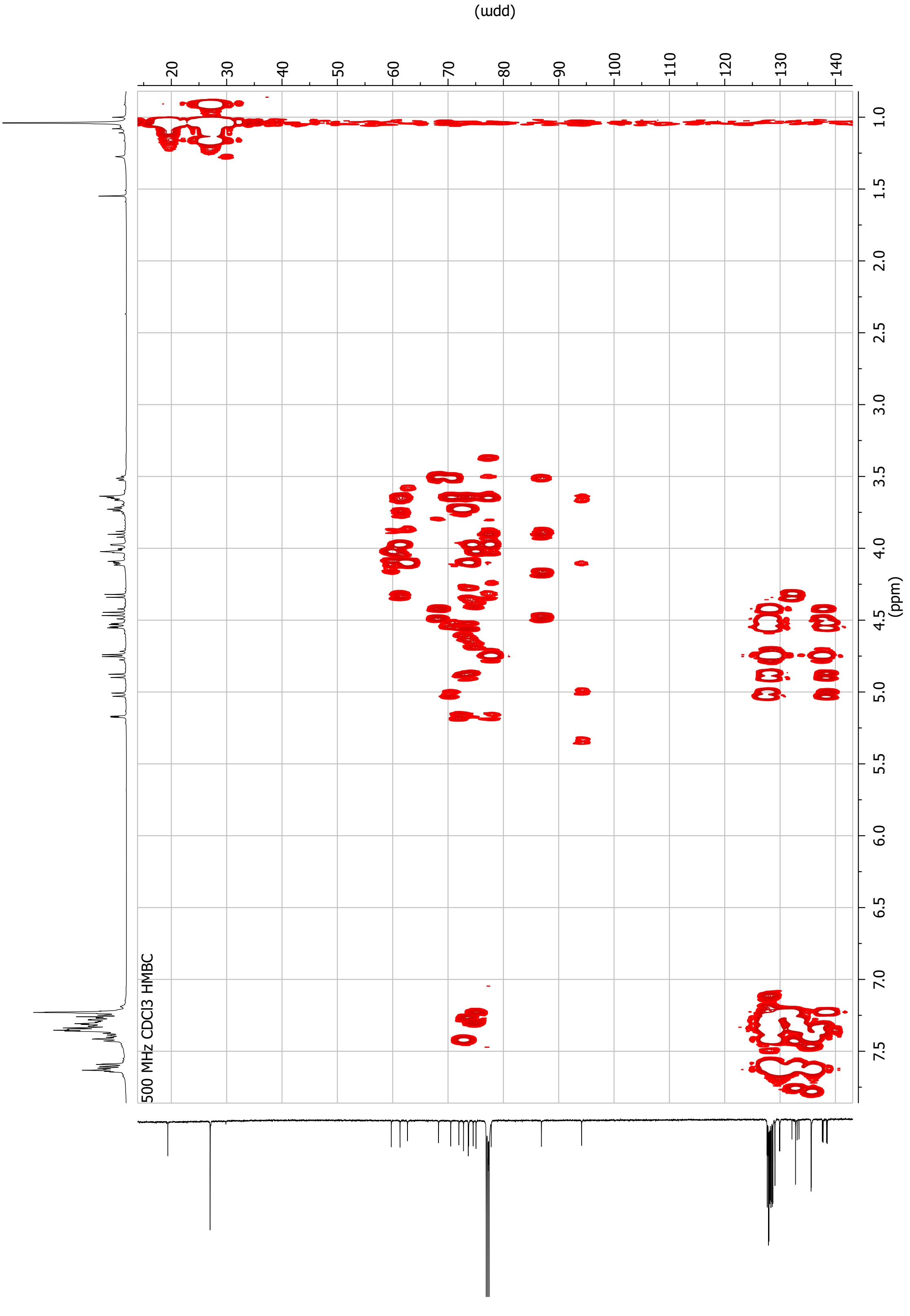

Figure S60: HMBC NMR (500 MHz, $CDCl_3$)

S2.14 Spectra for 27

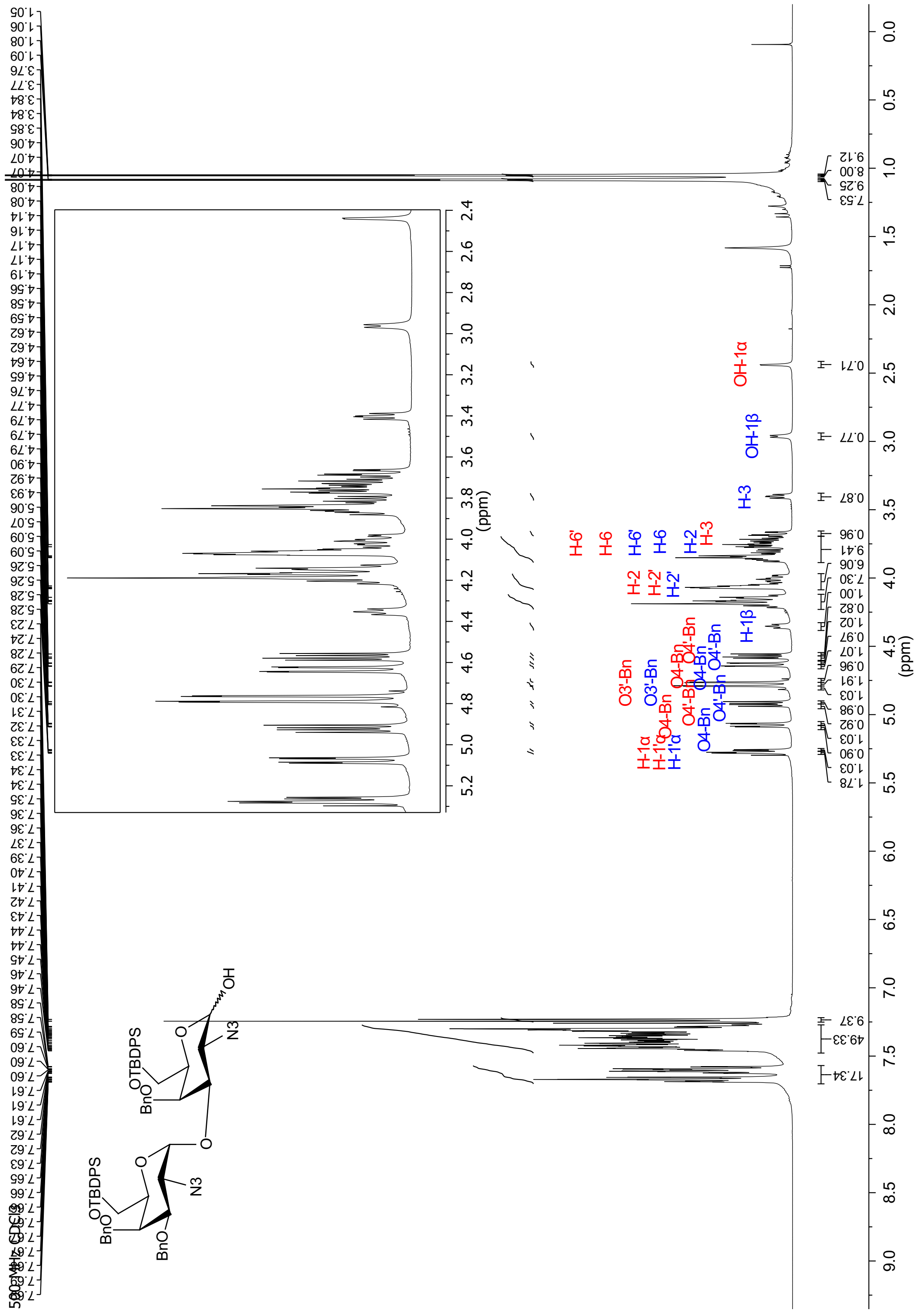

Figure S61: ^{1}H NMR (500 MHz, $CDCl_3$)

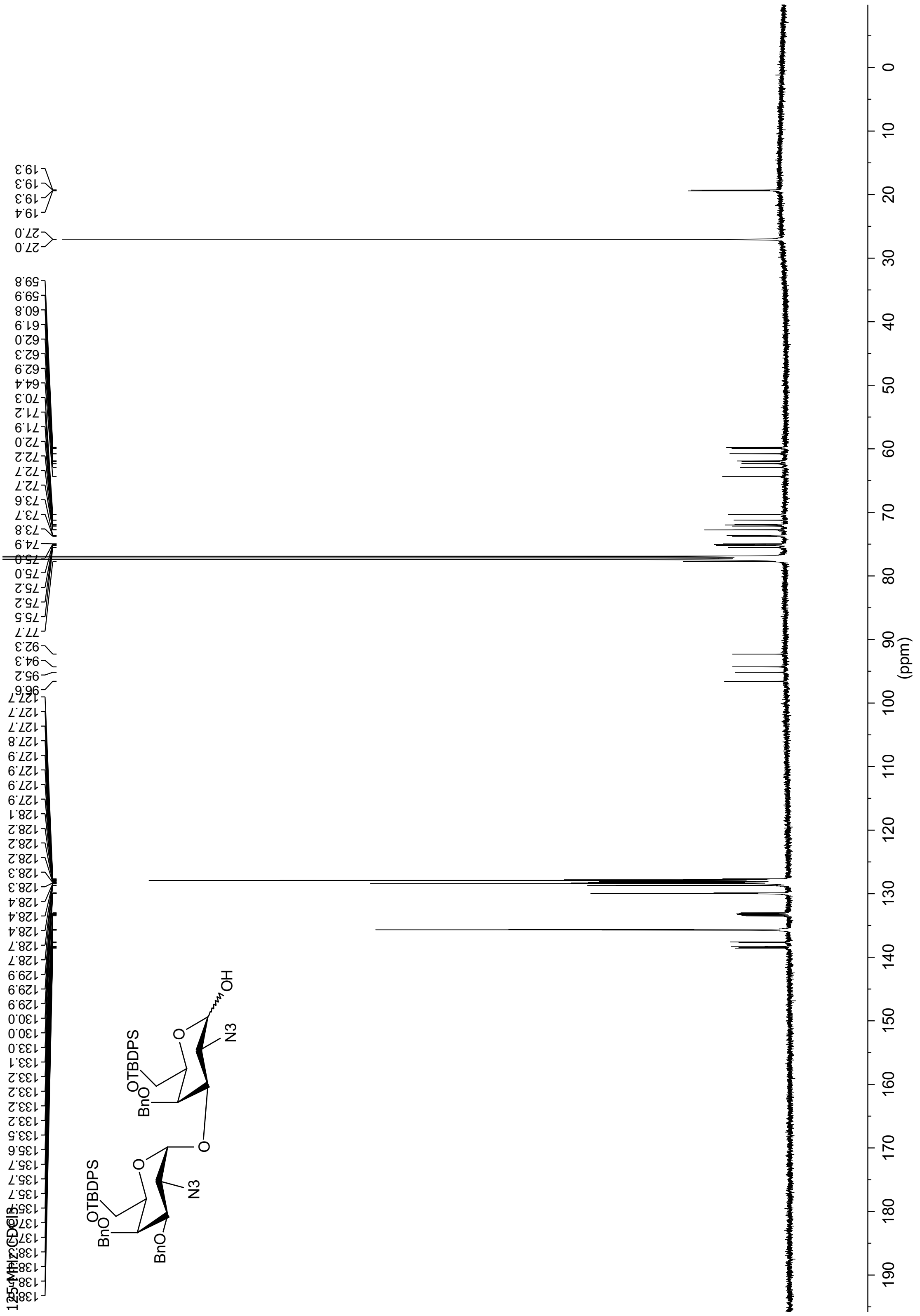

Figure S62: ^{13}C NMR (125 MHz, $CDCl_3$)

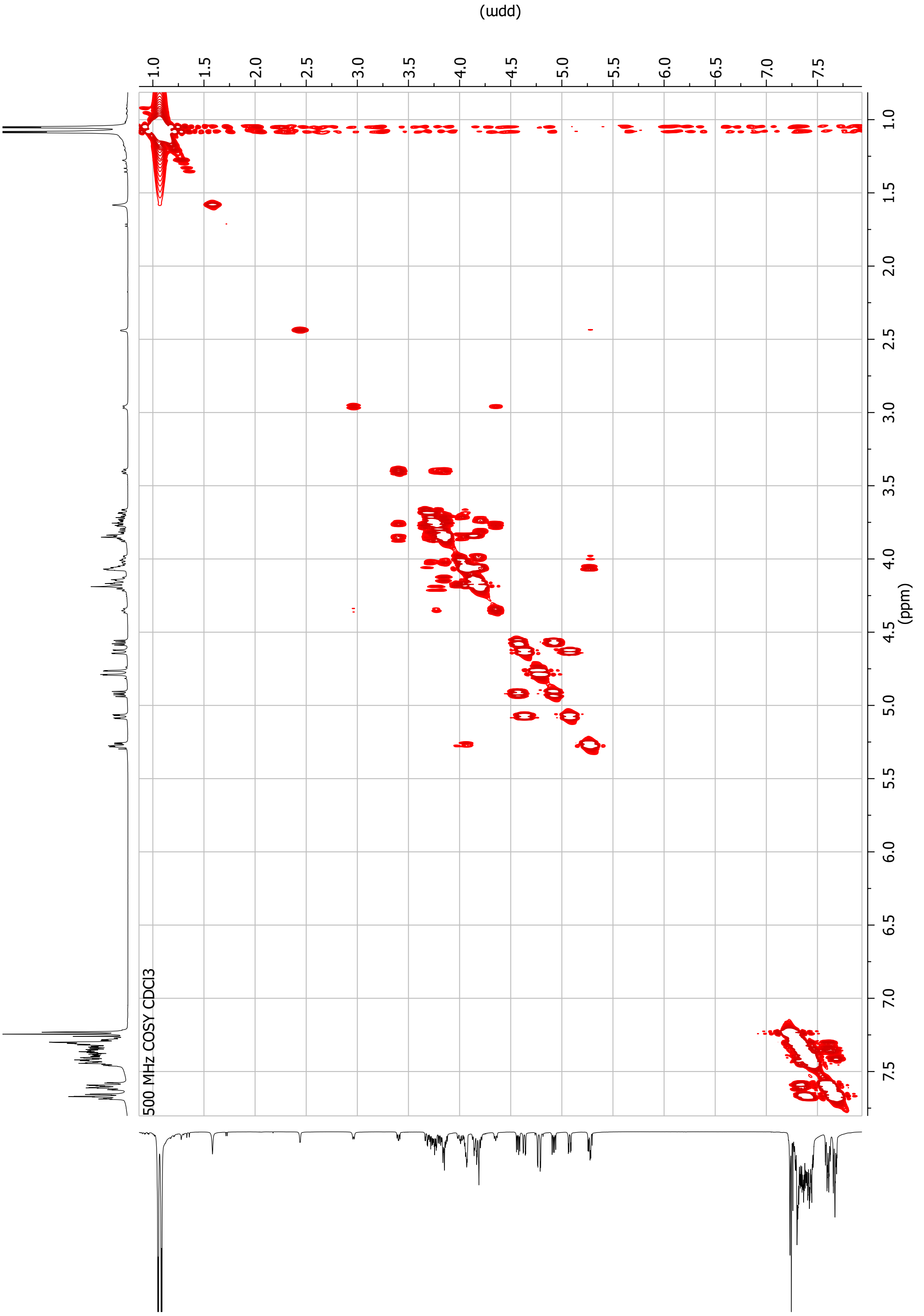

Figure S63: COSY NMR (500 MHz, $CDCl_3$)

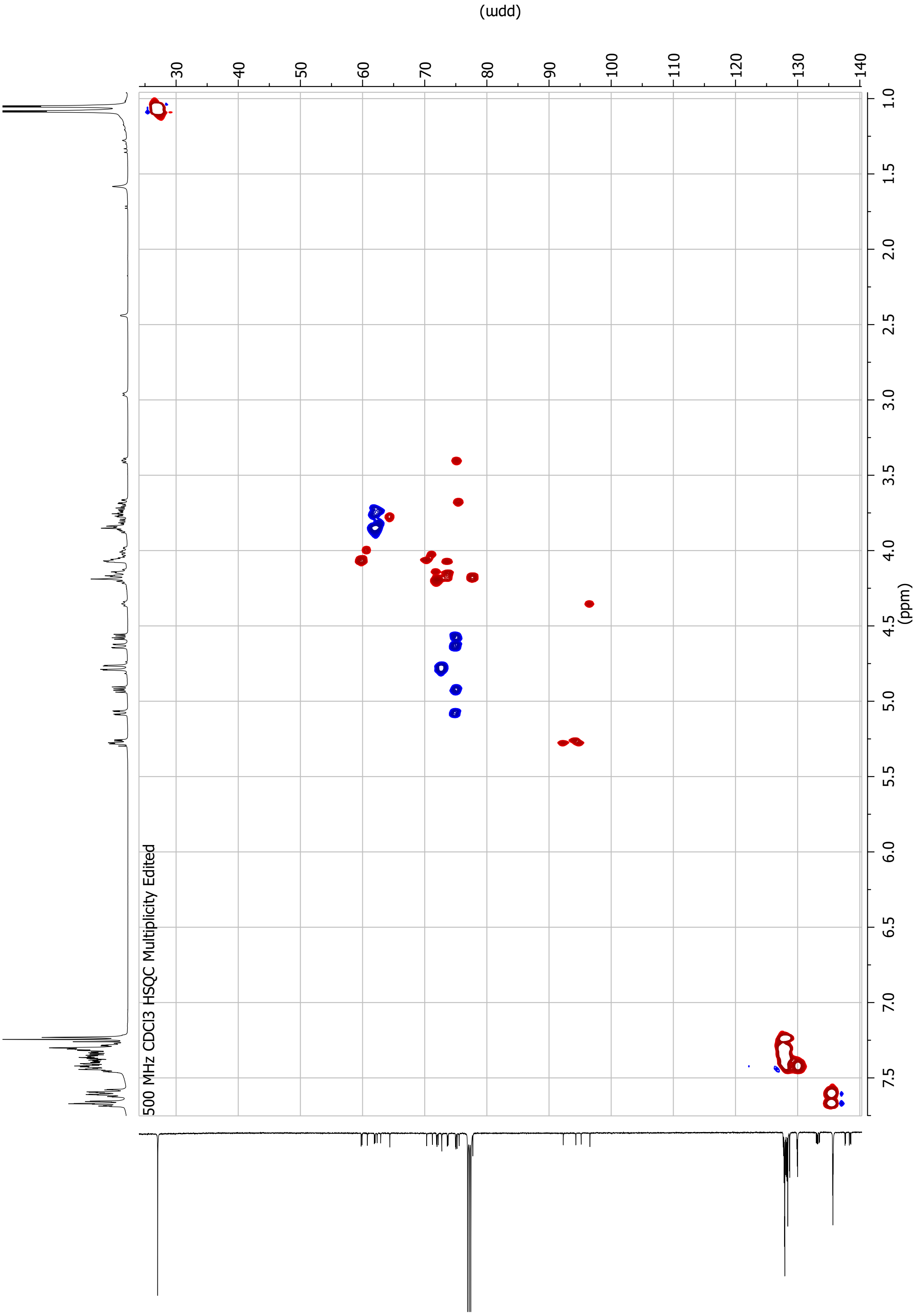

Figure S64: HSQC Multiplicity Edited NMR (500 MHz, $CDCl_3$)

S2.15 Spectra for 28

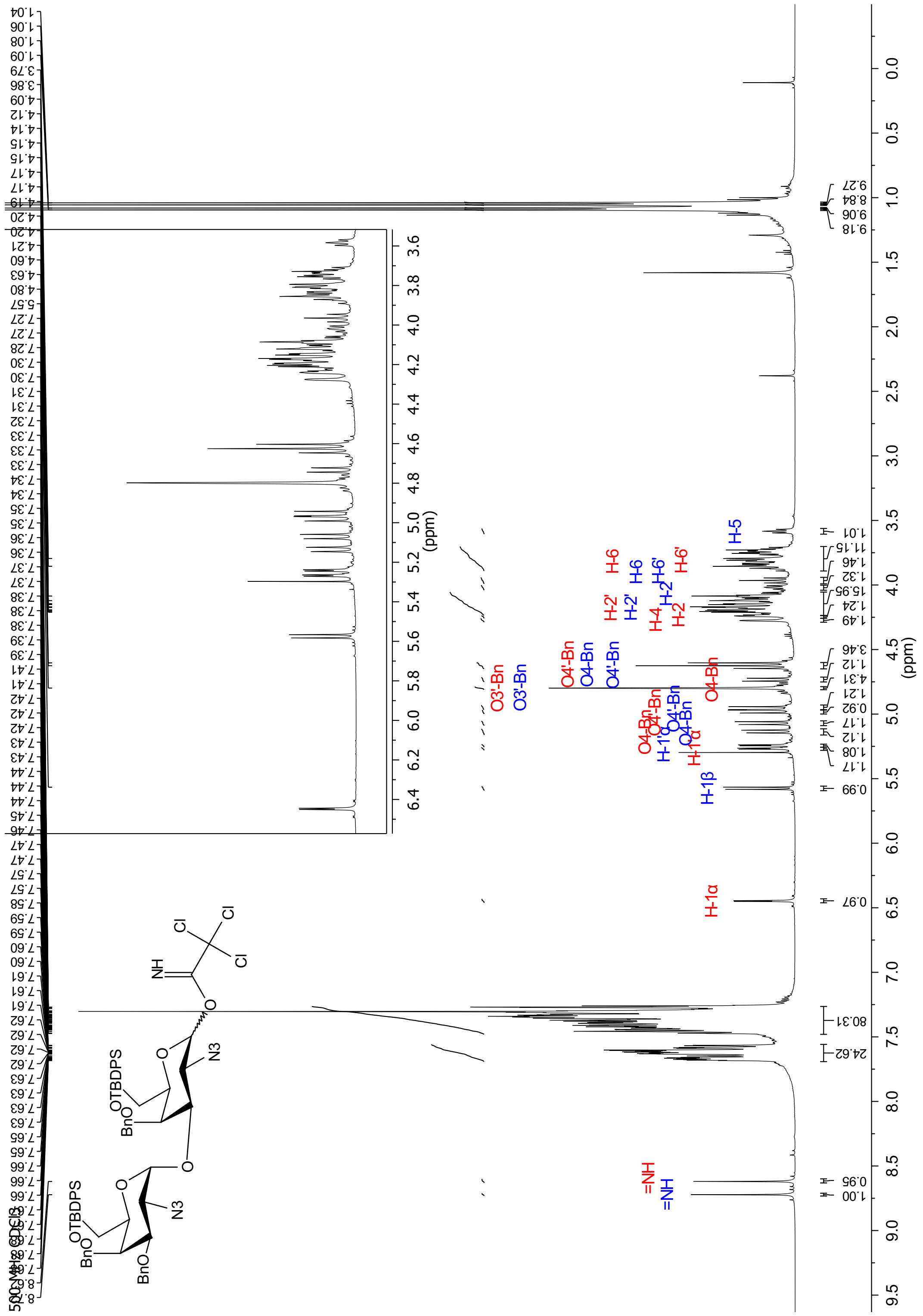

Figure S65: ^{1}H NMR (500 MHz, $CDCl_3$)

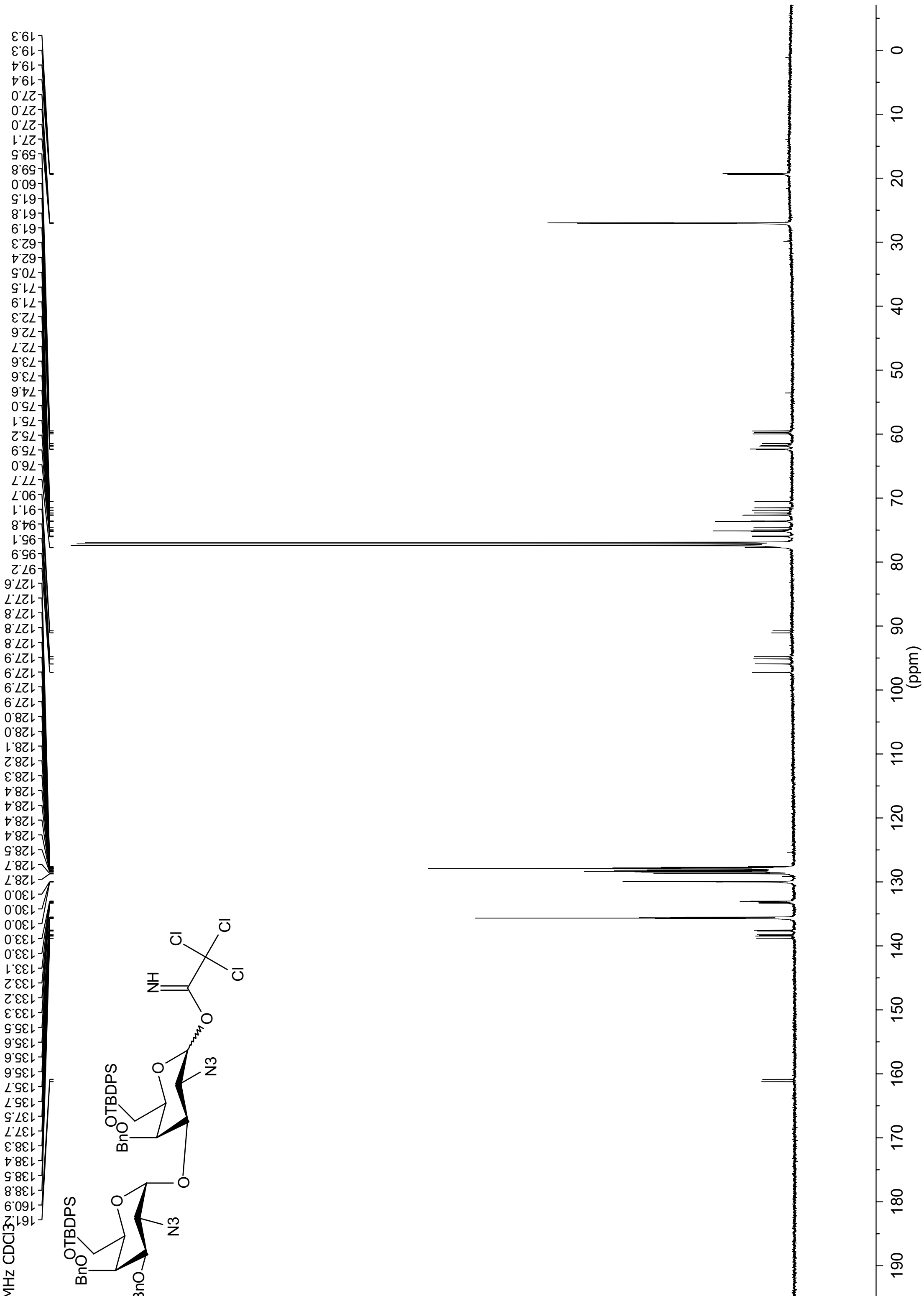

Figure S66: ^{13}C NMR (125 MHz, $CDCl_3$)

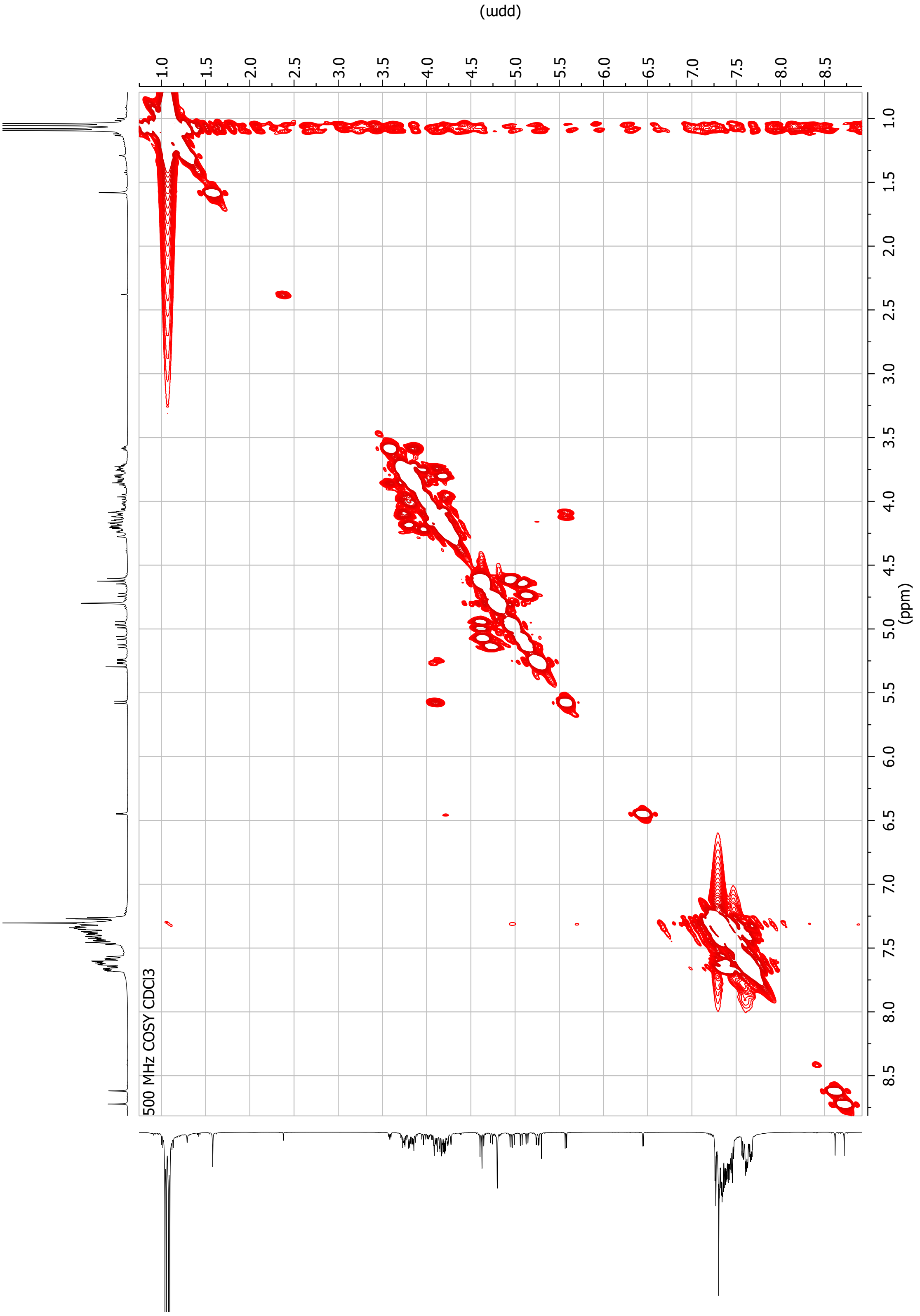

Figure S67: COSY NMR (500 MHz, $CDCl_3$)

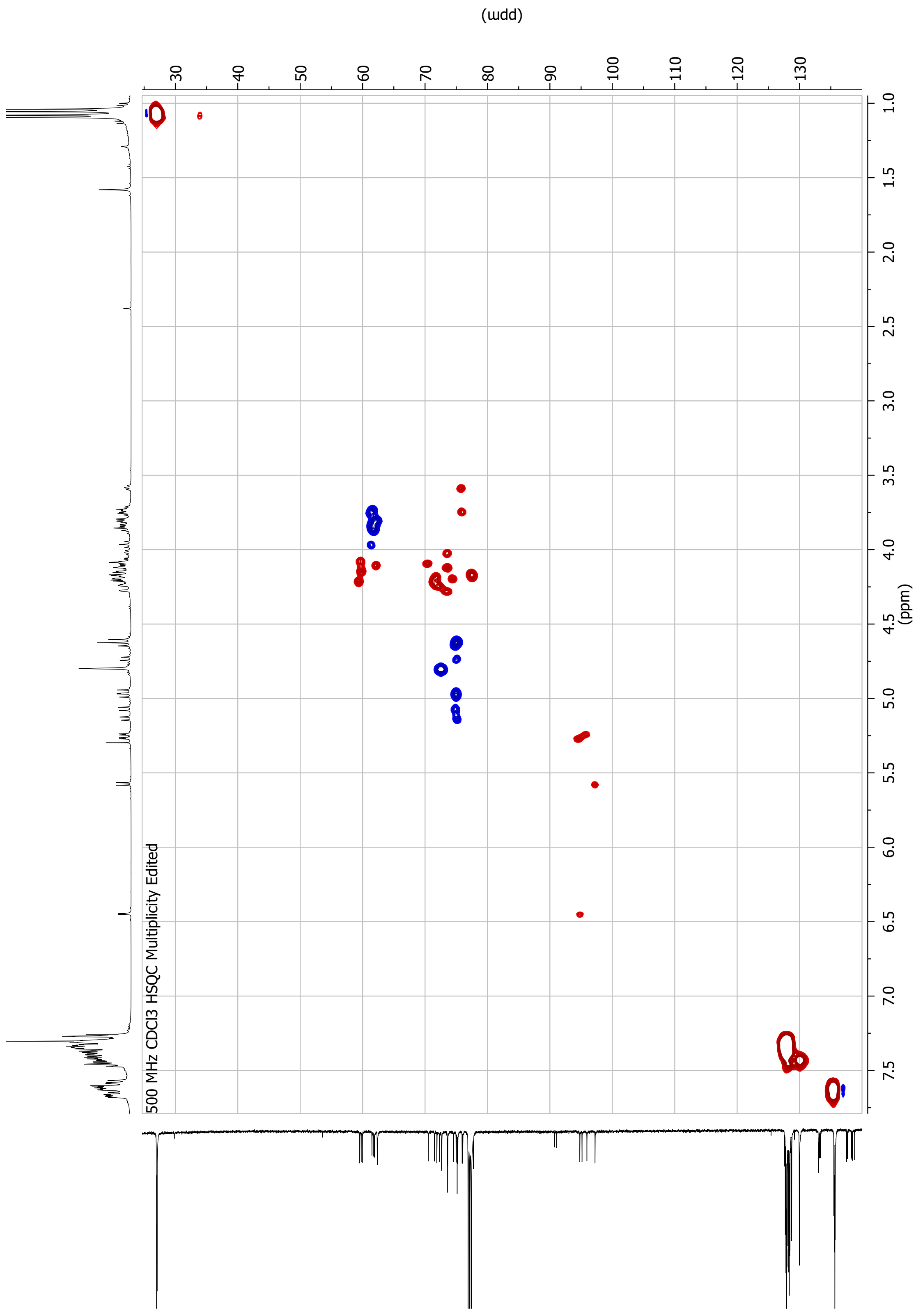

Figure S68: HSQC Multiplicity Edited NMR (500 MHz, $CDCl_3$)

S2.16 Spectra for 29

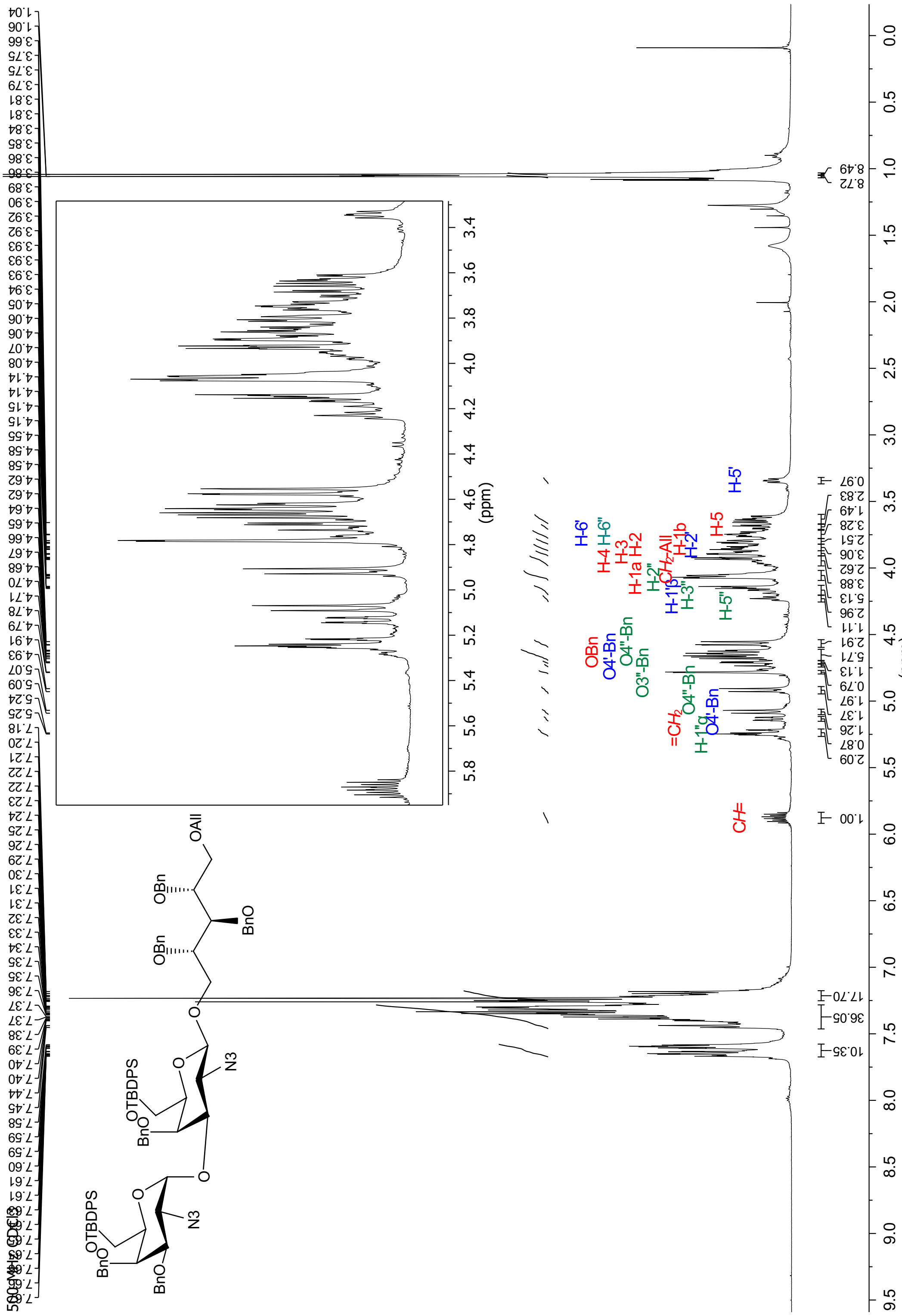

Figure S69: ^{1}H NMR (500 MHz, $CDCl_3$)

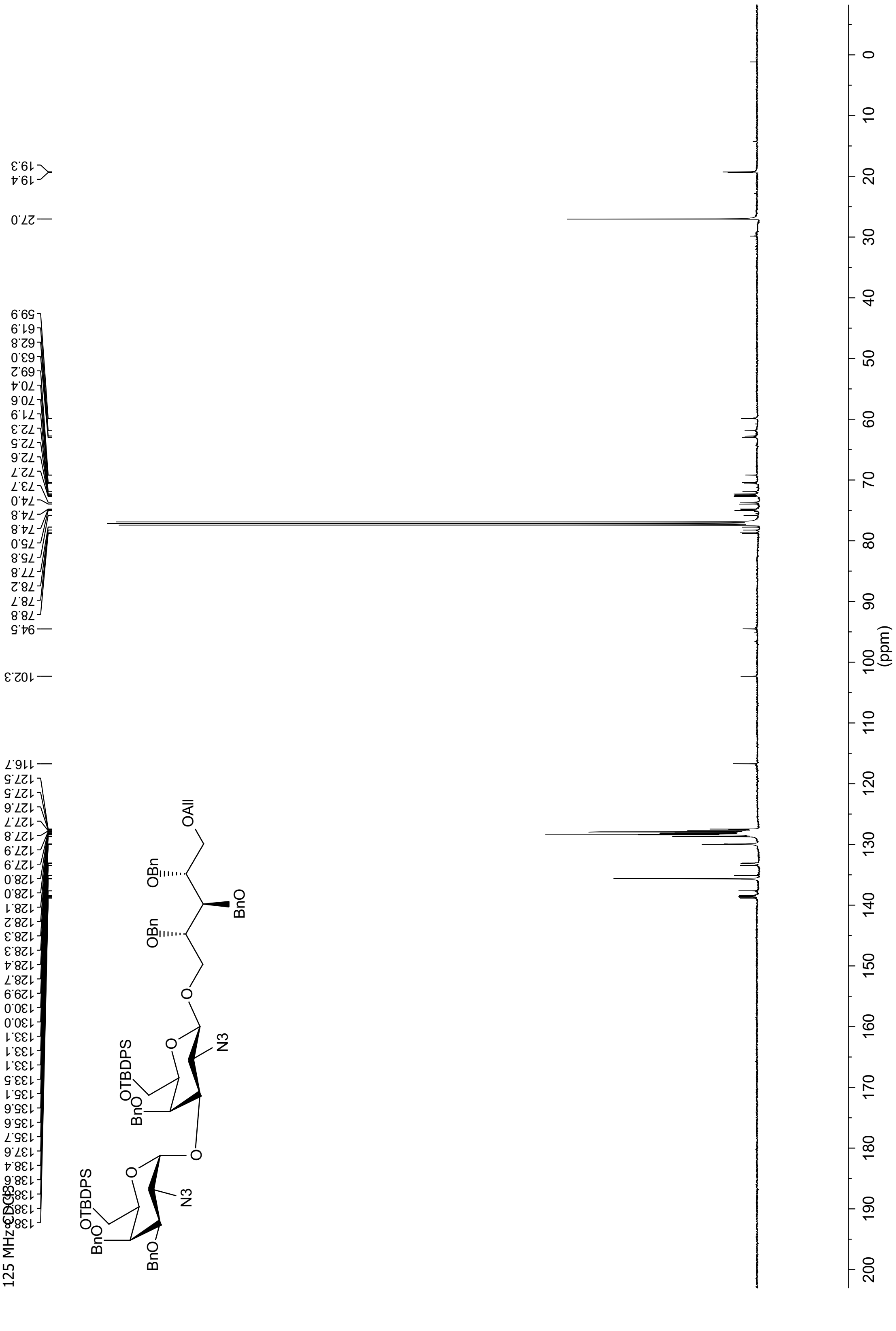

Figure S70: ^{13}C NMR (125 MHz, $CDCl_3$)

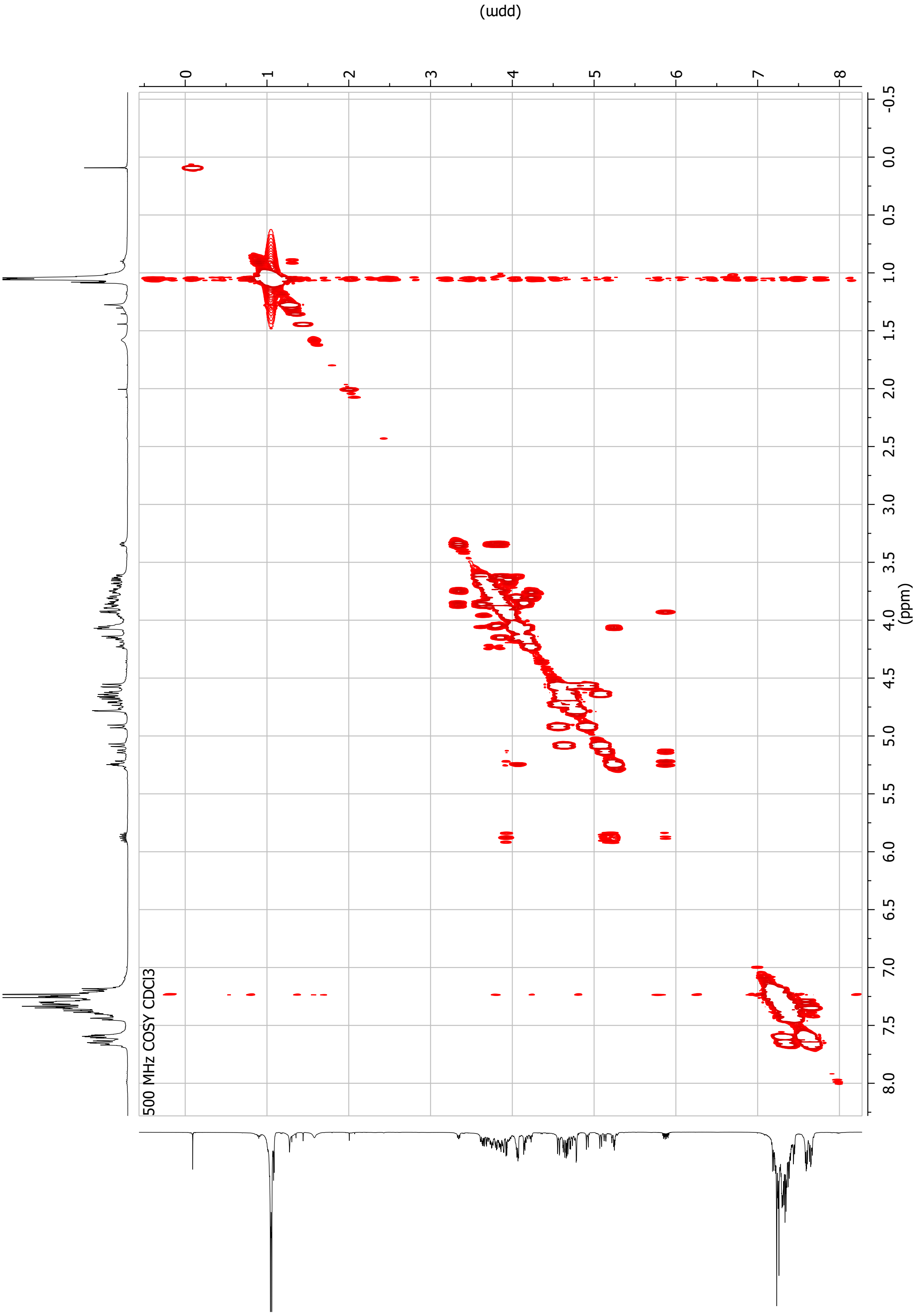

Figure S71: COSY NMR (500 MHz, $CDCl_3$)

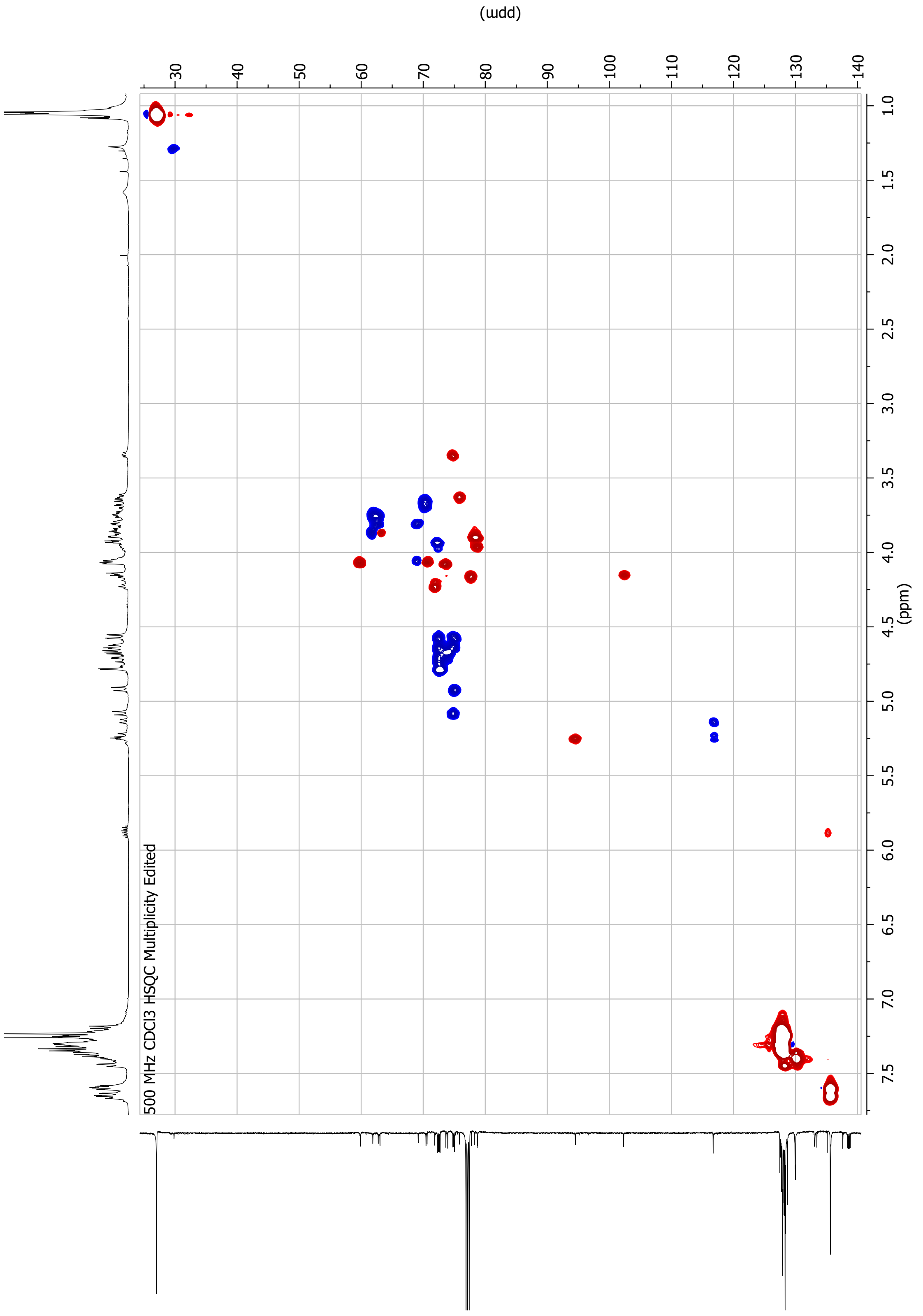

Figure S72: HSQC Multiplicity Edited NMR (500 MHz, $CDCl_3$)

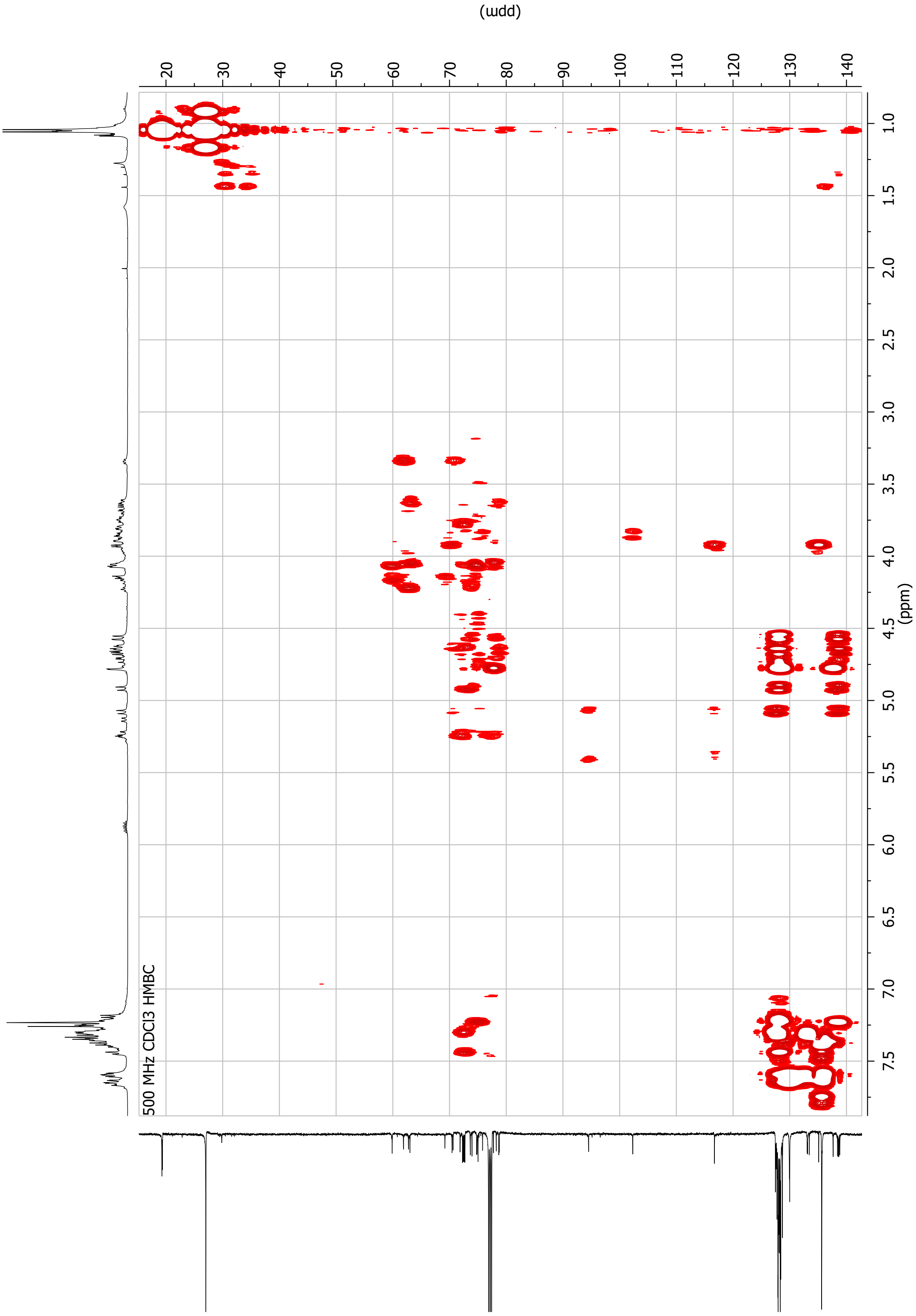

Figure S73: HMBC NMR (500 MHz, $CDCl_3$)

References

[1] Glibstrup, E.; Pedersen, C. M. *Org. Lett.* **2016**, *18*, 4424–4427.

[2] Cheshev, P. E.; Kononov, L. O.; Tsvetkov, Y. E.; Shashkov, A. S.; Nifantiev, N. E. *Russ. J. Bioorg. Chem.* **2002**, *28*, 419–429.

[3] van der Es, D.; Groenia, N. A.; Laverde, D.; Overkleeft, H. S.; Huebner, J.; van der Marel, G. A.; Codée, J. D. *Bioorg. Med. Chem.* **2016**, *24*, 3893–3907.

[4] Dere, R. T.; Kumar, A.; Kumar, V.; Zhu, X.; Schmidt, R. R. *J. Org. Chem.* **2011**, *76*, 7539–7545.

[5] Kumar, A.; Kumar, V.; Dere, R. T.; Schmidt, R. R. *Org. Lett.* **2011**, *13*, 3612–3615.

[6] Suzuki, K.; Ito, Y.; Kanie, O. *Carbohydr. Res.* **2012**, *359*, 81–91.

[7] Suzuki, K.; Ohtsuka, I.; Kanemitsu, T.; Ako, T.; Kanie, O. *J. Carbohydr. Chem.* **2005**, *24*, 219–236.

[8] Li, Z.; Gildersleeve, J. C. *Tetrahedron Lett.* **2007**, *48*, 559–562.

[9] Dileep Kumar, J. S.; Dupradeau, F.-Y.; Strouse, M. J.; Phelps, M. E.; Toyokuni, T. *J. Org. Chem.* **2001**, *66*, 3220–3223.

[10] Kalikanda, J.; Li, Z. *J. Org. Chem.* **2011**, *76*, 5207–5218.

[11] Grundler, G.; Schmidt, R. R. *Liebigs Ann. Chem.* **1984**, *1984*, 1826–1847.

[12] Qin, H.; Grindley, T. B. *Can. J. Chem.* **1999**, *77*, 481–494.
